# The effect of crop-rotation systems in regenerative agricultural environments on the rhizosphere microbiome of dry-land winter wheat

**DOI:** 10.1093/sumbio/qvaf020

**Published:** 2025-08-29

**Authors:** Lucan D Page, Johann Strauss, Karin Jacobs

**Affiliations:** Department of Microbiology, Stellenbosch University, Matieland, Stellenbosch 7600, South Africa; Western Cape Department of Agriculture, Western Cape Government, Elsenburg 7607, South Africa; Department of Agronomy, Stellenbosch University, Matieland, Stellenbosch 7600, South Africa; Department of Microbiology, Stellenbosch University, Matieland, Stellenbosch 7600, South Africa

**Keywords:** wheat cultivation, crop rotations, regenerative agriculture, agricultural droughts

## Abstract

Regenerative agriculture is a multifaceted approach that aims at transitioning farmers from conventional to sustainable management practices, by increasing biodiversity, functional redundancy, and nutrient cycling efficiency in the soil. This study investigated the effects of three crop rotation systems, wheat after wheat, wheat after medicago, and wheat after canola on the soil fungal and bacterial communities, in a regenerative agriculture system, in the Western Cape, South Africa, following a record-breaking drought (2015–19). Utilizing 16S rRNA and ITS (Internal transcribed spacer region) targeted amplicon sequences, soil-geochemical properties, and qPCR (Quantitative Polymerase Chain Reaction) analyses, it was found that crop rotations had little significant effect on the alpha-diversity between different crop-rotation systems . Medicago and wheat had the most similar communities, with canola diverging from the other rotation systems. Host driven selection was prevalent in the rhizosphere microbiome during the wheat growth period, across treatments and farms. After senescence, the microbiome composition transitioned from the wheat-selected communities towards communities consisting of saprotrophs and yeasts. qPCR analyses of nitrogen-associated genes revealed that genes for nitrification (*amoA*) and denitrification (*nirK)* increased during fertilizer application events, whereas genes associated with nitrogen fixation (*nifH)*, denitrification (*nirS*), and nitrification (*nxrB*) increased throughout the season. Genera associated with drought and halotolerance were enriched in all samples.

**Sustainability Statement**

The UN sustainability goals lay out a mandate for farmers to address not only their greenhouse gas emissions but to increase sustainable farming practices. One method utilized by farmers to address these goals is regenerative agriculture, limiting chemical inputs and mechanical disturbance of the soil to increase soil microbial activities, with the goal of increasing nitrogen use efficiency and carbon sequestration. In this study, we looked at the effects regenerative agriculture has on the rhizosphere microbiome of wheat, under drought conditions, and how changes in the microbiome could in the future be exploited to increase beneficial functionalities in the soil in alignment with the UN sustainability goals. The project can be linked to SDG 12 (responsible consumption and production).

## Introduction

The microbiome is a complex structure of interconnected microorganisms that can be thought of as an exogenous organ. It has a crucial role throughout several fundamental biological processes, such as nutrition cycling (Li et al. 2014, Nabi 2023, Zhou et al. 2023) and pathogen defence (Mendes et al. 2013, Dilla-Ermita et al. 2021, Lazcano et al. 2021) that have direct impacts on the host. While the specific roles of individual organisms in the microbiome may be unclear, the microbiome as a collective is frequently seen as more significant than the contributions of its individual components (Geesink et al. 2024). The significance comes from the overall functioning of the entire microbial community rather than the mere presence or absence of an individual organism within the community. Although the presence of an organism can suggest the potential functionality of a microbiome, its behaviour in the presence of or in competition with other microbes may be less evident. For example, if a pathogen is clearly identified in the microbiome, it could suggest the possibility of future disease. However, this may not always be true when the microbiome is in equilibrium, where pathogenicity might only emerge within a community that is out of balance, i.e. dysbiosis (DiLegge et al. 2022, Hrncir 2022).

Consequently, the process of elucidating the fundamental functionality of a microbiome can be complicated. Despite the complexity of soil microbiomes, certain actions can affect the microbiome structure and functionality. These actions can be classified into three categories. Biological factors refer to the genetic characteristics of the host organism, specifically how it interacts with the microbiome. For example, plants, by excreting certain root exudates, influence the type of bacteria and fungi found within their rhizosphere microbiome (Kawasaki et al. 2016). Another biological factor is the host's ability to be colonized, which depends on factors such as the surface area and permeability of the roots (Galindo-Castañeda et al. 2022, Gholizadeh et al. 2024), particularly in the case of endomycorrhizal fungi (Pathan et al. 2023). Second, environmental conditions, particularly the climate, can significantly impact the microbiome. Abiotic factors such as heat and drought have been demonstrated to be major factors that alter the composition of the microbiome (Asaf et al. 2017, Carlson et al. 2020, Gaete et al. 2021, Xie et al. 2021, Asghar et al. 2022). The interaction between the host and its environment is crucial in this context. When a plant is experiencing drought stress, it will exhibit a specific response in its microbiome. This mechanism may involve selecting microorganisms that are tolerant to drought, which may help the plant increase its root-soil permeability to absorb more water or protect itself from pathogens that are more likely to cause disease during periods of severe drought (Ait-El-Mokhtar et al. 2023). Third, human activities and interventions of an anthropogenic nature also impact microbiomes. For instance, agricultural practices, including tillage (Kraut-Cohen et al. 2020, Schlatter et al. 2020, Li et al. 2021, Bertrand et al. 2022, Frøslev et al. 2022), fertilizer application (Yeoh et al. 2016, Chen et al. 2019, Castle et al. 2021, Zhang et al. 2021), and fallowing (Li et al. 2023, Zhong et al. 2023), are all known to influence soil microbiomes.

Significant soil degradation has been associated with conventional, high-intensity agriculture, which is defined by practices such as soil tillage (Liu et al. 2021c), the excessive use of synthetic fertilizers and pesticides (Tripathi et al. 2020), and monocropping (Kopittke et al. 2019). These practices damage soil structure and fertility by contributing to soil erosion, nutrient depletion, and a decrease in soil organic matter (SOM) (Tsiafouli et al. 2015, Alhameid et al. 2017, Kopittke et al. 2019, Xue et al. 2022) which results in the disruption of soil microbial communities, which in turn leads to a decrease in biodiversity and resilience. In contrast, regenerative agriculture offers a paradigm shift that is intended to address these challenges by restoring the health and functionality of soil (Khangura et al. 2023). This method prioritizes the implementation of practices such as the use of cover crops, reduced or no tillage, crop rotation, and organic amendments, which collectively improve soil structure, promote microbial diversity, and increase SOM (Newton et al. 2020, Luján et al. 2021, Leu 2023, Singh et al. 2023). The objective of regenerative agriculture is to rebuild soil ecosystems, enhance soil productivity and environmental health, and increase resilience to erosion and degradation by promoting a more comprehensive and sustainable interaction between soil, plants, and microorganisms.

In this study, we investigated the influence of regenerative agriculture practices on the soil rhizosphere microbiome, while maintaining a conventional monocrop rotation system. The focus of the investigation was to study the effects of practices such as reduced fertilization and no-tillage on the microbial communities in the rhizosphere of wheat that is subjected to three fixed, commonly used, monocrop rotations. These included wheat after wheat (WW), wheat after canola (WC), and wheat after medicago (WM).

While crop rotation in a regenerative agricultural setting was the primary focus of this study, it should be noted that the Western Cape region of South Africa experienced a severe and lengthy drought preceding and during the study period (2019). The main drought period spanned from 2015 to 2019, with below average rainfall persisting into 2020 (Department of Agriculture, Forestry and Fisheries 2020, Theron et al. 2021). This drought had a major negative impact on the agriculture industry in the region (Theron et al. 2021). The prolonged drought decreased soil moisture, which is vital for the survival and functioning of soil microorganisms.

In light of this context, this study proposes the following hypotheses: (i) Bulk soils will have diverse and distinct microbiome profiles. (ii) Once the wheat crop is introduced, a host selected microbiome profile will be observed with distinct characteristics associated with the previous crop in the rotation system. (iii) Drought resistant microbial genera will be prominent in the microbiome profiles.

## Materials and methods

### Location

Two sampling locations were chosen to investigate the effects of crop rotation on the soil microbiome of winter wheat (*Triticum aestivum*). These were Langgewens research farm, outside of the town Malmesbury, South Africa (33°16′37.4″S, 18°42′10.4″E) and Tygerhoek research farm, located outside of the town Riviersonderend, South Africa (34°09′58.6″S, 19°54′40.7″E).

### Sampling protocol

Soil samples were collected from both bulk and rhizosphere soils. Bulk soils consisted of soils found surrounding the plant, separate from the crop roots. Bulk soils were used for soil chemical analyses as well as the establishment of the pre-plant (PP) baseline microbial communities in the absence of plants. After removing the upper SOM layer, 2 kg of bulk soil was sampled at each sampling point, up to 30 cm in depth. Rhizosphere soils consisted of soils found in the immediate vicinity around crop roots within the rhizosphere. Around 500 g of soil surrounding the roots of each sampled plant was collected. One gram of the rhizosphere soil was subsampled from the core root soil, which was used in this study for the purposes of DNA extraction and targeted amplicon sequencing.

Before soil samples were taken, all sampling equipment (shovels and sheers) were sterilized using 70% ethanol. Sterilization of equipment also occurred between each sampling point. Once the sampling equipment had been sterilized, the top layer of soil, consisting of SOM, crop residues, and livestock faecal matter, was removed. Plants were carefully uprooted to ensure the roots and surrounding soil remained intact. Approximately 1 kg of bulk soil was sampled from the immediate vicinity of the removed plant and placed into a separate sampling bag. Samples were stored at 4°C for up to 48 h until downstream analysis was completed.

### Soil chemical analyses

Soil chemical analyses, consisting of pH, resistance (EC), sodium, potassium, calcium, magnesium, carbon, phosphorus, Bray II, titratable acidity, and stone percentage, were conducted as part of the BemLab Standard analyses at Pathcare BemLab (Pty) Ltd (BemLab, SA) (Pathcare Bemlab 2019). Further soil chemical and enzyme analyses on the rhizosphere soil was performed at the Department of Conservation Ecology and Entomology, Stellenbosch University. These analyses consisted of pH, soil moisture, available phosphorus, nitrates (Baillie et al. 1990), ammonium (T0) vs ammonium (T7), nitrogen mineralization (Baillie et al. 1990), urease activity, alkaline and acid phosphatase activity, as well as beta-glucosidase activity (Dick et al. 2015).

### Wheat cultivars

Wheat cultivars were farm-specific and selected by the individual farm managers based on their respective requirements. The wheat cultivar grown on Langgewens was SST0166 [Sensako (PTY) LTD, 2018]. Similarly, the cultivar grown at Tygerhoek was SST0127 [Sensako (PTY) LTD, 2012]. These crops are transgenic; however, the specific extent of genetic modification remains proprietary information held by the seed company.

### Crop rotation systems (treatments)

For the purposes of this study, three monocrop crop-rotation systems were evaluated. To determine the crop rotation categories, the sampling year (2019) and the previous year (2018) were considered. Treatments were characterized into four main categories, namely (*n* = number of fields per farm):

- WW (4n)—Langgewens (2n) and Tygerhoek (2n).
- WC (*Brassica napus*) (4n)—Langgewens (2n) and Tygerhoek (2n).
- WM (*Medicago sativa*) (4n)—Langgewens (2n) and Tygerhoek (2n).

WW was chosen as the control for conventional farming strategies which often utilize monocrop systems. Consequently, WC and WM were chosen as our regenerative agriculture rotation systems, utilizing conventional cash crops, such as canola, and crops utilized for soil nutrient replenishment, such as legumes (*Medicago*). Samples were grouped into three categories, namely, PP—the period before seeding, the wheat growth period (WGP), combining the sampling points at-germination (GM), 2 weeks after the first wheat seeds started to germinate, top-dressing (TD), 2 weeks after additional fertilizer was added to the crops; and lastly, at-harvest (AH), after the wheat drying period, before harvest (Fig. S1).

### Farm metadata

Farm metadata such as seasonal rainfall, fertilizer type and quantity, and harvest quantity and quality were gathered from the farm managers. Rainfall data were compared to the area's average, and it is important to note here that the 2019 wheat growing season was at the height of the Western Cape drought, which occurred between the years 2015 and 2020 (Agri SA Research, 2019).

### EMA treatment and DNA extractions

Before DNA extraction, extracellular DNA was removed using 25 μM ethidium monoazide bromide (EMA) (Thermo Fisher Scientific, USA) consisting of 20% dimethyl sulfoxide (Thermo Fisher Scientific, USA), using a modified treatment method as described by (Wagner et al. 2015). EMA solution was added to 0.3 g of soil, to account for potential sample loss during wash steps. Once EMA was added to the samples, they were incubated in the dark on a Vortex-Genie 2 (Thermo Fisher Scientific, USA) for 15 min. Subsequently, samples were exposed to a 500-watt halogen light set 20 cm above the sample plane for 15 min. Samples were exposed to light/dark cycles for two rounds. Samples were kept on ice, while under the halogen light to prevent overheating (Ditommaso et al. 2015). Following EMA treatment, DNA from 144 soil samples (0.25 g of soil per sample) was extracted using the Quick-DNA Faecal/Soil Microbe Kits (Zymo Research, USA), according to the manufacturer's instructions. Successful DNA extractions were verified on a 1% agarose gel stained with ethidium bromide. DNA was stored at −20°C until downstream application.

### Targeted amplicon PCR

Targeted amplicon PCR amplification of the extracted DNA was performed with primers targeting the 16S rRNA V4–V5 region of bacteria, namely 338-F (5′-ACT CCT ACG GGA GGC AGC AG-3′) (Ampe et al. 1999) and 783-R (5′-CTA CCA GGG TAT CTA ATC CTG-3′) (Sakai et al. 2004). For fungi, primers targeting the ITS small subunit-5.8S region, namely ITS1F-F (5′-CTT GGT CAT TTA GAG GAA GTA A-3′) (Gardes and Bruns 1993) and ITS2-R (5′- GCT GCG TTC TTC ATC GAT GC-3′) (Monard et al. 2013) were used. Each PCR consisted of 1.5 mM MgCl_2_, 0.5 μl of a 10 mM dNTP mix, 0.25 μM forward and reverse primers, 0.5 U KAPA Taq Hot Start DNA Polymerase (Sigma-Aldrich, USA), and 1.5 μl or 100 ng of template DNA.

PCR amplifications were performed on a 2720 Thermal Cycler (Thermo Fisher Scientific, USA) under the following conditions: for 16S rDNA amplification an initial denaturing at 95°C for 5 min, followed by 35 cycles of 95°C for 30 s, 58°C for 30 s, and 72°C for 1 min. A final extension was completed at 72°C for 7 min and the PCR samples were held at 4°C (Ampe et al. 1999, Sakai et al. 2004). For ITS DNA amplification an initial denaturing at 95°C for 5 min, followed by 35 cycles of 95°C for 30 s, 56°C for 30 s, and 72°C for 45 s, with a final extension at 72°C for 7 min and the PCR samples were held at 4°C (Monard et al. 2013). PCR amplification products were confirmed on a 1% agarose gel, stained with ethidium bromide. Each sample was amplified in triplicate for bacteria (16S) and for fungi (ITS) to account for PCR amplification biases that may occur. Bacterial and fungal PCR amplification replicates were pooled, separately for each sample, and stored at 4°C. Field sample replicates were analysed as independent samples, with pooling performed only at the level of PCR replicates.

### Targeted amplicon sequencing

PCR products, consisting of pooled 16S and ITS products, were sent to the Central Analytical Facility (CAF) (Stellenbosch, SA) at the University of Stellenbosch for quality control (QC) and sequencing. Concentrations and sizes of the PCR products were verified using the Lab Chip GX Software, v.5.4 (PerkinElmer Inc., UK). Following QC, 16S and ITS samples were tagged and pooled. Samples were then loaded onto an Ion 530™ Chip for sequencing using the Ion S5 Sequencing System (Ion Torrent, Thermo Fisher Scientific, USA).

### Data clean-up and statistics

Data clean-up, clustering, alignment, and operational taxonomic unit (OTU) classification were done using MOTHUR (version 1.44.1) on the high-performance computing cluster 2 at Stellenbosch University (2008), using a modified version of the MiSeq standard operating procedure (Kozich et al. 2013). 16S rDNA sequences were aligned and classified using the SILVA (version 132) full-length sequences and taxonomy reference file (Quast et al. 2012, Yilmaz et al. 2014, Glöckner et al. 2017), with a classification cut-off of 80%, and distance level OTU classification labelled at 0.03. ITS DNA sequences were aligned and classified using the UNITE (version 8.3) fungal species reference file for MOTHUR (Abarenkov et al. 2021) with a classification cut-off of 60% and distance level OTU classification labelled at 0.05.

Graphs in this section were generated, and statistical analyses were calculated using R-studio (version 4.4.2) following the R *microeco* package (version 1.5.0) (Liu et al. 2021a). Datasets were rarefied using the rarefy_samples command in R, to ensure adequate coverage of the entire microbial community (McKnight et al. 2019). Alpha diversity statistics were calculated using the Observed, Chao1, Shannon, and Simpson measurements. The alpha diversity measurements were ranked using the Kruskal–Wallis H nonparametric test to determine statistically significant differences between the groups. *P*-values were adjusted using the ‘fdr’ script in R (Benjamini and Hochberg 1995). Beta diversity statistics were calculated using the Bray**–**Curtis dissimilarity measurement. Using the Bray–Curtis dissimilarity measurements, nonmetric multidimensional scaling (NMDS) graphs were created, organized by location and treatment. Alpha and beta diversity statistics were calculated using R-studio (version 2024.12.1 + 402) (Liu et al. 2021a). Model-based linear discriminant analysis (LDA) effect size (LEfSe) was employed to determine the biomarkers likely to account for the differences between groups (Segata et al. 2011). LEfSe analyses were combined with the random forest method, using the Mean Decrease Gini coefficient as indicator value (Beck and Foster 2014). Only the top 20 OTU genera with the highest Mean Decrease Gini values, with *P*-value < .001, were selected.

Microbial co-occurrence networks were generated utilizing the network package SpiecEasi on R, utilizing the SpiecEasy mb method (Friedman and Alm 2012, Kurtz et al. 2015), a correlation threshold of 0.0001, Spearman's correlation coefficient, and the NetCoMi normalization package (Peschel et al. 2021). Correlation maps were created utilizing Gephi (version 0.10.1 202 301 172 018) (Bastian et al. 2009). Further, Gephi was utilized to determine average degree, average weighted degree, and the graph density of the nodes in the network. Betweenness and closeness centralities were determined using the network diameter functionality in Gephi, utilizing the algorithms developed by Brandes (2001). Lastly, average clustering coefficients of nodes were calculated utilizing Latapy (2008) computations on Gephi.

### Microbial functional analyses

OTUs were assigned to functional groups utilizing the FAPROTAX package (Louca et al. 2016) for bacteria and the FungalTraits package (Põlme et al. 2020) for fungi. Once functional groups were determined, statistically significant differences between treatments were determined using LEFse and analysis of variance (ANOVA) analyses. Environmental data was correlated with the functional groups, utilizing the Spearman's rank correlation coefficient, to determine the directional correlation between soil chemical and enzymatic data and functional groups.

### qPCR analyses on Langgewens

qPCR analyses were done on the Langgewens samples to determine the gene-copy number of nitrogen cycle-associated genes present in the microbiome of wheat. This study primarily focused on genes associated with nitrification (ammonia to nitrite)—*AmoA* (Rotthauwe et al. 1997), nitrification (nitrite-to-nitrate)—*nxrB*, denitrification (nitrate to nitrite) (Pester et al. 2014)—*NapA* and *NarG* (Smith et al., 2007), denitrification (nitrite to nitric oxide)—*NirK* and *NirS* (Braker et al. 1998), and nitrogen fixation (N_2_ to ammonia)—*nifH* (Boulygina et al. 2002). Primer sequences and annealing temperatures can be found in Table 1. qPCRs were performed utilizing the Luna^®^ Universal qPCR Master Mix (New England Biolabs, MA) according to the manufacturer's instructions, using 0.25 µM of each primer and 100 ng of DNA template. Reactions were denatured at 95°C for 10 min, followed by 45 cycles of denaturation at 95°C for 10 s, annealing at temperature according to Table 1 for 30 s, and extension at 72°C for 30 s, followed by a final melt-curve stage of 95°C for 30 s, another annealing step according to temperatures in Table 1 for 1 min, followed by a melt-curve analysis increasing in increments of +0.3°C till reaching 95°C for a final 30 s. Experiments were conducted on a StepOnePlus™ Real-Time PCR System (Applied Biosystems, MA) at the CAF (Stellenbosch, South Africa). Data were collected using the StepOne™Software version 2.3.

**Table 1.** Nitrogen cycle associated genes, qPCR primer sequences, expected amplicon sizes, and annealing temperatures used in this study.

Targeted gene	Forward primer	Forward primer sequence	Reverse primer	Reverse primer sequence	Expected amplicon size (bp)	Annealing temp
*AmoA*	amoA-1F	GGGG TTTCTACTGGTGGT	amoA-2R	CCCCTCKGSAAAGCCTTCTTC	∼490	52°C
*NapA*	napA-1F	GTY ATG GAR GAA AAA TTC AA	napA-1R	GAR CCG AAC ATG CCR AC	∼120	55°C
*NarG*	narG-1F	GAC TTC CGC ATG TCR AC	narG-1R	TTY TCG TAC CAG GTG GC	∼100	56°C
*NirK*	nirK1F	GGM ATG GTK CCS TGG CA	nirK5R	GCC TCG ATC AGR TTR TGG	∼500	51°C
*NirS*	nirS1F	CCT AYT GGC CGC CRC ART	nirS3R	GCC GCC GTC RTG VAG GAA	∼350	51°C
*nxrB*	nxrB169F	TACATGTGGTGGAACA	nxrB638R	CGGTTCTGGTCRATCA	∼470	56°C
*nifH*	nifH F1	TAY GGN AAR GGN GGN ATY GGN AAR TC	nifH 439R	GGC ATN GCR AAN CCD CCR CA	400	60°C

## Results

### Relative abundance and statistics

Amplicon sequences were rarified to 13 170 and 9857,16S and ITS OTUs, respectively. Fungal OTUs were assigned to 16 phyla, 58 classes, 127 orders, 336 families, and 737 genera, whereas bacterial OTUs were assigned to 27 phyla, 77 classes, 197 orders, 390 families, and 1098 genera (Fig. S2). Genus-level classification of OTUs resulted in a greater localization of species to specific farms. Bacteria were more evenly distributed across various groups, with 12 of the top 40 genera ubiquitously distributed across all samples.

### Diversity-based analyses

Alpha diversity analyses (Table 2A) revealed significant differences (*P* < .05) between the PP fungal communities on Langgewens and significant differences (*P* < .05) between the PP bacterial communities on Tygerhoek. Furthermore, bacterial communities on Langgewens and fungal communities on Tygerhoek appeared to be more evenly distributed and uniform during the PP timepoint. However, no significant differences were observed when the alpha diversity analyses between farms were assessed for the PP period. Additionally, no significant differences between treatments were observed when samples within the WGP were assessed, although the bacterial communities on Langgewens tended to get more diverse. However, the opposite phenomenon was observed with the fungal communities on Langgewens and the bacterial and fungal communities on Tygerhoek, with adjusted *P*-values trending towards 1, suggesting uniformity between the communities. This is further supported by the significant differences (*P* < .01) in Observed and Chao1 diversity indexes when comparing fungal communities between farms for the WGP. Lastly, no significant difference could be observed between treatments for the AH timepoint, although all groups tended to get more diverse, with adjusted *P*-values trending downward. No significant differences could be observed for bacterial communities between farms. However, significant differences in the Observed (*P* < .05) and Chao1 (*P* < .01) indexes could be observed between fungal communities on the different farms.

**Table 2.** Alpha and Beta diversity statistics for wheat farms and treatments, for agricultural season 2019.

(A) Alpha diversity statistics													
Farm	Comparison	Measure	Test method	PP			WGP			AH (post-senescence)			
				P.unadjusted	P.adjusted	Sig	P.unadjusted	P.adjusted	Sig	P.unadjusted	P.adjusted	Sig	
Langgewens	Bacterial communities	Observed	Kruskal–Wallis Rank Sum	0.4 842 538	0.8 716 568	ns	0.26 790 971	0.3 444 553	ns	0.9 390 088	0.9 598 909	ns	
		Chao1		0.2 811 109	0.8 716 568	ns	0.55 092 651	0.5 509 265	ns	0.9 598 909	0.9 598 909	ns	
		Shannon		0.8 844 343	0.9 487 295	ns	0.04 351 649	0.1 493 612	ns	0.9 267 945	0.9 598 909	ns	
		Simpson		0.9 487 295	0.9 487 295	ns	0.04 978 707	0.1 493 612	ns	0.7 776 630	0.9 598 909	ns	
	Fungal communities	Observed		**0.001 256 522**	**0.0 056 543**	**	0.5 004 199	0.9 392 071	ns	0.02 975 911	0.07 615 118	ns	
		Chao1		**0.006 064 743**	**0.0 181 942**	*	0.6 183 076	0.9 392 071	ns	0.02 975 911	0.07 615 118	ns	
		Shannon		**0.019 532 559**	**0.0 292 988**	*	0.7 939 227	0.9 392 071	ns	0.45 942 582	0.68 913 874	ns	
		Simpson		**0.031 922 492**	**0.0 319 225**	*	0.8 740 520	0.9 392 071	ns	0.93 223 026	0.93 223 026	ns	
Tygerhoek	Bacterial communities	Observed		0.193 123 272	0.2 604 936	ns	0.7 351 415	0.7 351 415	ns	0.4 221 820	0.6 268 724	ns	
		Chao1		0.277 842 255	0.3 125 725	ns	0.2 188 802	0.5 845 911	ns	0.5 572 199	0.6 268 724	ns	
		Shannon		**0.004 422 489**	**0.0 199 012**	*	0.5 836 455	0.7 351 415	ns	0.2 620 601	0.5 896 353	ns	
		Simpson		0.050 372 792	0.1 133 388	ns	0.3 897 274	0.5 845 911	ns	0.1 426 490	0.4 279 469	ns	
	Fungal communities	Observed		0.5 225 039	0.7 837 559	ns	0.6 677 468	1.0 000 000	ns	0.1 907 712	0.2 577 149	ns	
		Chao1		0.7 776 630	0.8 054 338	ns	0.6 939 297	1.0 000 000	ns	0.2 026 062	0.2 577 149	ns	
		Shannon		0.2 290 799	0.7 837 559	ns	1.0 000 000	1.0 000 000	ns	0.2 237 835	0.2 577 149	ns	
		Simpson		0.4 594 258	0.7 837 559	ns	0.9 259 611	1.0 000 000	ns	0.0 964 065	0.2 577 149	ns	
Between farms	Bacterial communities	Observed		0.33 433 836	0.3 761 307	ns	0.0 565 316	0.2 543 923	ns	0.1 637 570	0.2 947 625	ns	
		Chao1		0.08 183 742	0.3 198 631	ns	0.2 040 239	0.3 671 420	ns	0.0 619 470	0.2 352 689	ns	
		Shannon		0.46 680 472	0.4 668 047	ns	0.1 190 329	0.3 570 987	ns	0.92 438 171	0.9 243 817	ns	
		Simpson		0.26 814 220	0.3 761 307	ns	0.2 986 976	0.3 671 420	ns	0.8 494 410	0.9 243 817	ns	
	Fungal communities	Observed		0.88 676 312	0.8 867 631	ns	**0.0 009 936**	**0.0 017 976**	**	**0.0 056 213**	**0.0 101 183**	*	
		Chao1		0.09 992 709	0.3 632 148	ns	**0.0 009 987**	**0.0 017 976**	**	**0.0 019 314**	**0.0 057 942**	**	
		Shannon		0.87 430 472	0.8 867 631	ns	0.0 433 081	0.0 649 622	ns	0.8 247 263	0.8 247 263	ns	
		Simpson		0.59 067 633	0.8 860 145	ns	0.0 734 883	0.0 734 884	ns	0.4 289 644	0.4 825 850	ns	

(B) Beta diversity statistics													
Farm	Kingdom	Comparison	Measure	Test method	PP			WGP			AH (post-senescence)		
					Observed *P*-value	Permutation *P*-value	Sig,	Observed *P*-value	Permutation *P*-value	Sig	Observed *P*-value	Permutation *P*-value	Sig
Langgewens	Bacterial communities	Total Treatment	Bray–Curtis's dissimilarity	Multivariate analysis of variance	.051		ns	.333		ns	**.019**		*
		WW vs WC			.324	.336	ns	.252	.456	ns	.033	.099	ns
		WW vs WM			.336	.336	ns	.588	.588	ns	.178	.178	ns
		WM vs WC			**.003**	**.009**	**	.304	.456	ns	.114	.171	ns
	Fungal communities	Total Treatment			**.001**		**	.058		ns	**.001**		***
		WW vs WC			**.005**	**.006**	**	.031	.0930	ns	**.002**	**.006**	**
		WW vs WM			**.004**	**.006**	**	.261	.2610	ns	**.019**	**.019**	*
		WM vs WC			**.006**	**.006**	**	.111	.1665	ns	**.005**	**.008**	**
Tygerhoek	Bacterial communities	Total Treatment			**.001**		***	.273		ns	**.006**		**
		WW vs WC			**.004**	**.006**	**	.216	.324	ns	**.008**	**.024**	*
		WW vs WM			**.002**	**.006**	**	.144	.324	ns	.107	.107	ns
		WM vs WC			.183	.183	ns	.615	.615	ns	.069	.104	ns
	Fungal communities	Total Treatment			**.002**		**	**.003**		**	**.001**		***
		WW vs WC			**.019**	**.019**	*	.033	.054	ns	**.003**	**.005**	**
		WW vs WM			**.003**	**.006**	**	.119	.119	ns	**.038**	**.038**	*
		WM vs WC			**.004**	**.006**	**	.036	.054	ns	**.003**	**.005**	**

Key: ‘ns’ non significance, *P < .05, **P < .01, ***P < .001.

Beta diversity analyses (Table 2B) followed a similar trend as the alpha diversity analyses, with PP and AH samples revealing significant differences between treatments, whereas the WGP trended towards uniformity. Significant differences (*P* < .01) could be observed between PP bacterial communities on Langgewens when WM and WC treatments were assessed. Moreover, significant differences (*P* < .01) in beta diversity could be observed for PP fungal communities on Langgewens and Tygerhoek between all treatments, with the only exception being PP fungal communities between WW and WC (*P* < .05). Additionally, PP bacterial communities on Tygerhoek were significantly different (*P* < .01) between treatments, except between WM and WC, where no significant differences were observed. No significant beta diversity differences were observed between treatments for the WGP. Lastly, when beta-diversity for the AH timepoint was assessed, significant differences (bacteria *P* < .05; fungi *P* < .001) were observed between treatment groups on both farms. However, when individual treatments were compared AH, fungal communities remained significantly different, with the most significant differences (*P* < .01) being between WW and WC communities and WC and WM treatments. Only WW and WC showed significant differences (*P* < .05) on Tygerhoek for bacterial communities at this timepoint.

**Figure 1.**
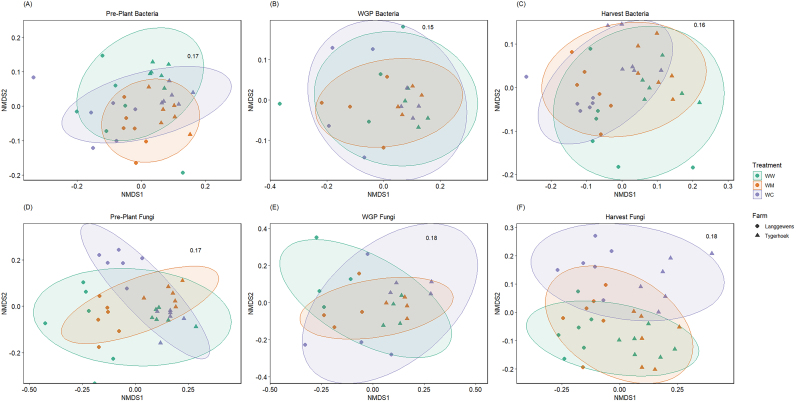
NMDS plots of the beta diversity differences between treatments, across three time-points (A) PP bacteria, (B) WGP bacteria, (C) AH bacteria, (D) PP fungi, (E) WGP fungi, and (F) AH fungi, for wheat agricultural season 2019. Key: Shapes representing farms—polygons for Langgewens and triangles for Tygerhoek, different colours represent the different treatments..

NMDS plots using the beta diversity values of each sample revealed samples clustered by farm (Fig. 1), with Tygerhoek (▴) samples clustering more tightly together than those of Langgewens (⬤). At PP, samples were grouped by treatment on each farm, with bacterial samples from Langgewens showing the most overlap between treatments. PP fungal communities showed the most distinct clustering within their distinct treatments and farms. During the WGP, samples showed the most overlap between treatments than at any of the other sampling time points. Samples clustered into their distinctive treatment groups during the AH sampling time-point, with WC and WW samples clustering the furthest apart. Lastly, WC fungal samples clustered the furthest from the other treatments AH, reflecting what was seen in the PP fungal samples of Langgewens.

### LEfSe OTU analyses

**Figure 2.**
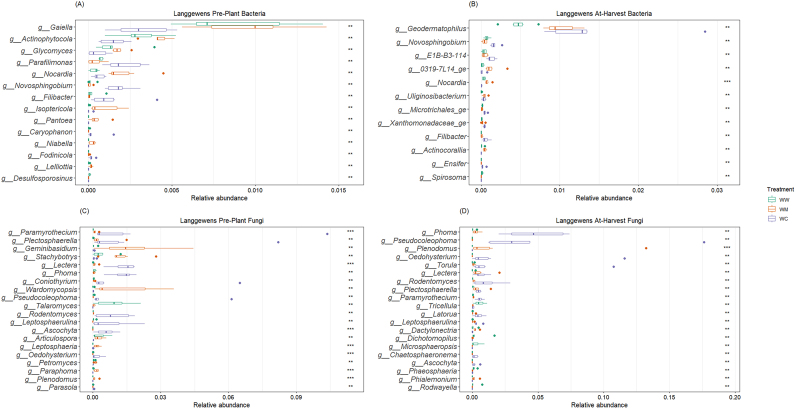
LEfSe comparison of the differences attributed to treatments on Langgewens—for wheat agricultural season 2019, (A) PP bacteria; (B) AH bacteria, (C) PP fungi, and (D) AH fungi, by relative abundance (%) Key: Treatments: WW, WC, and WM.

**Figure 3.**
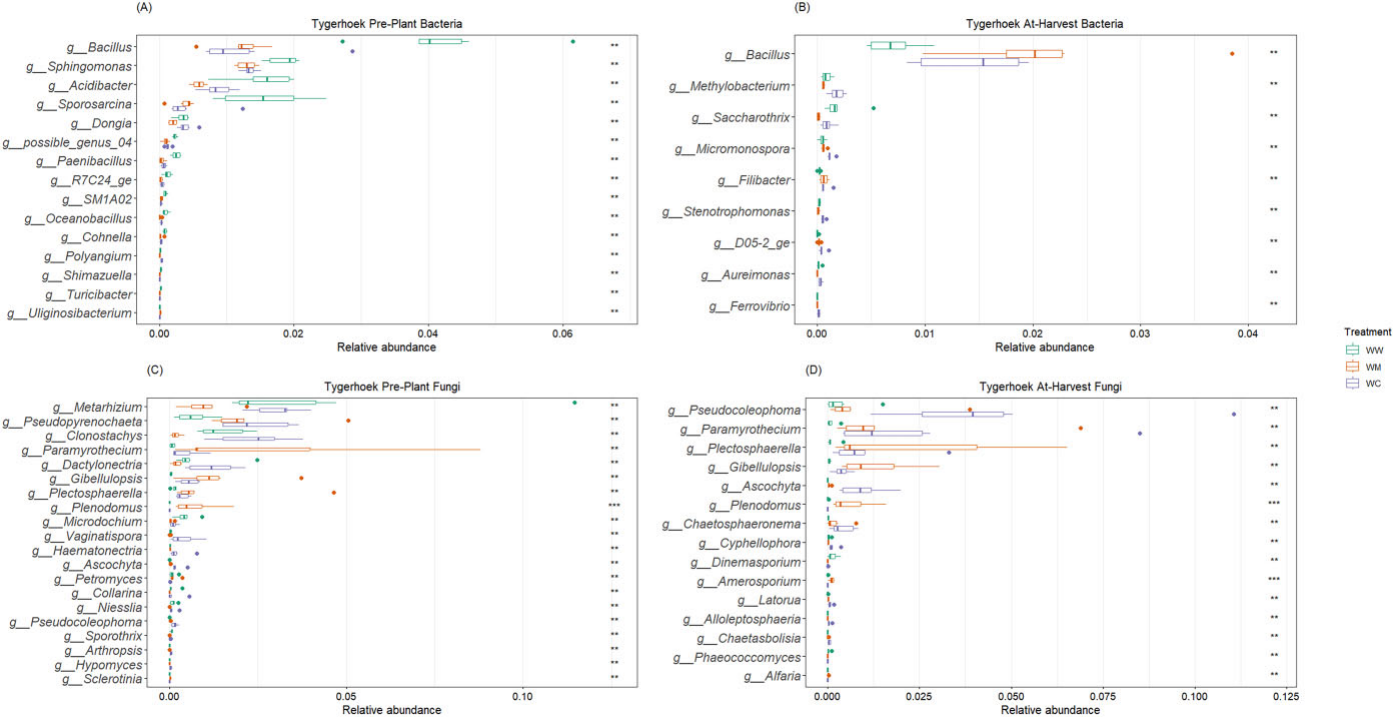
LEfSe comparison of the differences attributed to treatments on Tygerhoek—for wheat agricultural season 2019, (A) PP bacteria; (B) AH bacteria, (C) PP fungi, and (D) AH fungi, by relative abundance (%) Key: Treatments: WW, WC, and WM.

LEfSe analysis did not show any specific genera that explained the difference between treatments during the WGP. However, several genera were identified that explained the differences between treatments at the PP and AH time points. These genera tended to be farm-specific, and subsequently, farms were represented in different graphs (Figs. 2 and 3). Contrary to this, certain fungi were found to be significantly different between treatments at PP on Langgewens and AH. These included *Paramyrothecium, Plectosphaerellia, Lectera, Phoma*, and *Pseudocoleophoma*, which were all associated with WC treatments, where they were more abundant (*P* < .01). At Tygerhoek, only *Bacillus* was significantly associated with treatments in both PP and AH soils. However, at PP, *Bacillus* was significantly (*P* < .01) associated with WW soils, whereas AH, it was significantly associated with WM soils, suggesting a selection pressure other than crop rotation. Fungal communities showed similar patterns on both farms. While no significant genera could be identified to explain the differences between treatments during the WGP, certain genera were significantly associated with crop rotations when looking at the PP and AH time points, including *Paramyrothecium, Gibellulopsis, Plectosphaerellia, Plenodomus*, and *Ascochyta*. All of these tended to correlate with the WC and WM treatments at PP and AH (*P* < .01). It is important to note here that LEfSe analyses have certain limitations and results should be interpreted with caution, as LEfSe analyses are particularly sensitive to group size and taxonomic imbalances, particularly present in large environmental datasets, such as in this study. Additionally, LEfSe analyses do not account for correlated or phylogenetically related taxa.

**Figure 4:**
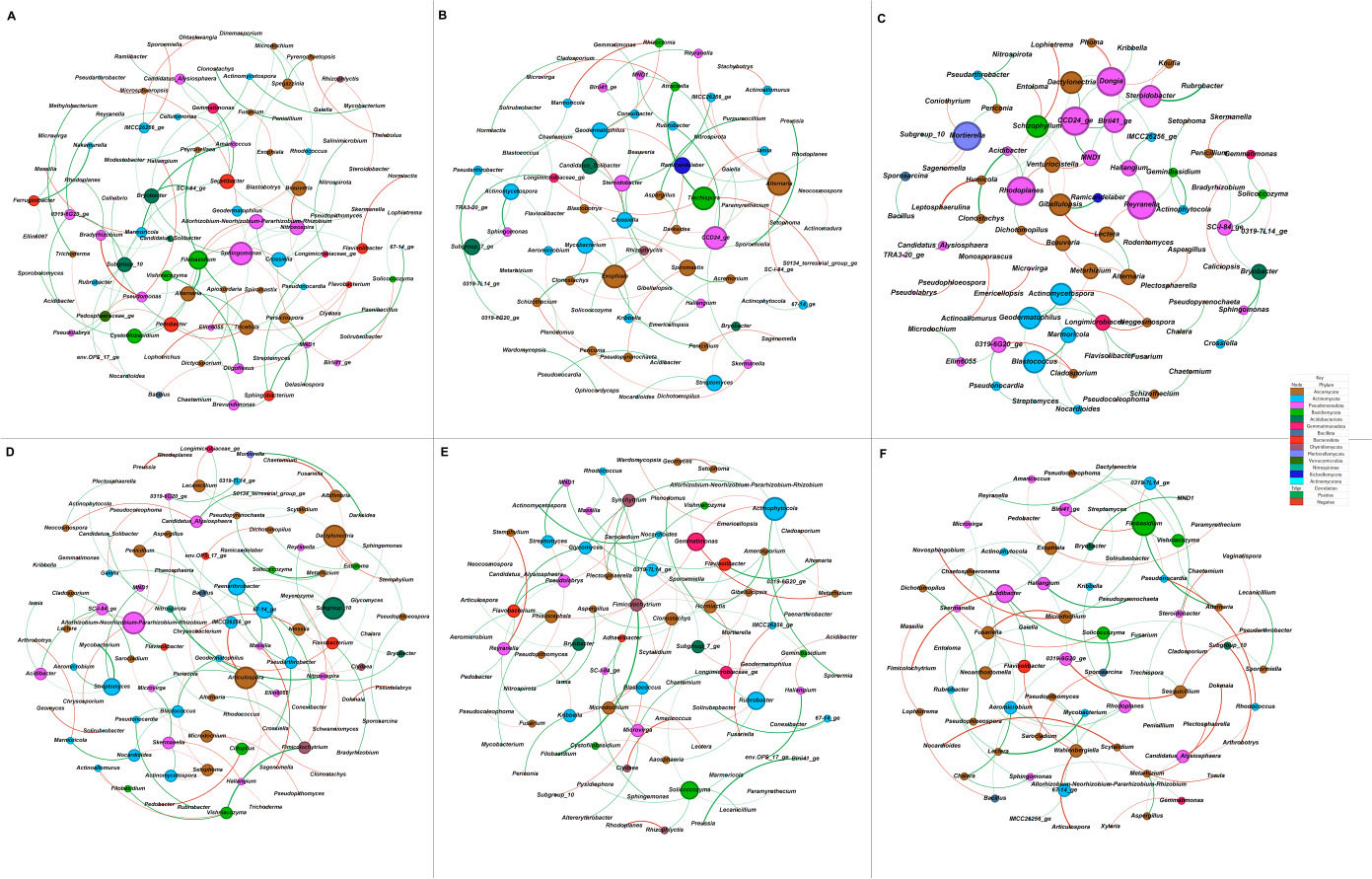
Co-occurrence network analyses of the core wheat microbiome by treatment—(A) Langgewens WW, (B) Langgewens WM, (C) Langgewens WC, (D) Tygerhoek WW, (E) Tygerhoek WM, and (F) Tygerhoek WC. Nodes indicated by circles, edges indicated by lines—green lines indicate positive correlations and red lines indicate negative correlations.

### Co-occurrence network analyses

Farm-wide co-occurrence analyses (Figs. S3, 4, and Table S1) at the PP time point revealed that key network nodes were dominated by phyla *Ascomycota* (37.8–45.05%), Actinomycetota (18.07%–28.05%) and Pseudomonadota (16.48%–22.89%). *Ascomycota* were most prevalent in WW PP soils and least prevalent in WM PP soils. In contrast, Actinomycetota were least prevalent in WW PP soils and most prevalent in WM PP soils. Pseudomonadota were most prevalent in WC PP soils and least prevalent in WW soils. The core nodes of the PP WW soil network consisted of genera *Pseudopyrenochaeta, Exophiala, Metarhizium, Trichoderma*, and *Schizothecium* (*Ascomycota*), *Bacillus* (Bacillota), *Mycobacterium* and *Solirubrobacter* (Actinomycetota), and *Bryobacter* (Acidobacteriota). The core nodes of the PP WM soil network consisted of *Trechispora* (*Basidiomycota*), *Alternaria* and *Exophiala* (*Ascomycota*), *CCD24* and *Steroidobacter* (Pseudomonadota), *Streptomyces, Mycobacterium, Actinomycetospora*, and *Geodermatophilus* (Actinomycetota), and *Candidatus* Solibacter (Acidobacteriota). Lastly, the core nodes of the PP WC soil network consisted of *Dactylonectria, Gibellulopsis*, and *Metarhizium* (*Ascomycota*), *Mortierella* (*Mortierellomycota*), *Schizophyllum* (*Basidiomycota*), *Rhodoplanes, CCD24, Reyranella, Dongia, BIrii41* and *Steroidobacter* (Pseudomonadota), *Blastococcus, Actinomycetospora, Geodermatophilus* (Actinomycetota), and *Bryobacter* (Acidobacteriota).

When co-occurrence networks were analysed through the perspective of farms and treatments during the WGP, shifts in key network nodes were evident. Langgewens soil networks were dominated by phyla Pseudomonadota (20.51%–27.84%), *Ascomycota* (27.84%–37.78%), and Actinomycetota (18.56%–21.79%). On Langgewens WW soils, nodes were co-dominated by phyla Pseudomonadota (27.84%) and *Ascomycota* (27.84%). *Ascomycota* was dominant in WM and WC soils (37.78% and 33.33%, respectively). Langgewens WW soils (Table S1), had many centralized nodes consisting of genera *Filobasidium, Cystofilobasidium*, and *Vishniacozyma* (*Basidiomycota*), *Alternaria, Tricellula*, and *Beauveria* (*Ascomycota*), *Sphingomonas, Nitrosospira*, and *Allorhizobium*-*Neorhizobium*-*Pararhizobium*-*Rhizobium* (Pseudomonadota), and *Bryobacter* and *Subgroup_10* (Acidobacteriota). Langgewens WM centralized nodes consisted of *Dokmaia, Pseudopyrenochaeta, Periconia, Coniochaeta, Caliciopsis*, and *Alternaria* (*Ascomycota*), *Vishniacozyma* (*Basidiomycota*), *Aeromicrobium, Nocardioides*, and *Gaiella* (Actinomycetota), *Flavobacterium, Sphingobacterium*, and *Ohtaekwangia* (Bacteroidota), and *Gemmatimonas* (Gemmatimonadota), Whereas Langgewens WC soils consisted of centralized nodes *Phoma, Alternaria, Rodentomyces*, and *Coniothyrium* (*Ascomycota*), *Subgroup_10* and *Bryobacter* (Acidobacteriota), *Cellvibrio, Microvirga*, and *Devosia* (Pseudomonadota), and *Solirubrobacter* (Actinomycetota). Lastly, Tygerhoek soils were dominated by phyla *Ascomycota* (37.5–44.3%), Actinomycetota (20.25–24.04%), and Pseudomonadota (16.35–18.99%). Tygerhoek WW centralized nodes consisted of genera *Articulospora, Dactylonectria, Penicillium, Microdochium, Setophoma, Albifimbria*, and *Lecanicillium* (*Ascomycota*), *Vishniacozyma* (*Basidiomycota*), *Subgroup_10* (Acidobacteriota), *Allorhizobium*-*Neorhizobium*-*Pararhizobium*-*Rhizobium, Solirubrobacterales bacterium 67–14, Streptomyces, Paenarthrobacter, Nocardioides, Pseudarthrobacter, Blastococcus*, and *Actinomycetospora* (Actinomycetota), and *Flavobacterium* (Bacteroidota). Tygerhoek WM centralized nodes consisted of genera *Solicoccozyma* (*Basidiomycota*), *Synchytrium* (*Chytridiomycota*), *Actinophytocola, Rubrobacter, Streptomyces, Blastococcus*, uncultured bacterium *0319–7L14, Kribbella*, and *Glycomyces* (Actinomycetota), *Gemmatimonas* and *Longimicrobiaceae_unclassified_ge* (Gemmatimonadota), *Fimicolochytrium* and *Microvirga, Reyranella, Pseudolabrys* (Pseudomonadota), *Streptomyces, Blastococcus, Kribbella, Glycomyces*, (Actinomycetota), and *Bryobacter* and *Subgroup_7* (Acidobacteriota). And Tygerhoek WC centralized nodes consisted of genera *Sesquicillium, Fusariella, Microdochium, Neoanthostomella, Wahlenbergiella*, and *Exophiala* (*Ascomycota*), *Filobasidium, Solicoccozyma*, and *Vishniacozyma* (*Basidiomycota*), *Acidibacter, BIrii41, Rhodoplanes, Haliangium, Candidatus* Alysiosphaera, and uncultured bacterium *0319–6G20* (Pseudomonadota), *Solirubrobacterales bacterium 67–14, Aeromicrobium*, and uncultured bacterium *0319–7L14* (Actinomycetota).

### Functional analyses

**Figure 5.**
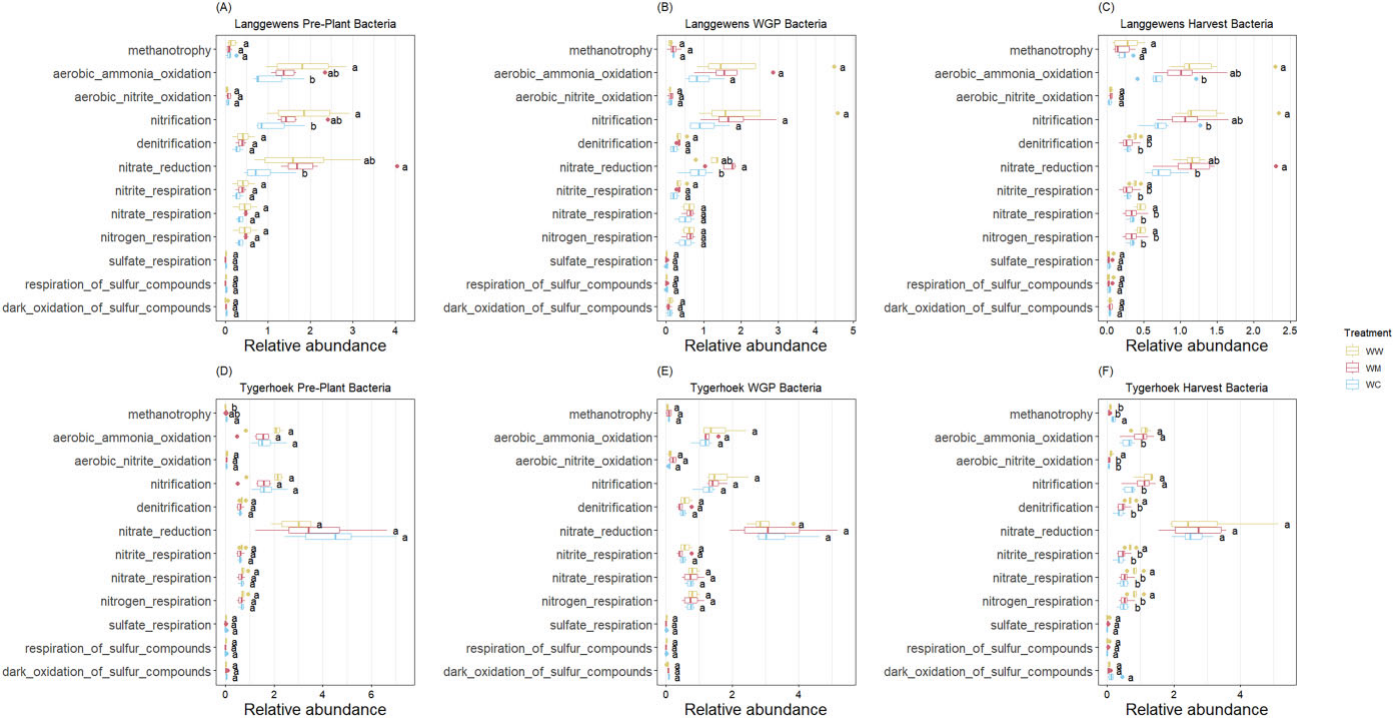
LDA utilizing an ANOVA comparison of the different bacterial functional groups between treatments and time-points for wheat agricultural season 2019, by relative abundance (%). (A) Langgewens PP; (B) Langgewens WGP, (C) Langgewens AH, (D) Tygerhoek PP, (E) Tygerhoek WGP, and (F) Tygerhoek AH. Key: Treatments: WW, WC, and WM.

**Figure 6.**
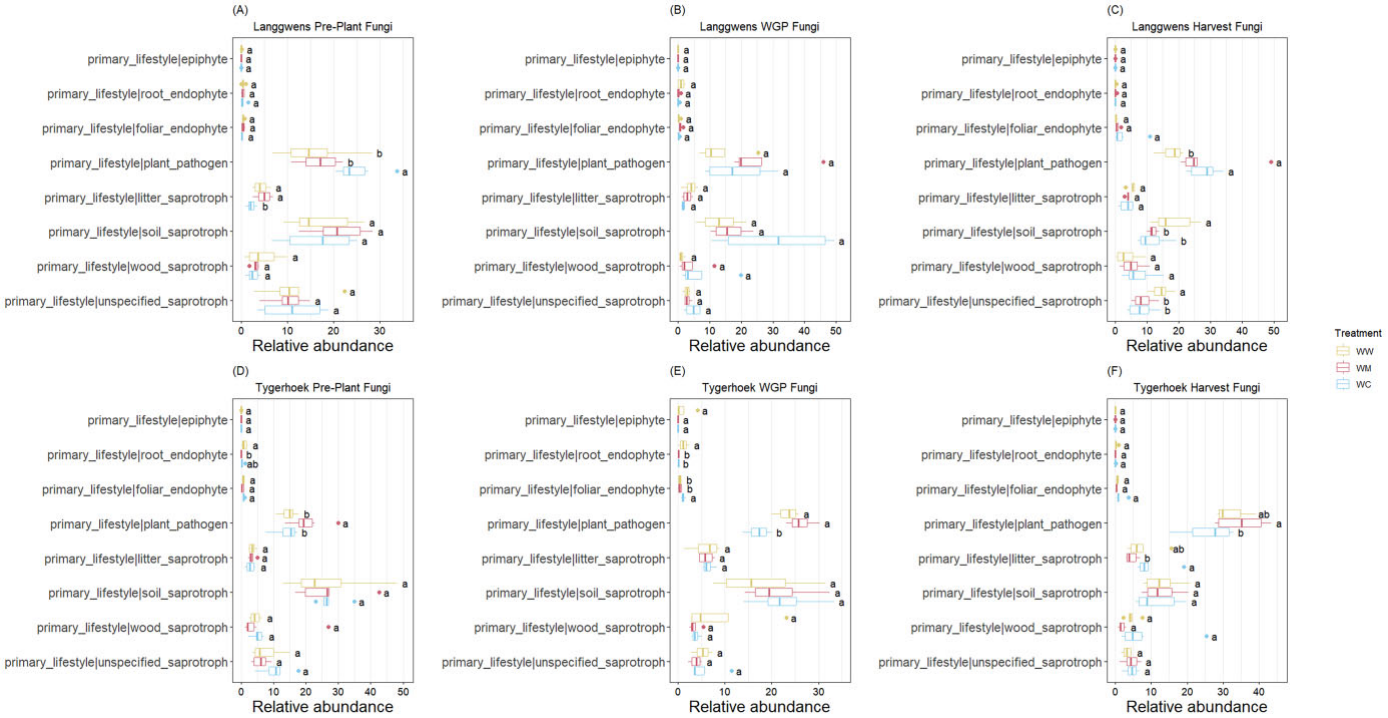
LDA utilizing an ANOVA comparison of the different fungal functional groups between treatments and time-points for wheat agricultural season 2019, by relative abundance (%). (A) Langgewens PP; (B) Langgewens WGP, (C) Langgewens AH, (D) Tygerhoek PP, (E) Tygerhoek WGP, and (F) Tygerhoek AH. Key: Treatments: WW, WC, and WM.

Several trends were evident when assessing microbial functional groups between crop-rotation treatments over time (Figs. 5 and 6). Methanotrophic communities showed significant differences between PP and AH time points on Tygerhoek, where methanotrophs were most abundant in WC soils. Functional groups associated with the nitrogen cycle, such as bacteria associated with aerobic ammonia oxidation, were most abundant in WW and WM soils and least abundant in WC soils across both farms. Functional groups associated with nitrification revealed significant differences between treatments on Langgewens during the PP and AH time-point, with WW and WM soils having similar relative abundances. WC soils saw the greatest drop in the relative abundance of functional groups associated with nitrification over time. Denitrification, nitrite-, nitrate-, and nitrogen respiration followed the same pattern on both farms, with significant differences in relative abundance only appearing AH, with WW soils having significantly more relative abundance in these groups than WC and WM soils, which had similar abundances AH. Functional groups associated with nitrate reduction revealed significant differences between treatments across all time points on Langgewens but no significant differences between treatments on Tygerhoek. On Langgewens nitrate reduction groups were most abundant in WM soils, followed by WW soils and least abundant in WC soils. However, on Tygerhoek, nitrate reduction groups were most abundant in WC soils at the start of the season and least abundant by the end of the season.

Fungal functional groups were classified into different lifestyles; for the purposes of this study, we only assessed the primary lifestyle of these functional groups. Fungi associated with the functional group epiphytes were only significantly different on Langgewens at the AH time point. This was due to the significant decrease in the abundance of epiphytes in WC soils AH. Root endophytes were more abundant in Tygerhoek WW soils than in WM and WC soils.

On Tygerhoek, foliar endophytes were significantly enriched during the WGP and AH time-points for WW and WM but not for WC soils. Functional groups associated with plant pathogenicity revealed significant differences between treatments. On Langgewens, WC soils were significantly more abundant in plant pathogen groups at PP. However, all treatments had similar abundances during the WGP, and by the AH time point, WW soils had significantly lower relative abundance in plant pathogens than WM and WC soils. On Tygerhoek, WM soils were the most abundant in plant pathogens. During the WGP, WW and WM soils had similar relative abundances in plant pathogen groups, and WC soils had significantly less. These ratios were stable up to the AH time-point, with the relative abundance of pathogens dropping slightly in WW soils compared to WM soils. On the other hand, WC soils on Langgewens and WM soils on Tygerhoek had significantly higher relative abundance in litter saprotrophs during the PP time-point. No significant differences were observed during the WGP on either farm. AH, a significant decrease in the relative abundance of litter saprotrophs was observed in WC soils on Tygerhoek only.

### Environmental correlations and qPCR analyses

Environmental correlation analyses between soil geochemical measurements and qPCR analyses with microbial functional groups revealed no significant correlations during the WGP. Significant correlations, however, were observed between these measurements and individual families, and mostly just during the PP bulk soil time point. Elemental analyses (Fig. S4) revealed that microbial families showed the greatest level of correlation (both positive and negative) with hydrogen ions (H) and soluble sulphur (Soluble S). Bacterial families *Xanthobacteraceae, Steroidobacteraceae, Reyranellaceae, Haliangiaceae, Entothenellaceae, Dongiaceae, Diplorickettsiaceae*, and *Blrii41* were all negatively correlated with these elements. On the other hand, bacterial families *Spingomonadaceae*, an unclassified *Sericytochromatia* family, *Nakamurellaceae, Microbacteriaceae, Burkholderiaceae*, and *Alicyclobacillaceae* were all positively correlated with these elements. Alternatively, fungal families that were negatively correlated with these elements were *Pseudeurotiaceae, Morosphaeriaceae, Kickxellaceae, Entolomataceae, Clavicipitaceae*, and *Chatomiaceae*. Those positively correlated with these elements included *Sordariaceae, Herpotichiellaceae, Coryneliaceae*, and *Chatothyriaceae*. When soil enzyme analyses were correlated to microbial families, a more diverse correlation pattern could be observed (Fig. S5). For bacterial families, pH, soil moisture, soil electroconductivity, beta glucosidase activity, available phosphorus, and nitrate availability were the greatest correlating factors. For fungal families, a more diverse correlation pattern was observed.

**Figure 7.**
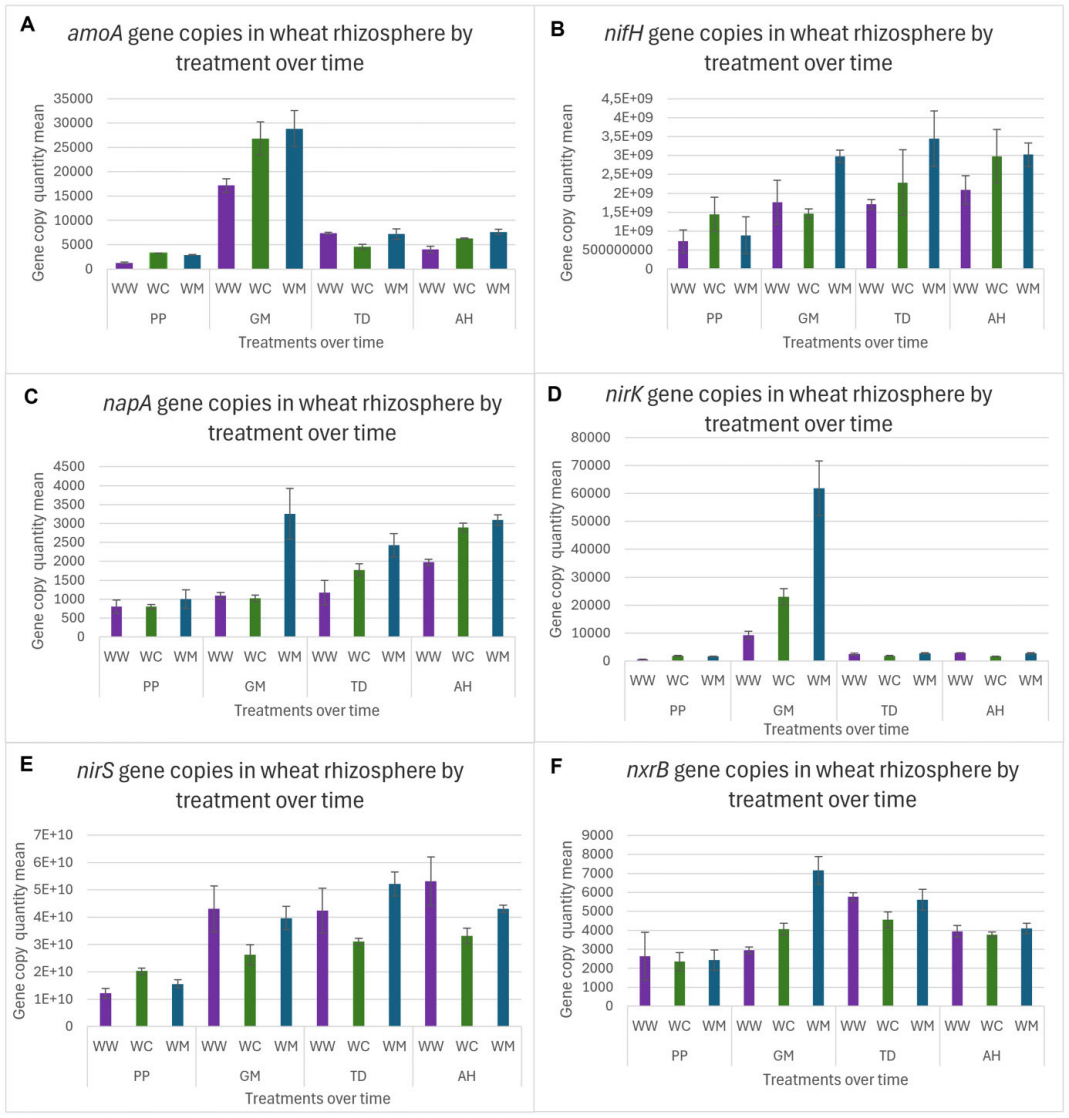
qPCR gene copy counts for nitrogen-cycling associated genes between treatments and across time-points for Langgewens, wheat agricultural season 2019. (A) Nitrification associated gene *AmoA*, (B) nitrogen fixation associated gene *nifH*, (C) denitrification associated gene *napA*, (D) denitrification associated gene *nirK*, (E) denitrification associated gene *nirS*, and (F) nitrification associated gene *nxrB*. Key: WW, WC, WM, PP, GM, TD (GM + TD = WGP), and AH.

qPCR analyses (Fig. 7) on Langgewens revealed a significant trend where copies of genes encoding *amoA, nirS, nirK*, and *nxrB* all increased during the GM period. However, *amoA* and *nirK* decreased in subsequent sampling time points, *nirS* and *nxrB* genes copies plateaued. Alternatively, gene copies encoding for *nifH* and *napA* increased throughout the season, peaking AH. Gene copies for all genes examined were most abundant in WM soils, becoming evident during the GM time-point, even though these soils had fewer or equal starting points at PP. WW soils, on the other hand, were less affected by changes throughout the season when compared to the other treatments. The increase in gene copies was not directly correlated with the relative abundance of nitrifying functional groups identified (Fig. 5) across all treatments, however an increase in gene copies was associated with an increase in nitrifying functional groups in WM soils during the WGP.

## Discussion

### Crop rotations

In this study, we investigated the effects of crop rotation systems on the rhizosphere microbiome of wheat in regenerative agricultural settings. The results showed that crop rotations only influenced the PP bulk soils; however, once wheat was introduced to the soil, a significant selective pressure was exerted over the microbiome in the rhizosphere. Significant differences in the alpha-diversity of fungal communities between treatments were observed on Langgewens but not on Tygerhoek. Alpha-diversity between treatments for bacterial communities on Tygerhoek was, however, significant. This implies that crop rotation affected the fungal communities on Langgewens but not at Tygerhoek, which suggests that crop rotation is just one variable that influences the structure of microbial communities. This was not totally unexpected, as previous studies found that the differences attributable to crop rotation may not necessarily be observed in the alpha diversity statistics but in the rare and niche species that are selected by different types of crops (Town et al. 2023, Fritze et al. 2024).

When comparing the beta diversity of fungal communities all were significantly different between treatments. The outlier treatment would reflect this pattern for multiple genera, i.e. WC on Langgewens or WW on Tygerhoek, suggesting that these differences are less due to the specific crop rotation treatments and more due to the location the samples were taken from. Conversely, fungal communities displayed a greater level of heterogeneity across treatments, with certain genera significantly enriched in one treatment and in low abundance in comparative treatments. For example, on Langgewens, *Paramyrothecium*, a potentially opportunistic plant pathogen and saprobe, found in soils (Aumentado et al. 2024), was found to be significantly enriched in WC soils pre-planting, and virtually depleted in the other treatments. On Tygerhoek, however, *Paramyrothecium* was significantly enriched in WM soils while being much less abundant in WC soils. On both farms, *Paramyrothecium* was depleted in WW soils, with abundances approaching zero. There is little indication that differences in potentially pathogenic genera or plant growth-promoting microorganisms were exclusively due to crop rotations. However, some of the identified species of potential plant pathogens were associated with pathogenicity of the previous crop, *Paramyrothecium vignicola* for example, has been described as the active agent in causing leaf spot disease on brassicas (Haituk et al. 2024), while *Paramyrothecium foliicola*, also associated with leaf spot disease, has been identified as abundant in medicago soils (Idbella et al. 2024), potentially explaining their enrichment in these treatments and not in the monoculture soils. These contrasting results on each farm may suggest that local soil environments may interact with crop rotational history to affect the fungal microbiome composition.

Analyses of the core microbiome communities revealed unique core communities associated with each treatment. WW soils had the most homogenous core communities at PP, with the greatest number of high-degree nodes, especially fungal nodes of *Persiciospora, Fusicolla, Fusarium, Thelonectria*, and *Naganishia*, suggesting greater reliance on these genera within these soils. On the other hand, WC soils had the most centralized core community, where genera had the highest betweenness centrality (bc). These genera served as connectors between other genera, representing genera commonly found connecting two separate genera, consisting of *Niesslia, Ramicandelaber, Acrocalymma, Cordyceps*, and *Rhizocola*. However, as Ma et al. (2016) suggest, nodes with high bc and lower degrees should not be considered as key-stone species but rather transitional genera. This makes sense as analyses in this study revealed that WC soils tended to have the most divergence from WW and WM soils. This may be due to greater variability in the selection of rhizosphere communities of canola and wheat soils, comparatively. WM soils had the most concentrated core community at PP, with low degree nodes and BCs approaching zero, aside from *Clonostachys*, which had a low but similar BC to WW and WC nodes. This may be attributable to the high degree of similarity between medicago and wheat communities, as seen in the high degree of similarity in the other analyses done in this study. Additionally, while high bc may indicate a central network role, further validation e.g. functional assays are needed to confirm ecological importance and functionality.

Functional analyses of the PP soils (Figs. 5 and 6) revealed greater variability attributable to farms than treatments. However, we should highlight that for bacteria, WW and WM soils revealed the greatest level of similarity in functional groups, whereas WC soils showed the greatest level of variability. For fungal communities, the trend continues, with functional groups associated with plant pathogenicity and those associated with litter saprobes significantly more enriched in WC soils on Langgewens, compared to the other treatments. On Tygerhoek, these groups were more enriched in WM soils. Considering that these analyses throughout this article revealed that WC soils on Langgewens and WM soils on Tygerhoek are consistently different to their treatment counterparts, it is unsurprising to see it reflected in the functional analyses.

### Wheat growth period (WGP)

During the GM and TD time points, grouped as the WGP, the rhizosphere microbiome communities trended towards similarity. This trend was especially noticeable when the alpha diversity of the fungal communities (Table 2) was assessed, where fungal communities on both farms had adjusted p-Values approaching 1. Bacterial alpha diversity statistics were less uniform, with bacteria on Langgewens becoming more diverse while communities on Tygerhoek became less diverse. Analyses of the differences between farms revealed statistically significant differences (*P* < .01) between fungal communities on each farm, suggesting that while fungal communities on each farm appeared to be selected for by the wheat crop, these selected communities were unique to each cultivar and/or a product of the environments they were found in. Furthermore, LEfSe analyses (Figs. 2 and 3) revealed that there were no significant differences between the treatments due to crop rotation, compounding the evidence that during the period where the wheat plant is alive and growing, a distinct community is being selected for across the fields, regardless of the crop planted in the previous season.

Co-occurrence analyses (Figs. S3 and 4) revealed that Subgroup 10 and *Bryobacter* (Acidobacteriota), *Sphingomonas* (Pseudomonadota), P*seudopyrenochaeta, Exophiala*, and *Metarhizium* (*Ascomycota*), *Mycobacterium, Solirubrobacter*, and *Marmoricola* (Actinomycetota), *Acidibacter* and *Skermanella* (Pseudomonadota) formed central nodes in all wheat networks during the WGP. These nodes can be tracked back to PP network nodes. Interestingly, even though *Ascomycota* was the most dominant phylum in all networks, in Langgewens WW soils, Pseudomonadota were co-dominant, which was not observed in any of the other soils. Another interesting observation is that in most soils, the positive correlation network dominated on average 2:1. However, on Langgewens WM and WC soils and a roughly equal split between positive and negatively correlated taxa, on Tygerhoek, this was the case for WW soils. For Langgewens, this may be indicative of a competing microbial network in the WM and WC, potentially due to crop rotations. However, this notion is dispelled when Tygerhoek soils were investigated, where the inverse was true, suggesting that an alternative influence is affecting the correlations. Perhaps a greater difference between field replicate soils on the same farm than previously observed could explain the differences, meaning that it is not competing microbial networks influencing the differences, but two separate communities on each replicate responsible for these differences and are subsequently negatively correlated.

Functional analyses (Figs. 5 and 6) revealed that some significant differences between treatments, like for root endophytes on Tygerhoek, for example, were present at the start of the season and were not introduced alongside wheat. Interestingly, while fungal functional groups converged in functionality on Langgewens, on Tygerhoek, some of the functional groups diversified during the WGP to include foliar endophytes and plant pathogens, with WC communities diverging from those associated with WW and WM treatments. When functional bacterial groups were assessed, communities trended towards uniformity from the PP to WGP time-points, only shifting in significance AH. In Table 2, alpha diversity statistics indicate that fungal communities became more diverse, whereas bacterial communities became more similar, with *P*-values approaching 1.

### At-harvest

Grain drying is a key part of wheat agricultural production, where the wheat crop is allowed to dry out prior to harvest. This period sees the plant die and go through senescence after seeding at the end of the WGP. Redundancy based analyses revealed that while fungal communities (Figs. 2 and 3) returned to their ratios at the start of the season, genera such as *Pseudocoleophoma*, a genus described as having both endophytic and saprophytic lifestyles (Lu et al. 2022), and *Paramyrothecium*, a fungal genus commonly associated with plant diseases and commonly occurring on decaying plant matter and in soil (Aumentado et al. 2024), were more enriched. Fungi occurring in WC soils showed the quickest return to bulk soil taxa ratios after the death of the wheat crop, suggesting that even though the selection pressure of wheat may suppress these genera from dominating in one soil over another, they are ready to re-emerge once that pressure abates. Bacterial communities, however, remained more consistent in their taxa ratios AH, with results specific to each farm. While all bacteria saw a decrease in relative abundance AH, on Langgewens *Geodermatophilus*, a grassland associated bacterium with soil protective properties, bioremediation capabilities, and the ability to withstand extreme environments (Castro et al. 2018, Ren et al. 2023), was found to be enriched AH, especially in WC soils. However, on Tygerhoek, we found that only *Bacillus* was still enriched at significant numbers compared to the PP time-point. *Bacillus* was also now more significant in WM and WC soils than in WW soils, the inverse of what was seen at PP.

Co-occurrence analyses, in Figs. S3 and 4, revealed that core-communities AH shifted towards yeasts and agents of decay. These core nodes consisted of *Alternaria*, a genus typically associated with decay and decomposition (Dang et al. 2015), *Vishniacozyma*, a saprophytic yeast related to increased temperatures (Newsham et al. 2022), *Nakamurella*, a genus associated with plant protective capabilities against pathogens (Lazcano et al. 2021), and *Didymellaceae*_unclassified, a family of generalists, from saprobes to plant pathogens, occupying a variety of environments (Hou et al. 2020).

Functional analyses (Figs. 5 and 6) revealed that AH, treatments displayed the greatest shift in functional groups. However, these were once again specific to each farm. While saprophytes across the farms increased in relative abundance, those associated with WC soils showed the least increase in relative abundance, with WW and WM soils being the most similar. When bacterial functional groups were evaluated, those associated with the nitrogen cycle showed the greatest change from the WGP. On Langgewens, WW and WM soils were the most abundant for these groups, whereas WC soils were less abundant. Nitrate reduction was significantly more abundant in WM soils, followed by WW and WC soils. On, Tygerhoek, this was reflected, however, functional groups for nitrate reduction were not significantly different. This may be explained by different nitrogen fertilizers used by the different farms, where Tygerhoek utilizes nitrate-based fertilizers (Timac D Coder) and Langgewens uses ammonium-based fertilizers (GeoFlo), which may have selected for nitrate-reducing microbes in all treatments on Tygerhoek. Interestingly, nitrate-, nitrite-, and overall nitrogen respiration, processes involved with nitrogen loss to the atmosphere through denitrification, or dissimilatory nitrate reduction to ammonium, was significantly increased in WW soils on both farms AH. Nitrogen loss and ammonium production during plant senescence are not surprising. However, these groups being significantly more abundant in WW soils and not WC and WM soils is noteworthy and should be further investigated.

### Environmental correlations

Environmental correlation analyses revealed no significant correlations between functional groups and soil geochemical properties or qPCR gene-count values. However, correlations between these environmental measurements and individual species were observed to be significant (*P* < .001). Urease activity had the largest number of individual genera associated with it, compared to all other metrics. *Dothideomycetes* have previously been associated with urease degradation capability in soils by Alizadeh et al. (2017), with Strope et al. (2011) finding that they had the genetic capability to do so. Furthermore, Wang and Xiong (2022) have previously shown that ureolytic communities are often dominated by Alphaproteobacteria and Gammaproteobacteria. Lastly, urease activity has long been associated with Bacilli (Kim et al. 2005, Wright et al. 2020). Our analysis also revealed a strong negative correlation between genera *Vishniacozyma* and *Alternaria* and soil moisture. Considering that both these saprobes were dominant in the post-drying period AH, this does not come as a surprize.

qPCR analyses revealed trends in gene copy variation on Langgewens; a spike in gene copies associated with nitrification (*amoA* and *nxrB*) during GM is unsurprising as Langgewens uses ammonium-based nitrogen fertilizers. The conversion of ammonium to nitrite (*amoA*) and the subsequent conversion of nitrite to nitrate (*nxrB*), which the crops can absorb, is ideal. Unfortunately, as an effect of nitrite production, *nirS* and *nirK*, genes associated with the denitrification of nitrite to nitric oxide, were increased. Meaning nitrogen was lost to the atmosphere. Following GM, gene copies of *amoA* decreased, most likely due to a depletion in ammonium from the soil, i.e. complete depletion of nitrite. Gene copies of *nirK* also decreased, however gene copies of *nirS* and *nxrB* simply plateaued, suggesting rather than a decrease due to a depletion of nitrites in the soil, but a shift in metabolism. Lastly, gene copies of *nifH*, associated with nitrogen fixation, and of *napA*, associated with the denitrification of nitrate to nitrite, increased throughout the season.

### Drought-induced bacterial selection

The rhizosphere microbiome plays a critical role in enhancing crop resilience against abiotic stressors such as drought (Zolla et al. 2013, Ngumbi and Kloepper 2016, Eke et al. 2019) and biotic stressors such as pathogens (Mendes et al. 2013, Lazcano et al. 2021, Li et al. 2021, Xun et al. 2021, Gu et al. 2022). Subsequently, modifications to the rhizobiome are indicative of ongoing stressors. Previous studies have reported that the dominant rhizosphere microbes of wheat under normal conditions, in order of abundance, consisted of bacteria: Pseudomonadota (Alphaproteobacteria, Gammaproteobacteria, Deltaproteobacteria, and Betaproteobacteria), Actinomycetota (*Actinobacteria* and *Thermophilia*), Acidobacteriota (Acidimicrobiia) Bacteroidota (Sphingobacteriia), Bacillota (Bacilli), and Chloroflexota (Chloroflexi) (Araujo et al. 2019, Chen et al. 2019, Cordero et al. 2021, Dilla-Ermita et al. 2021, Xun et al. 2021). While the results in this study are mostly congruent with the consensus, various differences exist. In this study, we found that classes Actinobacteria and Alphaproteobacteria were significantly enriched. An increased relative abundance of Actinobacteria has been reported in multiple studies investigating the effects of drought stress on rhizosphere bacteria in different crops. This could be the result of their production of antibiotic compounds, either by granting them greater competitive fitness over other bacteria, or being selected by crops as a mitigation effort against the increased prevalence of pathogens under environmental stress conditions (Naylor et al. 2017, Santos-Medellín et al. 2017, Naylor and Coleman-Derr 2018, Gebauer et al. 2022, Hu et al. 2023). Furthermore, a study by Zhang et al. (2021) found that the genera *Nocardioides, Streptomyces*, and *Marmoricola* increased in relative abundance under drought conditions, echoing the results in this study. Alternative reasons for the enrichment of Actinobacteria relevant to this study include wheat selection (Kawasaki et al. 2016) and reduced fertilizer application (Ai et al. 2015, Chen et al. 2019, Jacquiod et al. 2022).

Alphaproteobacteria has been identified as a central class during droughts (Santos-Medellín et al. 2017, Liu et al. 2021b). Here, we found that the Alphaproteobacteria genera, *Microvirga* and *Sphingomonas*, were dominant in the rhizosphere. *Microvirga* species are considered thermo- and drought-tolerant rhizobia due to their ability to form root nodules and fix nitrogen under extreme conditions, potentially explaining their dominance in the drought-stressed rhizosphere (Zilli et al. 2015, Li et al. 2020, Hungria and Nogueira 2023). Additionally, *Spingomonas* species have been shown to be plant growth promoting rhizobacteria (PGPR), that can confer drought and osmotic stress tolerance towards a host plant, while others can fix nitrogen (Videira et al. 2009, Asaf et al. 2017, Luo et al. 2019, Asaf et al. 2020). The enrichment of these Alphaproteobacterial genera may also be due to drought-induced phytohormone cascades, that not only stimulate root growth, but also induce increased plant defence mechanisms against biotic and abiotic stressors, such as increased production of epicuticular wax, osmolytes, and antioxidants (Asaf et al. 2017, Carlson et al. 2020, Ahmad et al. 2022, Asghar et al. 2022). These genera are suggested to comprise species that exhibit drought tolerance. Nevertheless, functional validation is required to substantiate their contribution in a regenerative agricultural context. Various studies have also reported drought-induced enrichment of known PGPR. *Bacillus*, a dominant genus at the start of the season in this study; however, significantly decreases over the season, as if selected against by the wheat plant or being outcompeted by other taxa more adaptable to the rhizosphere environment (Naylor and Coleman-Derr 2018, Dai et al. 2019, Liu et al. 2021b). Interestingly, a study by Rashid et al. (2022) found that *Bacillus megaterium* had the ability to confer drought tolerance in wheat plants by increasing plant biomass and relative water retention. However, this does not appear to be the case in this study as we noted a decrease in relative abundance of *Bacillus* spp. over the season, perhaps due to increased rainfall over the sampling period.

In conclusion, we investigated the effects of crop rotation treatments under transitional regenerative management practices in South Africa. We found that monocrop rotation systems resulted in significant beta diversity differences between crops for bacterial and fungal communities. This study found a dominating selection pressure from the current host crop, wheat, with all communities trending towards similarity, supporting our second hypothesis. This selection pressure, however, was not maintained once the crop senesces during the grain drying period before harvest, with communities drifting away from one another, as saprobes came to dominate the communities. Additionally, we found increases in the number of microbial taxa associated with extreme environments, such as deserts, supporting our third hypothesis. Functional analyses revealed that groups associated with the nitrogen cycle significantly increased in WW and WM treatments compared to WC. Additionally, we found that fungal functional groups associated with saprophytic and pathogenic groups were more significantly associated with WM. Lastly, we found that WW and WM soils had the greatest overlap in microbial communities, with WC soils showing the most divergence. Although this study set out to investigate the effects of crop-rotation systems on the rhizosphere soils in wheat, the dominant selective pressure from the host plant overshadowed any potential impact of crop rotation effects or benefits. There was, however, a change in the abundance of functional groups associated with the nitrogen cycle, which correlated to the previous rotation. Ideally, consecutive years should have been investigated to establish the preceding year, establish the carry-over effect to the next season, and determine the connected taxa selected by the previous crop and incorporated into the wheat rhizosphere. This study hopes to contribute to the growing knowledge base of the effects regenerative agricultural practices have on the soil microbiome and to elucidate its mitigating factors on droughts, in an ever-changing climate, and to help contribute to and refine SDG goals referenced in our sustainability statement.

## Supplementary Material

qvaf020_Supplemental_File

## Data Availability

The DNA sequences from all incubation samples are deposited in the NCBI Sequence Read Archive (SRA) database with accession number PRJNA818831.
