# Supplementary material for: The effect of crop-rotation systems in regenerative agricultural environments on the rhizosphere microbiome of dry-land winter wheat
Source: Sustain Microbiol. 2025 Aug 29;2(4):qvaf020. doi: 10.1093/sumbio/qvaf020 (PMC13375304; doi:10.1093/sumbio/qvaf020)
Supplement: Supplementary file 1 [file qvaf020_Supplemental_File.docx]

**The effect of crop-rotation systems in regenerative agricultural environments on the rhizosphere microbiome of dry-land winter wheat.**

Supporting Figures and Tables

**Supplementary Figure 1** – Wheat agriculture cycle. White blocks representing the sampling time-points in this study. Green blocks representing the changes in crop lifestyle between sampling periods.

**Supplementary Figure 2** - Stacked bar graph of the top ten (10) (A) bacterial and (B) fungal phyla present in the wheat rhizosphere, for the 2019 agricultural season, grouped by farm, treatment, and time point. Key: Treatments: WW = wheat after wheat, WC = wheat after canola, WM = wheat after medicago; Time points: PP = Pre-plant, GM = germination, TD = top dressing, AH = At-harvest.

**Supplementary Figure 3** - Co-Occurrence network analyses of the core wheat microbiome by treatment – (A) total pre-plant network, (B) wheat after wheat pre-plant network, (C) wheat after medicago pre-plant network, & (D) wheat after canola pre-plant network. Nodes indicated by circles, edges indicated by lines – Green lines indicate positive correlations, red lines indicate negative correlations.

**Supplementary Figure 4** - Pre-plant Bacterial and Fungal Elemental Correlation Heat Map. Blue indicating negative correlations, red indicating positive correlations. Key: H = hydrogen, Na = sodium, C = carbon, K = potassium, Ca = calcium, Mg = magnesium, Soluble.S = soluble sulfur.

**Supplementary Figure 5** - Pre-plant Bacterial and Fungal Enzyme Correlation Heat Map. Blue indicating negative correlations, red indicating positive correlations. Key: EC = electrical conductivity, AvailableP = available phosphates, Nmin = nitrogen mineralization.

| Supplementary Table 1 – Co-occurrence analyses of the microbial communities, broken down by timepoint, farm, and treatment depicting relative abundance, node degree, weighted degree, closeness centrality, harmonic closeness centrality, betweenness closeness centrality, and network triangle analyses. | | | | | | | | | |
| --- | --- | --- | --- | --- | --- | --- | --- | --- | --- |
| OTU | **Phylum** | **Genus** | **Relative Abundance** | **Degree** | **Weighted Degree** | **Closeness Centrality** | **Harmonic Closeness Centrality** | **Betweenness Centrality** | **Triangles** |
| Total Pre-Plant (Bulk Soil) Networks | | | | | | | | | |
| F_Otu0004 | Ascomycota | Alternaria | 0.299677 | 8 | 0.227769 | 0.291793 | 0.358507 | 0.256626 | 6 |
| F_Otu0108 | Ascomycota | Exophiala | 0.183531 | 7 | 0.21956 | 0.258065 | 0.32066 | 0.060025 | 5 |
| B_Otu0022 | Actinomycetota | Rubrobacter | 0.756585 | 6 | 0.430818 | 0.239401 | 0.308172 | 0.111093 | 2 |
| F_Otu0024 | Ascomycota | Plectosphaerella | 0.369093 | 6 | 0.532687 | 0.224299 | 0.29902 | 0.188333 | 0 |
| F_Otu0035 | Ascomycota | Schizothecium | 0.348099 | 6 | 0.297001 | 0.256684 | 0.318428 | 0.147373 | 2 |
| B_Otu0019 | Actinomycetota | Blastococcus | 0.278795 | 6 | 0.506468 | 0.226415 | 0.292113 | 0.093781 | 1 |
| F_Otu0121 | Ascomycota | Knufia | 0.184547 | 6 | 0.224909 | 0.235294 | 0.304874 | 0.124654 | 1 |
| F_Otu0069 | Ascomycota | Lectera | 0.18116 | 6 | 0.292701 | 0.247423 | 0.314906 | 0.176292 | 1 |
| F_Otu0056 | Ascomycota | Phoma | 0.168406 | 6 | 0.440368 | 0.283186 | 0.342708 | 0.243461 | 2 |
| B_Otu0069 | Gemmatimonadota | Longimicrobiaceae_ge | 0.138382 | 6 | 0.258758 | 0.239401 | 0.302207 | 0.100215 | 2 |
| B_Otu0048 | Pseudomonadota | MND1 | 0.384444 | 5 | 0.533443 | 0.267409 | 0.324578 | 0.224379 | 1 |
| F_Otu0065 | Ascomycota | Hormiactis | 0.306223 | 5 | 0.098493 | 0.21286 | 0.270523 | 0.114978 | 1 |
| F_Otu0130 | Kickxellomycota | Ramicandelaber | 0.278569 | 5 | 0.098283 | 0.27907 | 0.331944 | 0.173104 | 2 |
| F_Otu0090 | Ascomycota | Stachybotrys | 0.210959 | 5 | 0.107697 | 0.244275 | 0.303671 | 0.154347 | 1 |
| F_Otu0041 | Ascomycota | Rodentomyces | 0.105987 | 5 | 0.525184 | 0.238213 | 0.296329 | 0.066086 | 2 |
| B_Otu0011 | Acidobacteriota | Bryobacter | 0.639197 | 4 | 0.438836 | 0.20296 | 0.261376 | 0.051059 | 0 |
| B_Otu0026 | Actinomycetota | Actinomycetospora | 0.239967 | 4 | 0.360717 | 0.246787 | 0.30119 | 0.141857 | 0 |
| B_Otu0079 | Actinomycetota | Kribbella | 0.235114 | 4 | 0.182232 | 0.221198 | 0.273127 | 0.043426 | 0 |
| F_Otu0113 | Ascomycota | Spiromastix | 0.214684 | 4 | 0.224492 | 0.191235 | 0.23991 | 0.029202 | 0 |
| B_Otu0091 | Actinomycetota | 0319-7L14_ge | 0.198769 | 4 | 0.229755 | 0.20915 | 0.267138 | 0.045728 | 1 |
| B_Otu0056 | Pseudomonadota | BIrii41_ge | 0.194592 | 4 | 0.452267 | 0.201681 | 0.258036 | 0.044112 | 0 |
| B_Otu0093 | Pseudomonadota | Reyranella | 0.167954 | 4 | 0.140386 | 0.30094 | 0.347049 | 0.274822 | 1 |
| F_Otu0204 | Ascomycota | Humicola | 0.132399 | 4 | 0.330024 | 0.191617 | 0.243597 | 0.051925 | 0 |
| F_Otu0002 | Ascomycota | Fusarium | 7.021914 | 3 | 0.069257 | 0.25 | 0.300124 | 0.085199 | 0 |
| F_Otu0019 | Basidiomycota | Solicoccozyma | 2.359149 | 3 | 0.315307 | 0.21968 | 0.26276 | 0.007436 | 0 |
| F_Otu0030 | Ascomycota | Aspergillus | 1.261915 | 3 | 0.05869 | 0.185687 | 0.235987 | 0.03777 | 1 |
| F_Otu0023 | Ascomycota | Pseudopyrenochaeta | 0.843722 | 3 | 0.184224 | 0.228571 | 0.284028 | 0.103447 | 0 |
| B_Otu0007 | Actinomycetota | Mycobacterium | 0.803427 | 3 | 0.115379 | 0.226415 | 0.272371 | 0.010779 | 1 |
| F_Otu0052 | Ascomycota | Metarhizium | 0.737848 | 3 | 0.311522 | 0.233577 | 0.276414 | 0.006675 | 1 |
| B_Otu0006 | Pseudomonadota | Microvirga | 0.643825 | 3 | 0.258972 | 0.213808 | 0.259945 | 0.032916 | 0 |
| B_Otu0020 | Actinomycetota | Marmoricola | 0.367174 | 3 | 0.407667 | 0.218679 | 0.264807 | 0.069583 | 0 |
| B_Otu0057 | Actinomycetota | Gaiella | 0.308029 | 3 | 0.205352 | 0.224299 | 0.264559 | 0.038048 | 0 |
| B_Otu0034 | Gemmatimonadota | Gemmatimonas | 0.302724 | 3 | 0.071759 | 0.190476 | 0.247495 | 0.040004 | 0 |
| B_Otu0052 | Pseudomonadota | Haliangium | 0.282746 | 3 | 0.422131 | 0.178771 | 0.226889 | 0.025969 | 0 |
| F_Otu0126 | Ascomycota | Periconia | 0.2613 | 3 | 0.266512 | 0.184971 | 0.233383 | 0.041447 | 0 |
| B_Otu0046 | Pseudomonadota | 0319-6G20_ge | 0.238048 | 3 | 0.058695 | 0.200418 | 0.242696 | 0.009341 | 1 |
| F_Otu0099 | Ascomycota | Emericellopsis | 0.215135 | 3 | 0.12102 | 0.193159 | 0.242638 | 0.048969 | 0 |
| B_Otu0068 | Actinomycetota | IMCC26256_ge | 0.192335 | 3 | 0.07639 | 0.195122 | 0.237512 | 0.023794 | 0 |
| B_Otu0074 | Acidobacteriota | Candidatus_Solibacter | 0.190529 | 3 | 0.290382 | 0.223776 | 0.265365 | 0.024048 | 0 |
| B_Otu0080 | Pseudomonadota | Rhodoplanes | 0.188497 | 3 | 0.282852 | 0.186408 | 0.23769 | 0.020496 | 0 |
| B_Otu0098 | Actinomycetota | Actinophytocola | 0.183531 | 3 | 0.211876 | 0.238213 | 0.2812 | 0.025079 | 1 |
| B_Otu0086 | Pseudomonadota | TRA3-20_ge | 0.172808 | 3 | 0.164096 | 0.21968 | 0.262438 | 0.032248 | 0 |
| B_Otu0035 | Actinomycetota | Geodermatophilus | 0.166261 | 3 | 0.351609 | 0.22069 | 0.268328 | 0.038381 | 1 |
| F_Otu0086 | Ascomycota | Setophoma | 0.161972 | 3 | 0.116162 | 0.246787 | 0.291865 | 0.038985 | 0 |
| F_Otu0209 | Ascomycota | Purpureocillium | 0.153168 | 3 | 0.303014 | 0.160267 | 0.203844 | 0.004375 | 1 |
| F_Otu0221 | Ascomycota | Blastobotrys | 0.132625 | 3 | 0.308586 | 0.192771 | 0.242134 | 0.031382 | 0 |
| B_Otu0122 | Actinomycetota | Actinoallomurus | 0.113324 | 3 | 0.099316 | 0.236453 | 0.281622 | 0.004812 | 2 |
| B_Otu0084 | Pseudomonadota | Pseudolabrys | 0.10452 | 3 | 0.011033 | 0.210989 | 0.256634 | 0.027856 | 0 |
| B_Otu0003 | Actinomycetota | 67-14_ge | 1.61419 | 2 | 0.054587 | 0.185687 | 0.217572 | 0.000877 | 0 |
| F_Otu0014 | Ascomycota | Preussia | 1.408537 | 2 | 0.225882 | 0.181818 | 0.207056 | 0.020833 | 0 |
| B_Otu0001 | Actinomycetota | Nocardioides | 1.292165 | 2 | 0.180326 | 0.181132 | 0.214199 | 0.041228 | 0 |
| B_Otu0005 | Actinomycetota | Streptomyces | 1.037299 | 2 | 0.073122 | 0.154093 | 0.180985 | 0.020833 | 0 |
| F_Otu0027 | Ascomycota | Chaetomium | 0.772613 | 2 | 0.136066 | 0.208696 | 0.248537 | 0.022941 | 0 |
| B_Otu0008 | Pseudomonadota | Sphingomonas | 0.647324 | 2 | 0.139502 | 0.176147 | 0.209813 | 0 | 1 |
| F_Otu0020 | Ascomycota | Paramyrothecium | 0.560525 | 2 | 0.114323 | 0.184261 | 0.226438 | 0.020833 | 0 |
| B_Otu0015 | Actinomycetota | Solirubrobacter | 0.464132 | 2 | 0.186148 | 0.184971 | 0.220081 | 0.009143 | 0 |
| B_Otu0031 | Bacillota | Sporosarcina | 0.412549 | 2 | 0.084179 | 0.176471 | 0.211549 | 0.020833 | 0 |
| B_Otu0021 | Pseudomonadota | Skermanella | 0.411759 | 2 | 0.245219 | 0.207792 | 0.2516 | 0.024182 | 0 |
| F_Otu0129 | Ascomycota | Dichotomopilus | 0.377333 | 2 | 0.03209 | 0.217195 | 0.25935 | 0.037469 | 0 |
| F_Otu0128 | Ascomycota | Dactylonectria | 0.260171 | 2 | 0.005593 | 0.191617 | 0.230746 | 0.020833 | 0 |
| F_Otu0119 | Basidiomycota | Schizophyllum | 0.222133 | 2 | 0.03619 | 0.189723 | 0.229555 | 0.013973 | 0 |
| F_Otu0118 | Ascomycota | Sporormiella | 0.197753 | 2 | 0.212182 | 0.22069 | 0.252927 | 0.041228 | 0 |
| B_Otu0054 | Actinomycetota | Pseudarthrobacter | 0.18037 | 2 | 0.136366 | 0.198758 | 0.239823 | 0 | 1 |
| B_Otu0102 | Pseudomonadota | CCD24_ge | 0.176758 | 2 | 0.319239 | 0.218182 | 0.255667 | 0.019085 | 0 |
| B_Otu0094 | Pseudomonadota | Steroidobacter | 0.174162 | 2 | 0.408149 | 0.173913 | 0.214253 | 0.000439 | 0 |
| F_Otu0100 | Ascomycota | Gibellulopsis | 0.167051 | 2 | 0.129861 | 0.176147 | 0.209813 | 0 | 1 |
| F_Otu0101 | Ascomycota | Coniothyrium | 0.165584 | 2 | 0.245323 | 0.193548 | 0.227964 | 0.000786 | 0 |
| F_Otu0045 | Ascomycota | Pseudocoleophoma | 0.164342 | 2 | 0.065113 | 0.199584 | 0.237339 | 0.002412 | 0 |
| F_Otu0156 | Ascomycota | Wardomycopsis | 0.164342 | 2 | 0.144118 | 0.163543 | 0.201567 | 0.005914 | 0 |
| B_Otu0099 | Acidobacteriota | Subgroup_7_ge | 0.143912 | 2 | 0.044362 | 0.192385 | 0.227505 | 0.006621 | 0 |
| B_Otu0082 | Actinomycetota | Crossiella | 0.142332 | 2 | 0.270754 | 0.205567 | 0.249297 | 0.012226 | 0 |
| F_Otu0201 | Ascomycota | Ophiocordyceps | 0.138946 | 2 | 0.26193 | 0.158416 | 0.194122 | 0 | 1 |
| B_Otu0063 | Bacteroidota | Flavisolibacter | 0.128223 | 2 | 0.162752 | 0.195519 | 0.231709 | 0 | 1 |
| B_Otu0083 | Pseudomonadota | Candidatus_Alysiosphaera | 0.112534 | 2 | 0.192507 | 0.192771 | 0.228373 | 0.002053 | 0 |
| F_Otu0192 | Basidiomycota | Atractiella | 0.108922 | 2 | 0.172676 | 0.210066 | 0.250112 | 0.052911 | 0 |
| B_Otu0090 | Actinomycetota | Conexibacter | 0.10689 | 2 | 0.262439 | 0.177122 | 0.21756 | 0.006871 | 0 |
| B_Otu0009 | Bacillota | Bacillus | 0.866635 | 1 | 0.042039 | 0.150235 | 0.171718 | 0 | 0 |
| F_Otu0021 | Ascomycota | Clonostachys | 0.747442 | 1 | 0.004309 | 0.161074 | 0.184396 | 0 | 0 |
| F_Otu0057 | Basidiomycota | Trechispora | 0.4884 | 1 | 0.036772 | 0.184971 | 0.216501 | 0 | 0 |
| F_Otu0048 | Ascomycota | Neocosmospora | 0.445056 | 1 | 0.015091 | 0.160804 | 0.186909 | 0 | 0 |
| B_Otu0018 | Actinomycetota | Pseudonocardia | 0.428238 | 1 | 0.032775 | 0.133705 | 0.151476 | 0 | 0 |
| F_Otu0076 | Ascomycota | Trichoderma | 0.366046 | 1 | 0.04236 | 0.156352 | 0.183705 | 0 | 0 |
| F_Otu0087 | Basidiomycota | Geminibasidium | 0.362208 | 1 | 0.019983 | 0.154093 | 0.17042 | 0 | 0 |
| B_Otu0042 | Pseudomonadota | Acidibacter | 0.349341 | 1 | 0.134738 | 0.151899 | 0.17908 | 0 | 0 |
| F_Otu0097 | Basidiomycota | Leucocoprinus | 0.317623 | 1 | 0.091209 | 0.183556 | 0.205419 | 0 | 0 |
| F_Otu0071 | Ascomycota | Darksidea | 0.288389 | 1 | 0.104485 | 0.155844 | 0.181101 | 0 | 0 |
| B_Otu0053 | Pseudomonadota | SC-I-84_ge | 0.247417 | 1 | 0.319896 | 0.169014 | 0.199686 | 0 | 0 |
| F_Otu0105 | Ascomycota | Sagenomella | 0.224955 | 1 | 0.027582 | 0.196721 | 0.225248 | 0 | 0 |
| F_Otu0073 | Ascomycota | Lophiotrema | 0.220101 | 1 | 0.093941 | 0.198758 | 0.230394 | 0 | 0 |
| B_Otu0065 | Nitrospirotae | Nitrospirota | 0.206557 | 1 | 0.015729 | 0.163543 | 0.187223 | 0 | 0 |
| F_Otu0114 | Chytridiomycota | Rhizophlyctis | 0.200236 | 1 | 0.037018 | 0.156352 | 0.183705 | 0 | 0 |
| F_Otu0139 | Ascomycota | Talaromyces | 0.139736 | 1 | 0.186842 | 0.183556 | 0.219494 | 0 | 0 |
| F_Otu0064 | Ascomycota | Caliciopsis | 0.134205 | 1 | 0.074563 | 0.205567 | 0.233581 | 0 | 0 |
| F_Otu0301 | Ascomycota | Acremonium | 0.130368 | 1 | 0.06858 | 0.162162 | 0.189757 | 0 | 0 |
| F_Otu0124 | Basidiomycota | Entoloma | 0.128223 | 1 | 0.040176 | 0.184971 | 0.216501 | 0 | 0 |
| F_Otu0202 | Ascomycota | Torula | 0.125063 | 1 | 0.075484 | 0.190855 | 0.223801 | 0 | 0 |
| B_Otu0111 | Pseudomonadota | Bradyrhizobium | 0.105536 | 1 | 0.074161 | 0.161074 | 0.188967 | 0 | 0 |
| Pre-Plant KK Networks | | | | | | | | | |
| F_Otu0023 | Ascomycota | Pseudopyrenochaeta | 0.176212 | 6 | 0.249914 | 0.190751 | 0.292834 | 0.283208 | 0 |
| F_Otu0108 | Ascomycota | Exophiala | 0.285022 | 5 | 0.405974 | 0.176471 | 0.273005 | 0.179754 | 0 |
| F_Otu0052 | Ascomycota | Metarhizium | 1.10563 | 4 | 0.267013 | 0.183333 | 0.273463 | 0.098398 | 0 |
| B_Otu0009 | Bacillota | Bacillus | 1.409694 | 3 | 0.207681 | 0.138655 | 0.221857 | 0.095006 | 0 |
| F_Otu0076 | Ascomycota | Trichoderma | 0.796429 | 3 | 0.185945 | 0.152074 | 0.233987 | 0.071161 | 0 |
| B_Otu0007 | Actinomycetota | Mycobacterium | 0.719657 | 3 | 0.194314 | 0.167513 | 0.257291 | 0.184894 | 0 |
| B_Otu0011 | Acidobacteriota | Bryobacter | 0.678249 | 3 | 0.181147 | 0.098068 | 0.17258 | 0.039576 | 0 |
| F_Otu0035 | Ascomycota | Schizothecium | 0.524706 | 3 | 0.035765 | 0.179348 | 0.256291 | 0.051415 | 0 |
| B_Otu0015 | Actinomycetota | Solirubrobacter | 0.507175 | 3 | 0.244311 | 0.8 | 0.875 | 0.001248 | 0 |
| B_Otu0042 | Pseudomonadota | Acidibacter | 0.472719 | 3 | 0.149486 | 0.1375 | 0.205347 | 0.047337 | 0 |
| B_Otu0031 | Bacillota | Sporosarcina | 0.429799 | 3 | 0.294112 | 0.125714 | 0.205983 | 0.076654 | 0 |
| F_Otu0139 | Ascomycota | Talaromyces | 0.355446 | 3 | 0.124175 | 0.118919 | 0.184603 | 0.04769 | 0 |
| F_Otu0121 | Ascomycota | Knufia | 0.349703 | 3 | 0.151433 | 0.107143 | 0.188582 | 0.082647 | 0 |
| B_Otu0021 | Pseudomonadota | Skermanella | 0.339729 | 3 | 0.177716 | 1 | 1 | 0.000499 | 1 |
| B_Otu0020 | Actinomycetota | Marmoricola | 0.314038 | 3 | 0.164588 | 0.127413 | 0.203028 | 0.177528 | 0 |
| B_Otu0052 | Pseudomonadota | Haliangium | 0.308899 | 3 | 0.228899 | 0.175532 | 0.245966 | 0.26263 | 0 |
| B_Otu0056 | Pseudomonadota | BIrii41_ge | 0.239684 | 3 | 0.286095 | 0.154567 | 0.223018 | 0.069496 | 0 |
| B_Otu0068 | Actinomycetota | IMCC26256_ge | 0.180745 | 3 | 0.086124 | 0.150342 | 0.232742 | 0.124345 | 0 |
| B_Otu0091 | Actinomycetota | 0319-7L14_ge | 0.156565 | 3 | 0.23714 | 1 | 1 | 0.000749 | 0 |
| B_Otu0069 | Gemmatimonadota | Longimicrobiaceae_ge | 0.139337 | 3 | 0.108493 | 0.117229 | 0.199833 | 0.142572 | 0 |
| F_Otu0192 | Basidiomycota | Atractiella | 0.132083 | 3 | 0.117535 | 0.173684 | 0.253387 | 0.138577 | 0 |
| F_Otu0204 | Ascomycota | Humicola | 0.126038 | 3 | 0.414724 | 0.150342 | 0.217371 | 0.04769 | 0 |
| F_Otu0102 | Chytridiomycota | Clydaea | 0.119086 | 3 | 0.146843 | 0.140426 | 0.222193 | 0.053184 | 0 |
| F_Otu0349 | Ascomycota | Thelonectria | 0.112739 | 3 | 0.054137 | 0.162162 | 0.225267 | 0.211985 | 0 |
| F_Otu0019 | Basidiomycota | Solicoccozyma | 3.091715 | 2 | 0.112006 | 0.156028 | 0.21416 | 0.000395 | 0 |
| F_Otu0005 | Ascomycota | Penicillium | 2.526507 | 2 | 0.150339 | 0.133065 | 0.199839 | 0.061923 | 0 |
| B_Otu0003 | Actinomycetota | 67-14_ge | 1.555681 | 2 | 0.229302 | 0.133603 | 0.19818 | 0.041698 | 0 |
| F_Otu0030 | Ascomycota | Aspergillus | 1.481931 | 2 | 0.027314 | 0.106109 | 0.170906 | 0.036954 | 0 |
| F_Otu0014 | Ascomycota | Preussia | 1.305418 | 2 | 0.292632 | 0.113597 | 0.169025 | 0.01623 | 0 |
| F_Otu0046 | Ascomycota | Beauveria | 0.879245 | 2 | 0.378301 | 0.121547 | 0.179618 | 0.029338 | 0 |
| B_Otu0022 | Actinomycetota | Rubrobacter | 0.573368 | 2 | 0.078576 | 0.75 | 0.833333 | 0 | 1 |
| F_Otu0065 | Ascomycota | Hormiactis | 0.502642 | 2 | 0.060312 | 0.106796 | 0.157532 | 0.01623 | 0 |
| F_Otu0119 | Basidiomycota | Schizophyllum | 0.459722 | 2 | 0.149476 | 0.147982 | 0.204002 | 0.073159 | 0 |
| F_Otu0126 | Ascomycota | Periconia | 0.457002 | 2 | 0.183605 | 0.141935 | 0.203768 | 0.068414 | 0 |
| B_Otu0048 | Pseudomonadota | MND1 | 0.433729 | 2 | 0.113761 | 0.137787 | 0.188374 | 0.01702 | 0 |
| F_Otu0113 | Ascomycota | Spiromastix | 0.420127 | 2 | 0.243139 | 0.083439 | 0.140032 | 0.001748 | 0 |
| F_Otu0220 | Ascomycota | Dichotomopilus | 0.414082 | 2 | 0.117277 | 0.097202 | 0.156671 | 0.01623 | 0 |
| F_Otu0004 | Ascomycota | Alternaria | 0.395343 | 2 | 0.085348 | 0.178378 | 0.24619 | 0.044049 | 0 |
| F_Otu0180 | Ascomycota | Epicoccum | 0.274141 | 2 | 0.171677 | 0.113793 | 0.178942 | 0.020474 | 0 |
| F_Otu0128 | Ascomycota | Dactylonectria | 0.250263 | 2 | 0.149665 | 0.131474 | 0.180861 | 0.01623 | 0 |
| F_Otu0031 | Ascomycota | Cladosporium | 0.242404 | 2 | 0.037643 | 0.157895 | 0.217714 | 0.08015 | 0 |
| F_Otu0048 | Ascomycota | Neocosmospora | 0.242102 | 2 | 0.080803 | 0.089431 | 0.144712 | 0.00824 | 0 |
| F_Otu0049 | Ascomycota | Microdochium | 0.238173 | 2 | 0.017389 | 0.096916 | 0.15465 | 0.022347 | 0 |
| B_Otu0074 | Acidobacteriota | Candidatus_Solibacter | 0.206739 | 2 | 0.035423 | 0.149321 | 0.204349 | 0.191011 | 0 |
| B_Otu0094 | Pseudomonadota | Steroidobacter | 0.206134 | 2 | 0.196494 | 0.159806 | 0.222728 | 0.033625 | 0 |
| B_Otu0079 | Actinomycetota | Kribbella | 0.198276 | 2 | 0.123995 | 0.101695 | 0.152753 | 0.01623 | 0 |
| B_Otu0065 | Nitrospirotae | Nitrospirota | 0.192835 | 2 | 0.168953 | 1 | 1 | 0.00025 | 0 |
| F_Otu0086 | Ascomycota | Setophoma | 0.187093 | 2 | 0.063968 | 0.105939 | 0.163558 | 0.009238 | 0 |
| B_Otu0102 | Pseudomonadota | CCD24_ge | 0.181048 | 2 | 0.059088 | 0.133603 | 0.187022 | 0.014544 | 0 |
| B_Otu0093 | Pseudomonadota | Reyranella | 0.171376 | 2 | 0.220934 | 0.112628 | 0.174793 | 0.03196 | 0 |
| F_Otu0201 | Ascomycota | Ophiocordyceps | 0.160495 | 2 | 0.105984 | 0.666667 | 0.75 | 0.000749 | 0 |
| F_Otu0090 | Ascomycota | Stachybotrys | 0.154147 | 2 | 0.113043 | 0.1056 | 0.165625 | 0.009863 | 0 |
| F_Otu0221 | Ascomycota | Blastobotrys | 0.153543 | 2 | 0.263937 | 0.090164 | 0.151783 | 0.009488 | 0 |
| B_Otu0063 | Bacteroidota | Flavisolibacter | 0.149009 | 2 | 0.156466 | 0.75 | 0.833333 | 0 | 1 |
| B_Otu0111 | Pseudomonadota | Bradyrhizobium | 0.1209 | 2 | 0.044897 | 0.182825 | 0.248569 | 0.232667 | 0 |
| F_Otu0062 | Ascomycota | Articulospora | 0.116366 | 2 | 0.27483 | 0.142241 | 0.197467 | 0.023013 | 0 |
| B_Otu0122 | Actinomycetota | Actinoallomurus | 0.115762 | 2 | 0.213499 | 0.112436 | 0.168757 | 0.018227 | 0 |
| B_Otu0140 | Pseudomonadota | Dongia | 0.105787 | 2 | 0.127438 | 0.121547 | 0.171568 | 0.01623 | 0 |
| B_Otu0082 | Actinomycetota | Crossiella | 0.102765 | 2 | 0.073059 | 0.137787 | 0.197028 | 0.181773 | 0 |
| F_Otu0003 | Mortierellomycota | Mortierella | 7.22196 | 1 | 0.028447 | 0.106452 | 0.147431 | 0 | 0 |
| F_Otu0002 | Ascomycota | Fusarium | 6.855633 | 1 | 0.015386 | 0.139831 | 0.177028 | 0 | 0 |
| B_Otu0001 | Actinomycetota | Nocardioides | 1.033695 | 1 | 0.01452 | 0.5 | 0.583333 | 0 | 0 |
| F_Otu0021 | Ascomycota | Clonostachys | 0.972036 | 1 | 0.056526 | 0.089431 | 0.135874 | 0 | 0 |
| B_Otu0005 | Actinomycetota | Streptomyces | 0.866853 | 1 | 0.009233 | 0.092437 | 0.127843 | 0 | 0 |
| F_Otu0027 | Ascomycota | Chaetomium | 0.843277 | 1 | 0.091797 | 1 | 1 | 0 | 0 |
| B_Otu0008 | Pseudomonadota | Sphingomonas | 0.621426 | 1 | 0.09932 | 0.666667 | 0.75 | 0 | 0 |
| B_Otu0006 | Pseudomonadota | Microvirga | 0.487529 | 1 | 0.029232 | 1 | 1 | 0 | 0 |
| F_Otu0105 | Ascomycota | Sagenomella | 0.395041 | 1 | 0.059436 | 0.6 | 0.666667 | 0 | 0 |
| B_Otu0057 | Actinomycetota | Gaiella | 0.330964 | 1 | 0.025923 | 0.123364 | 0.172902 | 0 | 0 |
| F_Otu0039 | Ascomycota | Lophotrichus | 0.300134 | 1 | 0.059919 | 0.096633 | 0.131193 | 0 | 0 |
| B_Otu0034 | Gemmatimonadota | Gemmatimonas | 0.293485 | 1 | 0.033912 | 1 | 1 | 0 | 0 |
| F_Otu0091 | Ascomycota | Scytalidium | 0.252983 | 1 | 0.030776 | 0.132265 | 0.181218 | 0 | 0 |
| F_Otu0072 | Ascomycota | Pseudophloeospora | 0.245729 | 1 | 0.033912 | 1 | 1 | 0 | 0 |
| F_Otu0047 | Basidiomycota | Naganishia | 0.212784 | 1 | 0.020876 | 0.108553 | 0.141684 | 0 | 0 |
| B_Otu0080 | Pseudomonadota | Rhodoplanes | 0.199787 | 1 | 0.115016 | 0.134146 | 0.174287 | 0 | 0 |
| B_Otu0026 | Actinomycetota | Actinomycetospora | 0.186488 | 1 | 0.049641 | 0.116402 | 0.149015 | 0 | 0 |
| B_Otu0098 | Actinomycetota | Actinophytocola | 0.184675 | 1 | 0.016134 | 1 | 1 | 0 | 0 |
| F_Otu0250 | Ascomycota | Albifimbria | 0.182559 | 1 | 0.061331 | 0.08871 | 0.128803 | 0 | 0 |
| F_Otu0168 | Ascomycota | Sporormiella | 0.180745 | 1 | 0.209495 | 0.102167 | 0.139232 | 0 | 0 |
| B_Otu0078 | Acidobacteriota | Subgroup_10 | 0.179536 | 1 | 0.034392 | 0.6 | 0.666667 | 0 | 0 |
| B_Otu0192 | Bacteroidota | Chryseobacterium | 0.176212 | 1 | 0.091797 | 1 | 1 | 0 | 0 |
| B_Otu0086 | Pseudomonadota | TRA3-20_ge | 0.166842 | 1 | 0.029232 | 1 | 1 | 0 | 0 |
| F_Otu0064 | Ascomycota | Caliciopsis | 0.152334 | 1 | 0.111987 | 0.150342 | 0.201832 | 0 | 0 |
| B_Otu0054 | Actinomycetota | Pseudarthrobacter | 0.15052 | 1 | 0.01147 | 0.6 | 0.666667 | 0 | 0 |
| F_Otu0290 | Ascomycota | Auxarthron | 0.128758 | 1 | 0.091037 | 0.160584 | 0.211973 | 0 | 0 |
| B_Otu0077 | Verrucomicrobia | Pedosphaeraceae_ge | 0.125131 | 1 | 0.069634 | 0.666667 | 0.75 | 0 | 0 |
| B_Otu0035 | Actinomycetota | Geodermatophilus | 0.118784 | 1 | 0.016134 | 1 | 1 | 0 | 0 |
| F_Otu0206 | Ascomycota | Retroconis | 0.11818 | 1 | 0.295615 | 0.130952 | 0.17076 | 0 | 0 |
| F_Otu0209 | Ascomycota | Purpureocillium | 0.109112 | 1 | 0.097932 | 0.444444 | 0.541667 | 0 | 0 |
| B_Otu0090 | Actinomycetota | Conexibacter | 0.105183 | 1 | 0.22174 | 0.5 | 0.583333 | 0 | 0 |
| B_Otu0143 | Pseudomonadota | Nordella | 0.103369 | 1 | 0.191277 | 0.6 | 0.666667 | 0 | 0 |
| Pre-Plant KM Networks | | | | | | | | | |
| F_Otu0009 | Basidiomycota | Solicoccozyma | 2.374291 | 1 | 0.055347 | 0.666667 | 0.75 | 0 | 0 |
| B_Otu0003 | Actinomycetota | 67-14_ge | 1.970684 | 2 | 0.100812 | 0.110145 | 0.199349 | 0.01142 | 0 |
| F_Otu0005 | Ascomycota | Penicillium | 1.798386 | 2 | 0.168595 | 0.175926 | 0.255213 | 0.01142 | 0 |
| B_Otu0001 | Actinomycetota | Nocardioides | 1.757223 | 1 | 0.049023 | 0.35 | 0.445238 | 0 | 0 |
| F_Otu0030 | Ascomycota | Aspergillus | 1.320103 | 2 | 0.288225 | 0.75 | 0.833333 | 0.000617 | 0 |
| B_Otu0005 | Actinomycetota | Streptomyces | 1.30189 | 3 | 0.074957 | 0.5 | 0.654762 | 0.004321 | 0 |
| F_Otu0057 | Basidiomycota | Trechispora | 1.279305 | 4 | 0.530734 | 0.40625 | 0.553846 | 0.014815 | 0 |
| F_Otu0014 | Ascomycota | Preussia | 1.211552 | 1 | 0.236969 | 1 | 1 | 0 | 0 |
| B_Otu0022 | Actinomycetota | Rubrobacter | 0.969678 | 2 | 0.238082 | 0.342105 | 0.447436 | 0.00679 | 0 |
| F_Otu0027 | Ascomycota | Chaetomium | 0.918317 | 1 | 0.090068 | 0.125413 | 0.18237 | 0 | 0 |
| B_Otu0007 | Actinomycetota | Mycobacterium | 0.90921 | 3 | 0.166564 | 0.189055 | 0.278404 | 0.107099 | 0 |
| F_Otu0048 | Ascomycota | Neocosmospora | 0.815229 | 1 | 0.001125 | 1 | 1 | 0 | 0 |
| B_Otu0006 | Pseudomonadota | Microvirga | 0.79993 | 1 | 0.080141 | 0.240741 | 0.313553 | 0 | 0 |
| F_Otu0023 | Ascomycota | Pseudopyrenochaeta | 0.782081 | 2 | 0.13426 | 0.205405 | 0.273079 | 0.110185 | 0 |
| F_Otu0020 | Ascomycota | Paramyrothecium | 0.734362 | 1 | 0.011429 | 0.5 | 0.611111 | 0 | 0 |
| B_Otu0008 | Pseudomonadota | Sphingomonas | 0.632732 | 2 | 0.267626 | 0.131488 | 0.210294 | 0 | 1 |
| B_Otu0011 | Acidobacteriota | Bryobacter | 0.569349 | 2 | 0.006878 | 0.388889 | 0.540476 | 0.001852 | 0 |
| B_Otu0015 | Actinomycetota | Solirubrobacter | 0.512159 | 1 | 0.022243 | 1 | 1 | 0 | 0 |
| B_Otu0021 | Pseudomonadota | Skermanella | 0.498681 | 2 | 0.073521 | 0.325 | 0.428205 | 0.003704 | 0 |
| B_Otu0020 | Actinomycetota | Marmoricola | 0.476097 | 2 | 0.223651 | 0.130584 | 0.203715 | 0.01142 | 0 |
| B_Otu0018 | Actinomycetota | Pseudonocardia | 0.458248 | 1 | 0.072376 | 1 | 1 | 0 | 0 |
| F_Otu0156 | Ascomycota | Wardomycopsis | 0.453512 | 1 | 0.105189 | 1 | 1 | 0 | 0 |
| B_Otu0048 | Pseudomonadota | MND1 | 0.429835 | 2 | 0.131828 | 0.270833 | 0.379487 | 0.003704 | 0 |
| F_Otu0100 | Ascomycota | Gibellulopsis | 0.413079 | 1 | 0.009755 | 0.139194 | 0.182354 | 0 | 0 |
| F_Otu0090 | Ascomycota | Stachybotrys | 0.400329 | 1 | 0.073715 | 0.099476 | 0.157952 | 0 | 0 |
| B_Otu0019 | Actinomycetota | Blastococcus | 0.352975 | 1 | 0.03954 | 0.115854 | 0.165245 | 0 | 0 |
| F_Otu0099 | Ascomycota | Emericellopsis | 0.344232 | 1 | 0.029925 | 0.145038 | 0.186299 | 0 | 0 |
| F_Otu0188 | Basidiomycota | Rhizoctonia | 0.341682 | 2 | 0.240104 | 0.148438 | 0.23658 | 0.022222 | 0 |
| B_Otu0057 | Actinomycetota | Gaiella | 0.337675 | 1 | 0.031389 | 0.309524 | 0.376923 | 0 | 0 |
| F_Otu0065 | Ascomycota | Hormiactis | 0.323469 | 1 | 0.104913 | 1 | 1 | 0 | 0 |
| F_Otu0058 | Ascomycota | Clonostachys | 0.321283 | 2 | 0.082431 | 0.161017 | 0.22782 | 0.01142 | 0 |
| F_Otu0059 | Chytridiomycota | Rhizophlyctis | 0.30307 | 2 | 0.162874 | 1 | 1 | 0.000309 | 0 |
| F_Otu0035 | Ascomycota | Schizothecium | 0.292142 | 2 | 0.244534 | 0.208791 | 0.284419 | 0.111111 | 0 |
| F_Otu0004 | Ascomycota | Alternaria | 0.290685 | 4 | 0.165368 | 0.122581 | 0.250203 | 0.043519 | 0 |
| B_Otu0034 | Gemmatimonadota | Gemmatimonas | 0.2834 | 1 | 0.041049 | 0.12459 | 0.175791 | 0 | 0 |
| B_Otu0052 | Pseudomonadota | Haliangium | 0.282307 | 2 | 0.106344 | 0.160338 | 0.242232 | 0.080556 | 0 |
| F_Otu0209 | Ascomycota | Purpureocillium | 0.279393 | 1 | 0.197464 | 0.5 | 0.611111 | 0 | 0 |
| F_Otu0052 | Ascomycota | Metarhizium | 0.277207 | 1 | 0.027979 | 1 | 1 | 0 | 0 |
| F_Otu0221 | Ascomycota | Blastobotrys | 0.272472 | 2 | 0.060732 | 0.184466 | 0.278897 | 0.059259 | 0 |
| F_Otu0301 | Ascomycota | Acremonium | 0.266279 | 2 | 0.177424 | 0.168889 | 0.232937 | 0.01142 | 0 |
| B_Otu0091 | Actinomycetota | 0319-7L14_ge | 0.256808 | 1 | 0.204328 | 0.269231 | 0.378571 | 0 | 0 |
| B_Otu0042 | Pseudomonadota | Acidibacter | 0.254258 | 1 | 0.072376 | 1 | 1 | 0 | 0 |
| B_Otu0026 | Actinomycetota | Actinomycetospora | 0.252801 | 3 | 0.285273 | 0.149606 | 0.254124 | 0.032407 | 1 |
| B_Otu0046 | Pseudomonadota | 0319-6G20_ge | 0.24333 | 1 | 0.071273 | 0.116923 | 0.17314 | 0 | 0 |
| F_Otu0118 | Ascomycota | Sporormiella | 0.241509 | 1 | 0.236969 | 1 | 1 | 0 | 0 |
| B_Otu0098 | Actinomycetota | Actinophytocola | 0.237502 | 1 | 0.031093 | 0.099476 | 0.157952 | 0 | 0 |
| B_Otu0068 | Actinomycetota | IMCC26256_ge | 0.232038 | 2 | 0.171545 | 0.133803 | 0.231882 | 0.04784 | 0 |
| B_Otu0079 | Actinomycetota | Kribbella | 0.229124 | 2 | 0.097777 | 0.174312 | 0.251003 | 0.08642 | 0 |
| F_Otu0031 | Ascomycota | Cladosporium | 0.22621 | 1 | 0.104913 | 1 | 1 | 0 | 0 |
| B_Otu0054 | Actinomycetota | Pseudarthrobacter | 0.219653 | 2 | 0.214889 | 0.35 | 0.504762 | 0.001852 | 0 |
| F_Otu0081 | Ascomycota | Plenodomus | 0.208725 | 1 | 0.027979 | 1 | 1 | 0 | 0 |
| B_Otu0035 | Actinomycetota | Geodermatophilus | 0.202532 | 3 | 0.13052 | 0.170404 | 0.285316 | 0.070679 | 0 |
| B_Otu0053 | Pseudomonadota | SC-I-84_ge | 0.202532 | 1 | 0.001209 | 0.291667 | 0.397619 | 0 | 0 |
| B_Otu0056 | Pseudomonadota | BIrii41_ge | 0.195611 | 2 | 0.098353 | 0.131944 | 0.218724 | 0.009568 | 0 |
| B_Otu0080 | Pseudomonadota | Rhodoplanes | 0.193426 | 1 | 0.042397 | 0.160338 | 0.223474 | 0 | 0 |
| F_Otu0126 | Ascomycota | Periconia | 0.190876 | 2 | 0.025353 | 0.5 | 0.595238 | 0.003704 | 0 |
| B_Otu0102 | Pseudomonadota | CCD24_ge | 0.188326 | 4 | 0.23363 | 0.433333 | 0.564103 | 0.016358 | 0 |
| B_Otu0065 | Nitrospirotae | Nitrospirota | 0.186505 | 1 | 0.053708 | 0.216667 | 0.289927 | 0 | 0 |
| F_Otu0086 | Ascomycota | Setophoma | 0.182498 | 1 | 0.035821 | 0.125413 | 0.18237 | 0 | 0 |
| F_Otu0046 | Ascomycota | Beauveria | 0.182498 | 1 | 0.107527 | 0.666667 | 0.75 | 0 | 0 |
| F_Otu0271 | Ascomycota | Ophiocordyceps | 0.181769 | 1 | 0.105189 | 1 | 1 | 0 | 0 |
| B_Otu0086 | Pseudomonadota | TRA3-20_ge | 0.178855 | 1 | 0.138244 | 0.240741 | 0.313553 | 0 | 0 |
| B_Otu0074 | Acidobacteriota | Candidatus_Solibacter | 0.176305 | 3 | 0.21143 | 0.16309 | 0.268712 | 0.052778 | 0 |
| B_Otu0093 | Pseudomonadota | Reyranella | 0.169384 | 2 | 0.127947 | 0.121019 | 0.215115 | 0.001543 | 0 |
| B_Otu0094 | Pseudomonadota | Steroidobacter | 0.164649 | 3 | 0.242327 | 0.147287 | 0.250204 | 0.074383 | 0 |
| B_Otu0069 | Gemmatimonadota | Longimicrobiaceae_ge | 0.162099 | 2 | 0.198199 | 0.309524 | 0.421795 | 0.003704 | 0 |
| B_Otu0082 | Actinomycetota | Crossiella | 0.155542 | 3 | 0.189695 | 0.142857 | 0.237457 | 0.022531 | 0 |
| F_Otu0130 | Kickxellomycota | Ramicandelaber | 0.155542 | 3 | 0.157627 | 0.19 | 0.300827 | 0.083025 | 0 |
| F_Otu0071 | Ascomycota | Darksidea | 0.149349 | 2 | 0.10219 | 0.75 | 0.833333 | 0.000617 | 0 |
| B_Otu0099 | Acidobacteriota | Subgroup_7_ge | 0.144614 | 3 | 0.10249 | 0.131944 | 0.223452 | 0.01142 | 1 |
| B_Otu0122 | Actinomycetota | Actinoallomurus | 0.143157 | 2 | 0.092411 | 0.110145 | 0.199349 | 0.01142 | 0 |
| F_Otu0113 | Ascomycota | Spiromastix | 0.139878 | 3 | 0.23509 | 0.2 | 0.285338 | 0.117284 | 0 |
| F_Otu0192 | Basidiomycota | Atractiella | 0.133322 | 2 | 0.379216 | 0.309524 | 0.421795 | 0.003704 | 0 |
| B_Otu0136 | Actinomycetota | Actinomadura | 0.128222 | 1 | 0.001125 | 1 | 1 | 0 | 0 |
| F_Otu0108 | Ascomycota | Exophiala | 0.124944 | 4 | 0.359875 | 0.209945 | 0.328866 | 0.143519 | 0 |
| B_Otu0096 | Actinomycetota | Aeromicrobium | 0.121301 | 2 | 0.015649 | 0.4375 | 0.559524 | 0.003086 | 0 |
| B_Otu0063 | Bacteroidota | Flavisolibacter | 0.11948 | 1 | 0.022243 | 1 | 1 | 0 | 0 |
| B_Otu0090 | Actinomycetota | Conexibacter | 0.113287 | 2 | 0.149255 | 0.141791 | 0.219913 | 0.01142 | 0 |
| B_Otu0107 | Actinomycetota | Iamia | 0.102723 | 2 | 0.074177 | 0.433333 | 0.50641 | 0.012963 | 0 |
| F_Otu0203 | Ascomycota | Dichotomopilus | 0.101266 | 1 | 0.038204 | 0.25 | 0.317949 | 0 | 0 |
| F_Otu0105 | Ascomycota | Sagenomella | 0.100538 | 1 | 0.060564 | 0.150198 | 0.200291 | 0 | 0 |
| B_Otu0127 | Gemmatimonadota | S0134_terrestrial_group_ge | 0.100173 | 1 | 0.104488 | 0.295455 | 0.370513 | 0 | 0 |
| Pre-Plant KC Networks | | | | | | | | | |
| F_Otu0003 | Mortierellomycota | Mortierella | 7.670248 | 5 | 0.103296 | 0.666667 | 0.791667 | 0.007227 | 0 |
| B_Otu0080 | Pseudomonadota | Rhodoplanes | 0.174017 | 5 | 0.168539 | 0.252577 | 0.360706 | 0.139841 | 0 |
| B_Otu0102 | Pseudomonadota | CCD24_ge | 0.161767 | 5 | 0.393733 | 0.237864 | 0.358933 | 0.088101 | 1 |
| B_Otu0093 | Pseudomonadota | Reyranella | 0.161138 | 5 | 0.125296 | 0.233333 | 0.331625 | 0.083415 | 0 |
| B_Otu0140 | Pseudomonadota | Dongia | 0.103656 | 5 | 0.189395 | 0.202479 | 0.3173 | 0.029429 | 1 |
| F_Otu0128 | Ascomycota | Dactylonectria | 0.352745 | 4 | 0.113877 | 0.207627 | 0.315745 | 0.034528 | 1 |
| B_Otu0019 | Actinomycetota | Blastococcus | 0.269506 | 4 | 0.222509 | 0.189922 | 0.280831 | 0.094751 | 1 |
| B_Otu0026 | Actinomycetota | Actinomycetospora | 0.240294 | 4 | 0.24751 | 0.183521 | 0.270287 | 0.027702 | 0 |
| B_Otu0056 | Pseudomonadota | BIrii41_ge | 0.179043 | 4 | 0.194125 | 0.213974 | 0.325269 | 0.03247 | 0 |
| B_Otu0035 | Actinomycetota | Geodermatophilus | 0.174645 | 4 | 0.179513 | 0.216814 | 0.308673 | 0.15688 | 0 |
| F_Otu0119 | Basidiomycota | Schizophyllum | 0.164594 | 4 | 0.217643 | 0.221719 | 0.316075 | 0.035247 | 0 |
| B_Otu0094 | Pseudomonadota | Steroidobacter | 0.163337 | 4 | 0.36791 | 0.184906 | 0.289303 | 0.01935 | 0 |
| F_Otu0100 | Ascomycota | Gibellulopsis | 0.152657 | 4 | 0.444499 | 0.235577 | 0.322417 | 0.040941 | 0 |
| F_Otu0052 | Ascomycota | Metarhizium | 0.882963 | 3 | 0.090003 | 0.237864 | 0.303644 | 0.153568 | 1 |
| B_Otu0011 | Acidobacteriota | Bryobacter | 0.670624 | 3 | 0.261611 | 0.470588 | 0.614583 | 0.003613 | 1 |
| F_Otu0046 | Ascomycota | Beauveria | 0.562885 | 3 | 0.156107 | 0.246231 | 0.30277 | 0.09195 | 1 |
| F_Otu0069 | Ascomycota | Lectera | 0.489697 | 3 | 0.323278 | 0.247475 | 0.322352 | 0.080506 | 0 |
| B_Otu0048 | Pseudomonadota | MND1 | 0.345207 | 3 | 0.24201 | 0.204167 | 0.299953 | 0.016672 | 0 |
| B_Otu0020 | Actinomycetota | Marmoricola | 0.3317 | 3 | 0.182323 | 0.16388 | 0.242768 | 0.003714 | 1 |
| B_Otu0052 | Pseudomonadota | Haliangium | 0.277987 | 3 | 0.145003 | 0.221719 | 0.311508 | 0.039784 | 0 |
| B_Otu0053 | Pseudomonadota | SC-I-84_ge | 0.242493 | 3 | 0.112205 | 0.571429 | 0.666667 | 0.006323 | 0 |
| B_Otu0046 | Pseudomonadota | 0319-6G20_ge | 0.226787 | 3 | 0.224483 | 0.145833 | 0.224176 | 0.055405 | 0 |
| F_Otu0004 | Ascomycota | Alternaria | 0.149516 | 3 | 0.132622 | 0.242574 | 0.305539 | 0.071956 | 1 |
| B_Otu0069 | Gemmatimonadota | Longimicrobiaceae_ge | 0.142606 | 3 | 0.212458 | 0.181481 | 0.25328 | 0.020576 | 0 |
| F_Otu0232 | Ascomycota | Venturiocistella | 0.117477 | 3 | 0.150104 | 0.224771 | 0.311994 | 0.022992 | 0 |
| F_Otu0005 | Ascomycota | Penicillium | 3.007601 | 2 | 0.100977 | 0.421053 | 0.53125 | 0.002108 | 0 |
| F_Otu0019 | Basidiomycota | Solicoccozyma | 1.518721 | 2 | 0.152711 | 0.470588 | 0.5625 | 0.003613 | 0 |
| B_Otu0001 | Actinomycetota | Nocardioides | 1.037819 | 2 | 0.035919 | 0.162252 | 0.225761 | 0 | 1 |
| F_Otu0021 | Ascomycota | Clonostachys | 0.872283 | 2 | 0.240652 | 0.175 | 0.232517 | 0.028305 | 0 |
| B_Otu0008 | Pseudomonadota | Sphingomonas | 0.668112 | 2 | 0.18194 | 0.347826 | 0.483333 | 0 | 1 |
| B_Otu0031 | Bacillota | Sporosarcina | 0.648637 | 2 | 0.066544 | 0.470588 | 0.572917 | 0.002108 | 0 |
| F_Otu0129 | Ascomycota | Dichotomopilus | 0.560058 | 2 | 0.140574 | 0.245 | 0.301944 | 0.091499 | 0 |
| B_Otu0018 | Actinomycetota | Pseudonocardia | 0.493467 | 2 | 0.137059 | 0.128272 | 0.187469 | 0.014453 | 0 |
| F_Otu0056 | Ascomycota | Phoma | 0.458286 | 2 | 0.121785 | 0.182156 | 0.263793 | 0.001706 | 0 |
| B_Otu0034 | Gemmatimonadota | Gemmatimonas | 0.394522 | 2 | 0.06379 | 0.363636 | 0.489583 | 0.002108 | 0 |
| B_Otu0042 | Pseudomonadota | Acidibacter | 0.306571 | 2 | 0.294088 | 0.207627 | 0.276644 | 0.041554 | 0 |
| F_Otu0048 | Ascomycota | Neocosmospora | 0.236525 | 2 | 0.16956 | 0.157556 | 0.220708 | 0.000803 | 0 |
| F_Otu0130 | Kickxellomycota | Ramicandelaber | 0.233069 | 2 | 0.226396 | 0.208511 | 0.278855 | 0.007898 | 0 |
| F_Otu0035 | Ascomycota | Schizothecium | 0.211396 | 2 | 0.079643 | 1 | 1 | 0.000301 | 0 |
| B_Otu0068 | Actinomycetota | IMCC26256_ge | 0.195376 | 2 | 0.09397 | 0.199187 | 0.275803 | 0.009084 | 0 |
| B_Otu0082 | Actinomycetota | Crossiella | 0.183126 | 2 | 0.067802 | 0.347826 | 0.483333 | 0 | 1 |
| F_Otu0087 | Basidiomycota | Geminibasidium | 0.179043 | 2 | 0.179291 | 0.192913 | 0.257483 | 0.028305 | 0 |
| B_Otu0054 | Actinomycetota | Pseudarthrobacter | 0.175273 | 2 | 0.277811 | 0.4 | 0.510417 | 0.002108 | 0 |
| F_Otu0204 | Ascomycota | Humicola | 0.172132 | 2 | 0.249494 | 0.173759 | 0.243239 | 0.014453 | 0 |
| B_Otu0098 | Actinomycetota | Actinophytocola | 0.148574 | 2 | 0.237303 | 0.163333 | 0.215024 | 0.014453 | 0 |
| B_Otu0083 | Pseudomonadota | Candidatus_Alysiosphaera | 0.133183 | 2 | 0.020176 | 0.150307 | 0.199783 | 0.014453 | 0 |
| F_Otu0031 | Ascomycota | Cladosporium | 0.128157 | 2 | 0.136989 | 0.165541 | 0.238006 | 0.066245 | 0 |
| F_Otu0255 | Ascomycota | Periconia | 0.120618 | 2 | 0.185852 | 0.533333 | 0.604167 | 0.003613 | 0 |
| F_Otu0121 | Ascomycota | Knufia | 0.116221 | 2 | 0.042398 | 0.178832 | 0.262287 | 0.001807 | 0 |
| B_Otu0101 | Pseudomonadota | Ellin6055 | 0.10774 | 2 | 0.079068 | 0.128272 | 0.187469 | 0.014453 | 0 |
| F_Otu0002 | Ascomycota | Fusarium | 7.332265 | 1 | 0.082494 | 0.154088 | 0.195198 | 0 | 0 |
| F_Otu0023 | Ascomycota | Pseudopyrenochaeta | 1.579972 | 1 | 0.077943 | 1 | 1 | 0 | 0 |
| B_Otu0005 | Actinomycetota | Streptomyces | 1.019286 | 1 | 0.040503 | 0.113953 | 0.152991 | 0 | 0 |
| F_Otu0030 | Ascomycota | Aspergillus | 0.972798 | 1 | 0.044228 | 1 | 1 | 0 | 0 |
| B_Otu0022 | Actinomycetota | Rubrobacter | 0.824224 | 1 | 0.294257 | 0.15655 | 0.213401 | 0 | 0 |
| B_Otu0006 | Pseudomonadota | Microvirga | 0.697638 | 1 | 0.102211 | 1 | 1 | 0 | 0 |
| B_Otu0009 | Bacillota | Bacillus | 0.665599 | 1 | 0.058118 | 0.333333 | 0.410417 | 0 | 0 |
| F_Otu0024 | Ascomycota | Plectosphaerella | 0.663086 | 1 | 0.044228 | 1 | 1 | 0 | 0 |
| F_Otu0027 | Ascomycota | Chaetomium | 0.579533 | 1 | 0.065496 | 0.666667 | 0.75 | 0 | 0 |
| F_Otu0101 | Ascomycota | Coniothyrium | 0.459229 | 1 | 0.036964 | 0.421053 | 0.489583 | 0 | 0 |
| F_Otu0045 | Ascomycota | Pseudocoleophoma | 0.457344 | 1 | 0.014147 | 0.666667 | 0.75 | 0 | 0 |
| B_Otu0021 | Pseudomonadota | Skermanella | 0.392637 | 1 | 0.096322 | 0.307692 | 0.389583 | 0 | 0 |
| B_Otu0079 | Actinomycetota | Kribbella | 0.302802 | 1 | 0.022341 | 0.168966 | 0.227763 | 0 | 0 |
| F_Otu0041 | Ascomycota | Rodentomyces | 0.30186 | 1 | 0.037778 | 0.189922 | 0.238776 | 0 | 0 |
| F_Otu0173 | Ascomycota | Leptosphaerulina | 0.242807 | 1 | 0.24738 | 0.148485 | 0.191997 | 0 | 0 |
| B_Otu0065 | Nitrospirotae | Nitrospirota | 0.219563 | 1 | 0.122587 | 0.296296 | 0.379167 | 0 | 0 |
| F_Otu0124 | Basidiomycota | Entoloma | 0.211082 | 1 | 0.074153 | 0.182156 | 0.231586 | 0 | 0 |
| F_Otu0072 | Ascomycota | Pseudophloeospora | 0.2048 | 1 | 0.066095 | 1 | 1 | 0 | 0 |
| F_Otu0105 | Ascomycota | Sagenomella | 0.187524 | 1 | 0.013311 | 0.421053 | 0.489583 | 0 | 0 |
| F_Otu0064 | Ascomycota | Caliciopsis | 0.185325 | 1 | 0.036799 | 1 | 1 | 0 | 0 |
| F_Otu0049 | Ascomycota | Microdochium | 0.183126 | 1 | 0.063525 | 0.113953 | 0.152991 | 0 | 0 |
| F_Otu0073 | Ascomycota | Lophiotrema | 0.172132 | 1 | 0.13702 | 0.182156 | 0.231586 | 0 | 0 |
| F_Otu0248 | Ascomycota | Monosporascus | 0.16585 | 1 | 0.086996 | 1 | 1 | 0 | 0 |
| B_Otu0091 | Actinomycetota | 0319-7L14_ge | 0.159882 | 1 | 0.003113 | 0.275862 | 0.366667 | 0 | 0 |
| B_Otu0086 | Pseudomonadota | TRA3-20_ge | 0.15894 | 1 | 0.019804 | 0.131016 | 0.164204 | 0 | 0 |
| F_Otu0110 | Ascomycota | Chalara | 0.147946 | 1 | 0.077943 | 1 | 1 | 0 | 0 |
| B_Otu0111 | Pseudomonadota | Bradyrhizobium | 0.134125 | 1 | 0.036799 | 1 | 1 | 0 | 0 |
| B_Otu0063 | Bacteroidota | Flavisolibacter | 0.121246 | 1 | 0.042979 | 0.155556 | 0.202 | 0 | 0 |
| F_Otu0099 | Ascomycota | Emericellopsis | 0.119048 | 1 | 0.102211 | 1 | 1 | 0 | 0 |
| B_Otu0078 | Acidobacteriota | Subgroup_10 | 0.118105 | 1 | 0.013967 | 0.421053 | 0.489583 | 0 | 0 |
| F_Otu0086 | Ascomycota | Setophoma | 0.116221 | 1 | 0.06974 | 0.140805 | 0.174743 | 0 | 0 |
| B_Otu0122 | Actinomycetota | Actinoallomurus | 0.113394 | 1 | 0.086996 | 1 | 1 | 0 | 0 |
| B_Otu0084 | Pseudomonadota | Pseudolabrys | 0.112137 | 1 | 0.066095 | 1 | 1 | 0 | 0 |
| Langgewens KK Networks | | | | | | | | | |
| B_Otu0008 | Pseudomonadota | Sphingomonas | 0.672689 | 6 | 0.109692 | 0.195652 | 0.293768 | 0.149415 | 3 |
| F_Otu0038 | Basidiomycota | Filobasidium | 2.676423 | 5 | 0.524486 | 0.196185 | 0.282336 | 0.080227 | 3 |
| F_Otu0004 | Ascomycota | Alternaria | 2.117949 | 4 | 0.479544 | 0.205128 | 0.282573 | 0.16356 | 2 |
| F_Otu0051 | Basidiomycota | Cystofilobasidium | 0.523733 | 4 | 0.232516 | 0.178218 | 0.250154 | 0.0178 | 2 |
| B_Otu0011 | Acidobacteriota | Bryobacter | 0.519825 | 4 | 0.354194 | 0.181818 | 0.259827 | 0.098867 | 1 |
| B_Otu0070 | Pseudomonadota | Nitrosospira | 0.309637 | 4 | 0.192948 | 0.208092 | 0.292587 | 0.203143 | 1 |
| F_Otu0387 | Ascomycota | Tricellula | 0.17588 | 4 | 0.379474 | 0.201117 | 0.278831 | 0.19364 | 0 |
| B_Otu0116 | Bacteroidota | Pedobacter | 0.162852 | 4 | 0.204683 | 0.192 | 0.273821 | 0.206396 | 0 |
| B_Otu0078 | Acidobacteriota | Subgroup_10 | 0.124636 | 4 | 0.218235 | 0.167832 | 0.245751 | 0.103107 | 0 |
| B_Otu0100 | Bacteroidota | Segetibacter | 0.124636 | 4 | 0.283051 | 0.169811 | 0.243904 | 0.083151 | 1 |
| F_Otu0046 | Ascomycota | Beauveria | 0.114214 | 4 | 0.181827 | 0.192513 | 0.270305 | 0.160307 | 1 |
| B_Otu0082 | Actinomycetota | Crossiella | 0.111174 | 4 | 0.120885 | 0.19407 | 0.27327 | 0.080775 | 0 |
| B_Otu0129 | Pseudomonadota | Allorhizobium-Neorhizobium-Pararhizobium-Rhizobium | 0.103357 | 4 | 0.173148 | 0.207493 | 0.285197 | 0.187829 | 1 |
| F_Otu0015 | Basidiomycota | Vishniacozyma | 4.002258 | 3 | 0.244626 | 0.169014 | 0.237346 | 0.035088 | 1 |
| B_Otu0020 | Actinomycetota | Marmoricola | 0.515482 | 3 | 0.338471 | 0.363636 | 0.560417 | 0.002851 | 0 |
| B_Otu0034 | Gemmatimonadota | Gemmatimonas | 0.394754 | 3 | 0.211268 | 0.174334 | 0.241992 | 0.084284 | 1 |
| B_Otu0164 | Bacteroidota | Sphingobacterium | 0.272289 | 3 | 0.351348 | 0.143713 | 0.203672 | 0.045833 | 0 |
| B_Otu0077 | Verrucomicrobia | Pedosphaeraceae_ge | 0.24797 | 3 | 0.136041 | 0.17866 | 0.255047 | 0.160673 | 0 |
| B_Otu0063 | Bacteroidota | Flavisolibacter | 0.234073 | 3 | 0.154085 | 0.165517 | 0.231631 | 0.01557 | 1 |
| B_Otu0035 | Actinomycetota | Geodermatophilus | 0.228862 | 3 | 0.079404 | 0.11094 | 0.16983 | 0.088596 | 0 |
| B_Otu0046 | Pseudomonadota | 0319-6G20_ge | 0.208017 | 3 | 0.390227 | 0.145161 | 0.205544 | 0.030921 | 0 |
| F_Otu0415 | Ascomycota | Spegazzinia | 0.162418 | 3 | 0.213934 | 0.8 | 0.875 | 0.001096 | 0 |
| B_Otu0126 | Pseudomonadota | Pseudomonas | 0.160681 | 3 | 0.376544 | 0.156522 | 0.225558 | 0.101096 | 0 |
| B_Otu0068 | Actinomycetota | IMCC26256_ge | 0.152864 | 3 | 0.148189 | 0.11026 | 0.163153 | 0.030921 | 0 |
| B_Otu0083 | Pseudomonadota | Candidatus_Alysiosphaera | 0.147653 | 3 | 0.137584 | 0.15859 | 0.218811 | 0.01557 | 1 |
| F_Otu0253 | Ascomycota | Persiciospora | 0.132453 | 3 | 0.18618 | 0.165517 | 0.231631 | 0.01557 | 1 |
| B_Otu0145 | Bacteroidota | Ferruginibacter | 0.124202 | 3 | 0.295262 | 0.363636 | 0.560417 | 0.002851 | 0 |
| B_Otu0176 | Pseudomonadota | Brevundimonas | 0.115517 | 3 | 0.123341 | 0.165138 | 0.233139 | 0.074342 | 0 |
| B_Otu0148 | Pseudomonadota | Oligoflexus | 0.102488 | 3 | 0.229459 | 0.16476 | 0.228897 | 0.048319 | 0 |
| B_Otu0111 | Pseudomonadota | Bradyrhizobium | 0.102054 | 3 | 0.323362 | 0.138196 | 0.202358 | 0.074342 | 0 |
| F_Otu0002 | Ascomycota | Fusarium | 4.470839 | 2 | 0.035725 | 0.09219 | 0.137413 | 0.030702 | 0 |
| F_Otu0019 | Basidiomycota | Solicoccozyma | 1.169497 | 2 | 0.057394 | 0.142012 | 0.184001 | 0.01557 | 0 |
| B_Otu0009 | Bacillota | Bacillus | 0.63925 | 2 | 0.028853 | 0.142857 | 0.191913 | 0.030702 | 0 |
| B_Otu0026 | Actinomycetota | Actinomycetospora | 0.479003 | 2 | 0.196037 | 1 | 1 | 0.000219 | 0 |
| B_Otu0018 | Actinomycetota | Pseudonocardia | 0.456421 | 2 | 0.095188 | 0.17561 | 0.229151 | 0.059649 | 0 |
| B_Otu0059 | Bacteroidota | Flavobacterium | 0.394754 | 2 | 0.049501 | 0.171838 | 0.228665 | 0.026389 | 0 |
| F_Otu0076 | Ascomycota | Trichoderma | 0.364789 | 2 | 0.171361 | 0.125654 | 0.166074 | 0.01557 | 0 |
| F_Otu0049 | Ascomycota | Microdochium | 0.310939 | 2 | 0.024323 | 0.169412 | 0.222669 | 0.135965 | 0 |
| B_Otu0056 | Pseudomonadota | BIrii41_ge | 0.299214 | 2 | 0.070467 | 0.164384 | 0.217996 | 0.030702 | 0 |
| B_Otu0071 | Actinomycetota | Rhodococcus | 0.2775 | 2 | 0.018476 | 0.10084 | 0.150649 | 0.045395 | 0 |
| B_Otu0089 | Actinomycetota | Nakamurella | 0.260998 | 2 | 0.265823 | 0.421053 | 0.541667 | 0.003289 | 0 |
| F_Otu0108 | Ascomycota | Exophiala | 0.251444 | 2 | 0.081492 | 0.084706 | 0.124442 | 0.01557 | 0 |
| F_Otu0222 | Ascomycota | Microsphaeropsis | 0.247101 | 2 | 0.168371 | 0.100279 | 0.145325 | 0.01557 | 0 |
| B_Otu0048 | Pseudomonadota | MND1 | 0.209754 | 2 | 0.103883 | 0.11784 | 0.154144 | 0.01557 | 0 |
| F_Otu0309 | Ascomycota | Apiosordaria | 0.19716 | 2 | 0.252641 | 0.135084 | 0.181665 | 0.112281 | 0 |
| B_Otu0022 | Actinomycetota | Rubrobacter | 0.193686 | 2 | 0.013334 | 0.172662 | 0.232745 | 0 | 1 |
| F_Otu0114 | Chytridiomycota | Rhizophlyctis | 0.191514 | 2 | 0.060196 | 1 | 1 | 0.000219 | 0 |
| B_Otu0087 | Actinomycetota | Cellulomonas | 0.173275 | 2 | 0.075102 | 0.444444 | 0.541667 | 0.003509 | 0 |
| B_Otu0074 | Acidobacteriota | Candidatus_Solibacter | 0.152864 | 2 | 0.009038 | 0.122867 | 0.176772 | 0.045395 | 0 |
| B_Otu0069 | Gemmatimonadota | Longimicrobiaceae_ge | 0.148521 | 2 | 0.199427 | 0.126316 | 0.171218 | 0.01557 | 0 |
| B_Otu0101 | Pseudomonadota | Ellin6055 | 0.122031 | 2 | 0.10531 | 0.151579 | 0.19724 | 0.045395 | 0 |
| B_Otu0084 | Pseudomonadota | Pseudolabrys | 0.118556 | 2 | 0.084429 | 0.169412 | 0.233459 | 0.072076 | 0 |
| F_Otu0240 | Ascomycota | Dictyosporium | 0.114648 | 2 | 0.063388 | 0.122034 | 0.171672 | 0.099781 | 0 |
| F_Otu0352 | Ascomycota | Peyronellaea | 0.113345 | 2 | 0.20652 | 0.150628 | 0.19758 | 0.124342 | 0 |
| B_Otu0110 | Pseudomonadota | Amaricoccus | 0.10162 | 2 | 0.282422 | 0.421053 | 0.541667 | 0.003289 | 0 |
| F_Otu0113 | Ascomycota | Spiromastix | 0.100751 | 2 | 0.094033 | 0.132841 | 0.174164 | 0.030702 | 0 |
| F_Otu0376 | Ascomycota | Pyrenochaetopsis | 0.100317 | 2 | 0.191524 | 0.666667 | 0.75 | 0.000658 | 0 |
| F_Otu0005 | Ascomycota | Penicillium | 1.909932 | 1 | 0.172554 | 1 | 1 | 0 | 0 |
| B_Otu0001 | Actinomycetota | Nocardioides | 1.417466 | 1 | 0.025609 | 0.275862 | 0.389583 | 0 | 0 |
| F_Otu0039 | Ascomycota | Lophotrichus | 1.373605 | 1 | 0.150358 | 0.167832 | 0.209452 | 0 | 0 |
| B_Otu0003 | Actinomycetota | 67-14_ge | 0.966691 | 1 | 0.146263 | 1 | 1 | 0 | 0 |
| B_Otu0006 | Pseudomonadota | Microvirga | 0.831198 | 1 | 0.047118 | 0.275862 | 0.389583 | 0 | 0 |
| F_Otu0021 | Ascomycota | Clonostachys | 0.62709 | 1 | 0.172554 | 1 | 1 | 0 | 0 |
| B_Otu0015 | Actinomycetota | Solirubrobacter | 0.578017 | 1 | 0.146263 | 1 | 1 | 0 | 0 |
| B_Otu0021 | Pseudomonadota | Skermanella | 0.535893 | 1 | 0.024511 | 0.145455 | 0.186206 | 0 | 0 |
| B_Otu0005 | Actinomycetota | Streptomyces | 0.493768 | 1 | 0.089883 | 0.105572 | 0.130476 | 0 | 0 |
| B_Otu0007 | Actinomycetota | Mycobacterium | 0.488123 | 1 | 0.134264 | 0.666667 | 0.75 | 0 | 0 |
| F_Otu0200 | Ascomycota | Sporormiella | 0.452512 | 1 | 0.07907 | 0.099448 | 0.132763 | 0 | 0 |
| F_Otu0161 | Ascomycota | Pseudopithomyces | 0.366961 | 1 | 0.048348 | 0.078176 | 0.105946 | 0 | 0 |
| B_Otu0042 | Pseudomonadota | Acidibacter | 0.303557 | 1 | 0.106935 | 0.126984 | 0.16318 | 0 | 0 |
| B_Otu0053 | Pseudomonadota | SC-I-84_ge | 0.299648 | 1 | 0.242272 | 0.154176 | 0.19648 | 0 | 0 |
| F_Otu0306 | Ascomycota | Dinemasporium | 0.248404 | 1 | 0.074925 | 0.5 | 0.583333 | 0 | 0 |
| F_Otu0027 | Ascomycota | Chaetomium | 0.248404 | 1 | 0.032991 | 0.142012 | 0.181681 | 0 | 0 |
| B_Otu0057 | Actinomycetota | Gaiella | 0.22669 | 1 | 0.035446 | 0.666667 | 0.75 | 0 | 0 |
| B_Otu0095 | Pseudomonadota | Massilia | 0.220611 | 1 | 0.079637 | 0.275862 | 0.389583 | 0 | 0 |
| F_Otu0304 | Ascomycota | Blastobotrys | 0.220611 | 1 | 0.02177 | 0.5 | 0.583333 | 0 | 0 |
| B_Otu0051 | Actinomycetota | Modestobacter | 0.220176 | 1 | 0.061773 | 0.666667 | 0.75 | 0 | 0 |
| F_Otu0073 | Ascomycota | Lophiotrema | 0.19716 | 1 | 0.011323 | 0.124567 | 0.152476 | 0 | 0 |
| B_Otu0052 | Pseudomonadota | Haliangium | 0.188474 | 1 | 0.087558 | 0.135593 | 0.176663 | 0 | 0 |
| F_Otu0065 | Ascomycota | Hormiactis | 0.140704 | 1 | 0.140945 | 0.142292 | 0.180556 | 0 | 0 |
| B_Otu0080 | Pseudomonadota | Rhodoplanes | 0.138967 | 1 | 0.079138 | 0.121622 | 0.160568 | 0 | 0 |
| B_Otu0124 | Bacteroidota | Ohtaekwangia | 0.127242 | 1 | 0.061532 | 0.099448 | 0.132763 | 0 | 0 |
| B_Otu0120 | Bacteroidota | env.OPS_17_ge | 0.126808 | 1 | 0.030497 | 0.144 | 0.187025 | 0 | 0 |
| B_Otu0093 | Pseudomonadota | Reyranella | 0.123333 | 1 | 0.164133 | 0.126984 | 0.16318 | 0 | 0 |
| F_Otu0207 | Basidiomycota | Sporobolomyces | 0.123333 | 1 | 0.07233 | 0.151579 | 0.19111 | 0 | 0 |
| F_Otu0102 | Chytridiomycota | Clydaea | 0.123333 | 1 | 0.002602 | 0.161798 | 0.203396 | 0 | 0 |
| F_Otu0191 | Ascomycota | Thelebolus | 0.121162 | 1 | 0.074284 | 0.444444 | 0.541667 | 0 | 0 |
| B_Otu0065 | Nitrospirotae | Nitrospirota | 0.118556 | 1 | 0.039878 | 0.162896 | 0.205265 | 0 | 0 |
| B_Otu0115 | Pseudomonadota | Ellin6067 | 0.116819 | 1 | 0.008225 | 0.275862 | 0.389583 | 0 | 0 |
| B_Otu0094 | Pseudomonadota | Steroidobacter | 0.115082 | 1 | 0.02475 | 0.666667 | 0.75 | 0 | 0 |
| B_Otu0054 | Actinomycetota | Pseudarthrobacter | 0.114214 | 1 | 0.112976 | 0.091255 | 0.121672 | 0 | 0 |
| B_Otu0372 | Bacteroidota | Salinimicrobium | 0.112911 | 1 | 0.085049 | 0.112324 | 0.142243 | 0 | 0 |
| B_Otu0113 | Bacillota | Paenibacillus | 0.111174 | 1 | 0.179199 | 0.125874 | 0.161959 | 0 | 0 |
| B_Otu0123 | Pseudomonadota | Methylobacterium | 0.108134 | 1 | 0.162409 | 0.111801 | 0.139193 | 0 | 0 |
| B_Otu0169 | Pseudomonadota | Cellvibrio | 0.105528 | 1 | 0.053026 | 0.161435 | 0.205824 | 0 | 0 |
| B_Otu0135 | Pseudomonadota | Ramlibacter | 0.10466 | 1 | 0.060352 | 0.137143 | 0.172648 | 0 | 0 |
| F_Otu0266 | Ascomycota | Gelasinospora | 0.100751 | 1 | 0.175384 | 0.142292 | 0.180556 | 0 | 0 |
| Langgewens KM Networks | | | | | | | | | |
| F_Otu0109 | Ascomycota | Dokmaia | 0.251615 | 4 | 0.430043 | 1 | 1 | 0.001277 | 1 |
| B_Otu0096 | Actinomycetota | Aeromicrobium | 0.114682 | 4 | 0.432416 | 0.285714 | 0.42881 | 0.020557 | 0 |
| B_Otu0001 | Actinomycetota | Nocardioides | 2.065129 | 3 | 0.150533 | 0.473684 | 0.601852 | 0.003575 | 1 |
| F_Otu0015 | Basidiomycota | Vishniacozyma | 2.062989 | 3 | 0.411632 | 0.277778 | 0.394286 | 0.014556 | 0 |
| B_Otu0008 | Pseudomonadota | Sphingomonas | 0.671402 | 3 | 0.303911 | 0.204082 | 0.332401 | 0.009448 | 0 |
| B_Otu0059 | Bacteroidota | Flavobacterium | 0.541315 | 3 | 0.119167 | 0.327869 | 0.435 | 0.027324 | 0 |
| B_Otu0034 | Gemmatimonadota | Gemmatimonas | 0.359023 | 3 | 0.242365 | 0.295455 | 0.478297 | 0.010215 | 0 |
| B_Otu0056 | Pseudomonadota | BIrii41_ge | 0.321366 | 3 | 0.093085 | 0.5 | 0.6 | 0.008427 | 0 |
| F_Otu0023 | Ascomycota | Pseudopyrenochaeta | 0.24648 | 3 | 0.078618 | 0.45 | 0.583333 | 0.002043 | 1 |
| B_Otu0070 | Pseudomonadota | Nitrosospira | 0.240062 | 3 | 0.270745 | 0.4 | 0.541667 | 0.004341 | 0 |
| B_Otu0057 | Actinomycetota | Gaiella | 0.212247 | 3 | 0.150554 | 0.529412 | 0.62963 | 0.005107 | 1 |
| B_Otu0164 | Bacteroidota | Sphingobacterium | 0.167744 | 3 | 0.303621 | 0.333333 | 0.43 | 0.025919 | 0 |
| B_Otu0126 | Pseudomonadota | Pseudomonas | 0.127091 | 3 | 0.143502 | 0.277778 | 0.400119 | 0.014556 | 0 |
| F_Otu0126 | Ascomycota | Periconia | 0.126664 | 3 | 0.099549 | 1 | 1 | 0.000766 | 0 |
| B_Otu0124 | Bacteroidota | Ohtaekwangia | 0.121101 | 3 | 0.175102 | 0.357143 | 0.511667 | 0.004341 | 0 |
| B_Otu0093 | Pseudomonadota | Reyranella | 0.120673 | 3 | 0.132827 | 0.229885 | 0.35381 | 0.009448 | 0 |
| B_Otu0181 | Pseudomonadota | Luteimonas | 0.110831 | 3 | 0.155868 | 0.31746 | 0.425833 | 0.01762 | 0 |
| F_Otu0245 | Ascomycota | Coniochaeta | 0.109547 | 3 | 0.175327 | 0.325 | 0.481502 | 0.012002 | 0 |
| F_Otu0064 | Ascomycota | Caliciopsis | 0.100988 | 3 | 0.310626 | 0.473684 | 0.601852 | 0.005107 | 0 |
| F_Otu0004 | Ascomycota | Alternaria | 2.840943 | 2 | 0.196437 | 0.240964 | 0.34381 | 0.002043 | 0 |
| B_Otu0003 | Actinomycetota | 67-14_ge | 1.040267 | 2 | 0.094612 | 0.36 | 0.485185 | 0.002043 | 0 |
| B_Otu0006 | Pseudomonadota | Microvirga | 0.931576 | 2 | 0.059757 | 0.4 | 0.5 | 0.004086 | 0 |
| F_Otu0038 | Basidiomycota | Filobasidium | 0.90205 | 2 | 0.342482 | 0.229885 | 0.330476 | 0.009193 | 0 |
| F_Otu0039 | Ascomycota | Lophotrichus | 0.709059 | 2 | 0.123041 | 0.434783 | 0.525 | 0.005363 | 0 |
| B_Otu0007 | Actinomycetota | Mycobacterium | 0.568702 | 2 | 0.258923 | 1 | 1 | 0.000255 | 0 |
| B_Otu0021 | Pseudomonadota | Skermanella | 0.504515 | 2 | 0.125885 | 0.454545 | 0.616667 | 0.001021 | 0 |
| F_Otu0090 | Ascomycota | Stachybotrys | 0.421926 | 2 | 0.084969 | 0.309524 | 0.411538 | 0.010215 | 0 |
| F_Otu0049 | Ascomycota | Microdochium | 0.412512 | 2 | 0.225907 | 0.36 | 0.485185 | 0.002043 | 0 |
| B_Otu0011 | Acidobacteriota | Bryobacter | 0.354743 | 2 | 0.163805 | 0.240964 | 0.356012 | 0.013023 | 0 |
| F_Otu0051 | Basidiomycota | Cystofilobasidium | 0.321794 | 2 | 0.215937 | 0.192308 | 0.285456 | 0.004852 | 0 |
| F_Otu0087 | Basidiomycota | Geminibasidium | 0.272155 | 2 | 0.110346 | 0.282609 | 0.396154 | 0.009193 | 0 |
| B_Otu0089 | Actinomycetota | Nakamurella | 0.239634 | 2 | 0.314537 | 0.216667 | 0.357875 | 0.005618 | 0 |
| B_Otu0071 | Actinomycetota | Rhodococcus | 0.219094 | 2 | 0.114337 | 0.25 | 0.37967 | 0.007661 | 0 |
| B_Otu0048 | Pseudomonadota | MND1 | 0.208396 | 2 | 0.347652 | 0.185714 | 0.323687 | 0.003064 | 0 |
| F_Otu0036 | Ascomycota | Stemphylium | 0.180581 | 2 | 0.058789 | 0.25 | 0.33631 | 0.004852 | 0 |
| F_Otu0101 | Ascomycota | Coniothyrium | 0.173307 | 2 | 0.212242 | 0.240741 | 0.38428 | 0.003064 | 0 |
| B_Otu0078 | Acidobacteriota | Subgroup_10 | 0.147204 | 2 | 0.079872 | 0.454545 | 0.616667 | 0.001021 | 0 |
| B_Otu0110 | Pseudomonadota | Amaricoccus | 0.145064 | 2 | 0.237194 | 1 | 1 | 0.000255 | 0 |
| B_Otu0116 | Bacteroidota | Pedobacter | 0.144208 | 2 | 0.438414 | 0.298507 | 0.38 | 0.008427 | 0 |
| B_Otu0077 | Verrucomicrobia | Pedosphaeraceae_ge | 0.139073 | 2 | 0.264029 | 1 | 1 | 0.000255 | 0 |
| B_Otu0068 | Actinomycetota | IMCC26256_ge | 0.138217 | 2 | 0.06049 | 0.3125 | 0.431667 | 0.002298 | 0 |
| F_Otu0373 | Chytridiomycota | Operculomyces | 0.120673 | 2 | 0.089417 | 0.666667 | 0.75 | 0 | 1 |
| F_Otu0056 | Ascomycota | Phoma | 0.118533 | 2 | 0.13715 | 0.555556 | 0.666667 | 0.001532 | 0 |
| F_Otu0348 | Ascomycota | Thelonectria | 0.117249 | 2 | 0.146102 | 0.555556 | 0.666667 | 0.001532 | 0 |
| F_Otu0246 | Ascomycota | Niesslia | 0.113398 | 2 | 0.251173 | 0.666667 | 0.75 | 0 | 1 |
| B_Otu0090 | Actinomycetota | Conexibacter | 0.109547 | 2 | 0.07813 | 0.240741 | 0.38428 | 0.003064 | 0 |
| B_Otu0082 | Actinomycetota | Crossiella | 0.100561 | 2 | 0.034518 | 0.325 | 0.432051 | 0.010725 | 0 |
| F_Otu0002 | Ascomycota | Fusarium | 7.859985 | 1 | 0.03344 | 0.20202 | 0.257679 | 0 | 0 |
| F_Otu0018 | Mortierellomycota | Mortierella | 3.037357 | 1 | 0.174049 | 0.162602 | 0.224544 | 0 | 0 |
| F_Otu0005 | Ascomycota | Penicillium | 1.50199 | 1 | 0.079389 | 1 | 1 | 0 | 0 |
| F_Otu0014 | Ascomycota | Preussia | 1.190466 | 1 | 0.096536 | 1 | 1 | 0 | 0 |
| F_Otu0019 | Basidiomycota | Solicoccozyma | 0.979075 | 1 | 0.102454 | 0.158537 | 0.25116 | 0 | 0 |
| F_Otu0081 | Ascomycota | Plenodomus | 0.859686 | 1 | 0.074846 | 0.188679 | 0.259266 | 0 | 0 |
| F_Otu0048 | Ascomycota | Neocosmospora | 0.685096 | 1 | 0.189321 | 1 | 1 | 0 | 0 |
| B_Otu0005 | Actinomycetota | Streptomyces | 0.55672 | 1 | 0.024468 | 0.321429 | 0.401852 | 0 | 0 |
| B_Otu0026 | Actinomycetota | Actinomycetospora | 0.555865 | 1 | 0.019004 | 0.19697 | 0.289408 | 0 | 0 |
| F_Otu0095 | Ascomycota | Aspergillus | 0.520775 | 1 | 0.028304 | 0.666667 | 0.75 | 0 | 0 |
| B_Otu0015 | Actinomycetota | Solirubrobacter | 0.490821 | 1 | 0.030779 | 0.188679 | 0.259266 | 0 | 0 |
| B_Otu0018 | Actinomycetota | Pseudonocardia | 0.484402 | 1 | 0.232478 | 0.666667 | 0.75 | 0 | 0 |
| F_Otu0168 | Ascomycota | Sporormiella | 0.403526 | 1 | 0.096536 | 1 | 1 | 0 | 0 |
| F_Otu0027 | Ascomycota | Chaetomium | 0.375284 | 1 | 0.000689 | 0.272727 | 0.359259 | 0 | 0 |
| F_Otu0035 | Ascomycota | Schizothecium | 0.366725 | 1 | 0.019389 | 1 | 1 | 0 | 0 |
| F_Otu0065 | Ascomycota | Hormiactis | 0.359451 | 1 | 0.058844 | 0.27027 | 0.36 | 0 | 0 |
| F_Otu0069 | Ascomycota | Lectera | 0.342762 | 1 | 0.07972 | 0.571429 | 0.625 | 0 | 0 |
| B_Otu0022 | Actinomycetota | Rubrobacter | 0.320938 | 1 | 0.055941 | 0.333333 | 0.456667 | 0 | 0 |
| F_Otu0076 | Ascomycota | Trichoderma | 0.319226 | 1 | 0.037174 | 0.243902 | 0.326667 | 0 | 0 |
| F_Otu0268 | Ascomycota | Acremonium | 0.319226 | 1 | 0.007002 | 0.6 | 0.666667 | 0 | 0 |
| F_Otu0024 | Ascomycota | Plectosphaerella | 0.300826 | 1 | 0.000976 | 0.333333 | 0.456667 | 0 | 0 |
| F_Otu0108 | Ascomycota | Exophiala | 0.27943 | 1 | 0.012081 | 0.17094 | 0.245536 | 0 | 0 |
| B_Otu0051 | Actinomycetota | Modestobacter | 0.274723 | 1 | 0.014462 | 1 | 1 | 0 | 0 |
| B_Otu0042 | Pseudomonadota | Acidibacter | 0.243485 | 1 | 0.154677 | 0.17094 | 0.245536 | 0 | 0 |
| F_Otu0407 | Ascomycota | Truncatella | 0.240062 | 1 | 0.20889 | 0.666667 | 0.75 | 0 | 0 |
| B_Otu0046 | Pseudomonadota | 0319-6G20_ge | 0.228508 | 1 | 0.079389 | 1 | 1 | 0 | 0 |
| B_Otu0083 | Pseudomonadota | Candidatus_Alysiosphaera | 0.218666 | 1 | 0.03155 | 0.666667 | 0.75 | 0 | 0 |
| B_Otu0087 | Actinomycetota | Cellulomonas | 0.216954 | 1 | 0.037249 | 0.272727 | 0.359259 | 0 | 0 |
| B_Otu0063 | Bacteroidota | Flavisolibacter | 0.201549 | 1 | 0.014462 | 1 | 1 | 0 | 0 |
| F_Otu0190 | Ascomycota | Zopfiella | 0.183577 | 1 | 0.118555 | 0.25 | 0.337271 | 0 | 0 |
| B_Otu0052 | Pseudomonadota | Haliangium | 0.177586 | 1 | 0.009419 | 0.333333 | 0.411111 | 0 | 0 |
| B_Otu0107 | Actinomycetota | Iamia | 0.173307 | 1 | 0.036718 | 0.27027 | 0.36 | 0 | 0 |
| B_Otu0053 | Pseudomonadota | SC-I-84_ge | 0.169883 | 1 | 0.202662 | 0.666667 | 0.75 | 0 | 0 |
| F_Otu0046 | Ascomycota | Beauveria | 0.165604 | 1 | 0.031764 | 0.294118 | 0.376667 | 0 | 0 |
| B_Otu0069 | Gemmatimonadota | Longimicrobiaceae_ge | 0.157474 | 1 | 0.225839 | 0.294118 | 0.376667 | 0 | 0 |
| F_Otu0389 | Basidiomycota | Coniophora | 0.137361 | 1 | 0.070878 | 0.19697 | 0.289408 | 0 | 0 |
| B_Otu0101 | Pseudomonadota | Ellin6055 | 0.123668 | 1 | 0.176181 | 0.571429 | 0.625 | 0 | 0 |
| B_Otu0145 | Bacteroidota | Ferruginibacter | 0.118105 | 1 | 0.019389 | 1 | 1 | 0 | 0 |
| B_Otu0102 | Pseudomonadota | CCD24_ge | 0.11297 | 1 | 0.189321 | 1 | 1 | 0 | 0 |
| B_Otu0123 | Pseudomonadota | Methylobacterium | 0.110403 | 1 | 0.080544 | 0.6 | 0.666667 | 0 | 0 |
| F_Otu0071 | Ascomycota | Darksidea | 0.108691 | 1 | 0.012002 | 0.6 | 0.666667 | 0 | 0 |
| F_Otu0066 | Ascomycota | Fusariella | 0.105696 | 1 | 0.052186 | 0.224719 | 0.297679 | 0 | 0 |
| B_Otu0080 | Pseudomonadota | Rhodoplanes | 0.102272 | 1 | 0.056261 | 0.666667 | 0.75 | 0 | 0 |
| Langgewens KC Networks | | | | | | | | | |
| B_Otu0078 | Acidobacteriota | Subgroup_10 | 0.160743 | 6 | 0.498152 | 0.157407 | 0.281025 | 0.250171 | 0 |
| B_Otu0011 | Acidobacteriota | Bryobacter | 0.562408 | 4 | 0.301424 | 0.114094 | 0.227698 | 0.049897 | 1 |
| B_Otu0169 | Pseudomonadota | Cellvibrio | 0.100995 | 4 | 0.156165 | 0.126238 | 0.254918 | 0.171907 | 1 |
| F_Otu0056 | Ascomycota | Phoma | 2.287025 | 3 | 0.089087 | 0.093066 | 0.197019 | 0.050581 | 0 |
| F_Otu0004 | Ascomycota | Alternaria | 1.491404 | 3 | 0.306909 | 0.156442 | 0.245385 | 0.228298 | 0 |
| B_Otu0006 | Pseudomonadota | Microvirga | 1.152186 | 3 | 0.15745 | 0.105372 | 0.20847 | 0.065277 | 0 |
| F_Otu0038 | Basidiomycota | Filobasidium | 1.058515 | 3 | 0.459514 | 0.143258 | 0.24304 | 0.207792 | 0 |
| F_Otu0051 | Basidiomycota | Cystofilobasidium | 0.494565 | 3 | 0.250062 | 0.134921 | 0.244166 | 0.181476 | 0 |
| F_Otu0041 | Ascomycota | Rodentomyces | 0.48223 | 3 | 0.060826 | 0.092727 | 0.192117 | 0.019822 | 0 |
| B_Otu0015 | Actinomycetota | Solirubrobacter | 0.466425 | 3 | 0.044728 | 0.1 | 0.200923 | 0.074504 | 0 |
| B_Otu0075 | Pseudomonadota | Devosia | 0.341917 | 3 | 0.199587 | 0.149123 | 0.242204 | 0.196514 | 0 |
| F_Otu0101 | Ascomycota | Coniothyrium | 0.2999 | 3 | 0.037816 | 0.099609 | 0.198309 | 0.045796 | 0 |
| F_Otu0020 | Ascomycota | Paramyrothecium | 0.287179 | 3 | 0.05982 | 1 | 1 | 0.000684 | 1 |
| B_Otu0070 | Pseudomonadota | Nitrosospira | 0.141084 | 3 | 0.233304 | 0.16242 | 0.252498 | 0.24607 | 0 |
| B_Otu0101 | Pseudomonadota | Ellin6055 | 0.139928 | 3 | 0.222401 | 0.115385 | 0.228024 | 0.093985 | 0 |
| F_Otu0277 | Ascomycota | Monosporascus | 0.129134 | 3 | 0.163051 | 0.160377 | 0.247574 | 0.227614 | 0 |
| B_Otu0068 | Actinomycetota | IMCC26256_ge | 0.114872 | 3 | 0.040273 | 0.108051 | 0.211077 | 0.14149 | 0 |
| B_Otu0074 | Acidobacteriota | Candidatus_Solibacter | 0.106006 | 3 | 0.115091 | 0.096226 | 0.185734 | 0.033835 | 0 |
| F_Otu0021 | Ascomycota | Clonostachys | 1.956673 | 2 | 0.044578 | 0.14011 | 0.214098 | 0.177033 | 0 |
| F_Otu0015 | Basidiomycota | Vishniacozyma | 1.844499 | 2 | 0.402407 | 0.15 | 0.232112 | 0.203008 | 0 |
| F_Otu0005 | Ascomycota | Penicillium | 1.680672 | 2 | 0.069578 | 0.123188 | 0.196279 | 0.159945 | 0 |
| F_Otu0014 | Ascomycota | Preussia | 1.012644 | 2 | 0.052495 | 1 | 1 | 0.000342 | 0 |
| F_Otu0122 | Ascomycota | Oedohysterium | 0.933236 | 2 | 0.064287 | 0.75 | 0.833333 | 0.000171 | 0 |
| B_Otu0003 | Actinomycetota | 67-14_ge | 0.880426 | 2 | 0.029467 | 0.115385 | 0.19708 | 0.150376 | 0 |
| B_Otu0019 | Actinomycetota | Blastococcus | 0.781744 | 2 | 0.194687 | 0.75 | 0.833333 | 0.000171 | 0 |
| B_Otu0020 | Actinomycetota | Marmoricola | 0.679978 | 2 | 0.138904 | 0.454545 | 0.616667 | 0.001367 | 0 |
| B_Otu0008 | Pseudomonadota | Sphingomonas | 0.659934 | 2 | 0.329982 | 0.10303 | 0.18298 | 0.017088 | 0 |
| B_Otu0007 | Actinomycetota | Mycobacterium | 0.587464 | 2 | 0.136943 | 0.079439 | 0.14711 | 0.017088 | 0 |
| B_Otu0035 | Actinomycetota | Geodermatophilus | 0.558939 | 2 | 0.177608 | 0.75 | 0.833333 | 0.000171 | 0 |
| B_Otu0031 | Bacillota | Sporosarcina | 0.425565 | 2 | 0.031116 | 0.454545 | 0.616667 | 0.001367 | 0 |
| B_Otu0051 | Actinomycetota | Modestobacter | 0.32881 | 2 | 0.308008 | 0.75 | 0.833333 | 0.000171 | 0 |
| B_Otu0059 | Bacteroidota | Flavobacterium | 0.314162 | 2 | 0.088597 | 0.126238 | 0.198788 | 0.017088 | 0 |
| F_Otu0173 | Ascomycota | Leptosphaerulina | 0.287565 | 2 | 0.108721 | 0.75 | 0.833333 | 0 | 1 |
| B_Otu0042 | Pseudomonadota | Acidibacter | 0.264436 | 2 | 0.101529 | 0.137097 | 0.213034 | 0.017088 | 0 |
| B_Otu0087 | Actinomycetota | Cellulomonas | 0.193123 | 2 | 0.098141 | 0.113333 | 0.204822 | 0 | 1 |
| F_Otu0228 | Ascomycota | Sporormia | 0.169995 | 2 | 0.202159 | 0.555556 | 0.666667 | 0.002051 | 0 |
| F_Otu0157 | Ascomycota | Latorua | 0.168067 | 2 | 0.264006 | 1 | 1 | 0.000342 | 0 |
| B_Otu0077 | Verrucomicrobia | Pedosphaeraceae_ge | 0.158816 | 2 | 0.1829 | 0.160377 | 0.245909 | 0.220096 | 0 |
| B_Otu0116 | Bacteroidota | Pedobacter | 0.150335 | 2 | 0.094426 | 0.125 | 0.181755 | 0.017088 | 0 |
| F_Otu0059 | Chytridiomycota | Rhizophlyctis | 0.144939 | 2 | 0.11349 | 0.136364 | 0.201385 | 0.017088 | 0 |
| B_Otu0160 | Actinomycetota | Williamsia | 0.141855 | 2 | 0.084517 | 0.131443 | 0.201732 | 0.168831 | 0 |
| B_Otu0096 | Actinomycetota | Aeromicrobium | 0.125665 | 2 | 0.021549 | 0.141667 | 0.210538 | 0.033493 | 0 |
| B_Otu0069 | Gemmatimonadota | Longimicrobiaceae_ge | 0.125665 | 2 | 0.069053 | 0.085859 | 0.167468 | 0.033493 | 0 |
| F_Otu0423 | Mortierellomycota | Gamsiella | 0.113715 | 2 | 0.130949 | 0.555556 | 0.666667 | 0.002051 | 0 |
| B_Otu0093 | Pseudomonadota | Reyranella | 0.10138 | 2 | 0.090868 | 0.75 | 0.833333 | 0 | 1 |
| F_Otu0002 | Ascomycota | Fusarium | 4.936397 | 1 | 0.022956 | 0.130102 | 0.184235 | 0 | 0 |
| B_Otu0001 | Actinomycetota | Nocardioides | 1.6136 | 1 | 0.156778 | 0.093578 | 0.147219 | 0 | 0 |
| F_Otu0046 | Ascomycota | Beauveria | 1.258191 | 1 | 0.057323 | 1 | 1 | 0 | 0 |
| F_Otu0048 | Ascomycota | Neocosmospora | 1.010331 | 1 | 0.030345 | 0.6 | 0.666667 | 0 | 0 |
| F_Otu0134 | Ascomycota | Torula | 0.843034 | 1 | 0.166941 | 0.666667 | 0.75 | 0 | 0 |
| F_Otu0024 | Ascomycota | Plectosphaerella | 0.557397 | 1 | 0.055349 | 0.136364 | 0.199962 | 0 | 0 |
| B_Otu0005 | Actinomycetota | Streptomyces | 0.519621 | 1 | 0.128503 | 0.073699 | 0.121152 | 0 | 0 |
| B_Otu0026 | Actinomycetota | Actinomycetospora | 0.515766 | 1 | 0.016903 | 1 | 1 | 0 | 0 |
| F_Otu0121 | Ascomycota | Knufia | 0.443297 | 1 | 0.014984 | 0.102616 | 0.169908 | 0 | 0 |
| F_Otu0049 | Ascomycota | Microdochium | 0.429419 | 1 | 0.000133 | 0.120283 | 0.163208 | 0 | 0 |
| B_Otu0009 | Bacillota | Bacillus | 0.425179 | 1 | 0.087862 | 0.111354 | 0.149775 | 0 | 0 |
| F_Otu0091 | Ascomycota | Scytalidium | 0.368514 | 1 | 0.011497 | 0.090747 | 0.15192 | 0 | 0 |
| F_Otu0019 | Basidiomycota | Solicoccozyma | 0.360034 | 1 | 0.097065 | 0.666667 | 0.75 | 0 | 0 |
| F_Otu0069 | Ascomycota | Lectera | 0.323028 | 1 | 0.009705 | 0.087931 | 0.14493 | 0 | 0 |
| F_Otu0118 | Ascomycota | Sporormiella | 0.314933 | 1 | 0.015162 | 0.120853 | 0.168548 | 0 | 0 |
| B_Otu0056 | Pseudomonadota | BIrii41_ge | 0.265978 | 1 | 0.068546 | 0.136364 | 0.199962 | 0 | 0 |
| F_Otu0282 | Basidiomycota | Coprinopsis | 0.232056 | 1 | 0.030105 | 0.666667 | 0.75 | 0 | 0 |
| B_Otu0052 | Pseudomonadota | Haliangium | 0.209313 | 1 | 0.004752 | 0.333333 | 0.456667 | 0 | 0 |
| B_Otu0046 | Pseudomonadota | 0319-6G20_ge | 0.175006 | 1 | 0.034502 | 0.085 | 0.146879 | 0 | 0 |
| B_Otu0083 | Pseudomonadota | Candidatus_Alysiosphaera | 0.166911 | 1 | 0.085505 | 0.103659 | 0.172523 | 0 | 0 |
| B_Otu0109 | Actinomycetota | Jatrophihabitans | 0.151877 | 1 | 0.035971 | 0.087931 | 0.14493 | 0 | 0 |
| B_Otu0107 | Actinomycetota | Iamia | 0.14995 | 1 | 0.015404 | 1 | 1 | 0 | 0 |
| F_Otu0413 | Ascomycota | Pseudorobillarda | 0.149179 | 1 | 0.02239 | 0.666667 | 0.75 | 0 | 0 |
| B_Otu0063 | Bacteroidota | Flavisolibacter | 0.143397 | 1 | 0.001162 | 0.091071 | 0.153554 | 0 | 0 |
| B_Otu0347 | Pseudomonadota | Janthinobacterium | 0.141084 | 1 | 0.015404 | 1 | 1 | 0 | 0 |
| B_Otu0084 | Pseudomonadota | Pseudolabrys | 0.128749 | 1 | 0.103119 | 0.119159 | 0.184983 | 0 | 0 |
| B_Otu0095 | Pseudomonadota | Massilia | 0.126821 | 1 | 0.010395 | 0.112335 | 0.160707 | 0 | 0 |
| B_Otu0082 | Actinomycetota | Crossiella | 0.125279 | 1 | 0.052807 | 0.095506 | 0.15959 | 0 | 0 |
| B_Otu0099 | Acidobacteriota | Subgroup_7_ge | 0.112173 | 1 | 0.115284 | 0.136364 | 0.199962 | 0 | 0 |
| F_Otu0108 | Ascomycota | Exophiala | 0.111788 | 1 | 0.057323 | 1 | 1 | 0 | 0 |
| F_Otu0109 | Ascomycota | Dokmaia | 0.111402 | 1 | 0.016903 | 1 | 1 | 0 | 0 |
| B_Otu0115 | Pseudomonadota | Ellin6067 | 0.107548 | 1 | 0.079693 | 0.138587 | 0.189829 | 0 | 0 |
| B_Otu0120 | Bacteroidota | env.OPS_17_ge | 0.102151 | 1 | 0.04133 | 0.333333 | 0.456667 | 0 | 0 |
| Tygerhoek KK Networks | | | | | | | | | |
| F_Otu0062 | Ascomycota | Articulospora | 0.476386 | 5 | 0.335939 | 0.23125 | 0.347683 | 0.058062 | 0 |
| F_Otu0128 | Ascomycota | Dactylonectria | 0.207416 | 5 | 0.211027 | 0.217647 | 0.337881 | 0.053493 | 0 |
| B_Otu0078 | Acidobacteriota | Subgroup_10 | 0.139148 | 5 | 0.065494 | 0.196429 | 0.337345 | 0.080525 | 1 |
| B_Otu0129 | Pseudomonadota | Allorhizobium-Neorhizobium-Pararhizobium-Rhizobium | 0.101843 | 5 | 0.325803 | 0.14966 | 0.267094 | 0.078622 | 1 |
| B_Otu0003 | Actinomycetota | 67-14_ge | 1.46497 | 4 | 0.322684 | 0.172549 | 0.294154 | 0.045117 | 0 |
| B_Otu0005 | Actinomycetota | Streptomyces | 0.863986 | 4 | 0.5174 | 0.136223 | 0.245204 | 0.023986 | 0 |
| B_Otu0134 | Actinomycetota | Paenarthrobacter | 0.146609 | 4 | 0.287832 | 0.19469 | 0.322193 | 0.067771 | 1 |
| B_Otu0001 | Actinomycetota | Nocardioides | 1.372827 | 3 | 0.204605 | 0.207865 | 0.300912 | 0.034932 | 0 |
| F_Otu0005 | Ascomycota | Penicillium | 0.957621 | 3 | 0.066875 | 0.136646 | 0.234669 | 0.049305 | 1 |
| F_Otu0015 | Basidiomycota | Vishniacozyma | 0.889726 | 3 | 0.488812 | 0.095652 | 0.183468 | 0.015991 | 1 |
| F_Otu0049 | Ascomycota | Microdochium | 0.882265 | 3 | 0.062799 | 0.191304 | 0.266171 | 0.090425 | 0 |
| B_Otu0021 | Pseudomonadota | Skermanella | 0.456614 | 3 | 0.254734 | 0.217647 | 0.309695 | 0.042452 | 0 |
| B_Otu0054 | Actinomycetota | Pseudarthrobacter | 0.296202 | 3 | 0.114705 | 0.203704 | 0.309796 | 0.091376 | 1 |
| B_Otu0019 | Actinomycetota | Blastococcus | 0.273446 | 3 | 0.227468 | 0.203297 | 0.294713 | 0.032648 | 0 |
| F_Otu0086 | Ascomycota | Setophoma | 0.254421 | 3 | 0.027837 | 0.207865 | 0.298692 | 0.031982 | 0 |
| B_Otu0042 | Pseudomonadota | Acidibacter | 0.210028 | 3 | 0.14468 | 0.152778 | 0.255862 | 0.022654 | 0 |
| B_Otu0059 | Bacteroidota | Flavobacterium | 0.205178 | 3 | 0.228484 | 0.201087 | 0.286197 | 0.028365 | 0 |
| F_Otu0250 | Ascomycota | Albifimbria | 0.192121 | 3 | 0.229896 | 0.342857 | 0.498611 | 0.006663 | 0 |
| B_Otu0026 | Actinomycetota | Actinomycetospora | 0.182049 | 3 | 0.085048 | 0.205556 | 0.299937 | 0.032553 | 0 |
| B_Otu0068 | Actinomycetota | IMCC26256_ge | 0.181303 | 3 | 0.294392 | 0.196809 | 0.295807 | 0.02589 | 0 |
| F_Otu0083 | Chytridiomycota | Fimicolochytrium | 0.176826 | 3 | 0.421127 | 0.104513 | 0.199343 | 0.03103 | 0 |
| F_Otu0141 | Ascomycota | Lecanicillium | 0.171603 | 3 | 0.051957 | 0.387097 | 0.506944 | 0.007805 | 0 |
| F_Otu0283 | Basidiomycota | Clitopilus | 0.136537 | 3 | 0.203616 | 0.199095 | 0.284607 | 0.093851 | 0 |
| B_Otu0053 | Pseudomonadota | SC-I-84_ge | 0.130941 | 3 | 0.16 | 0.172549 | 0.282033 | 0.049496 | 0 |
| B_Otu0083 | Pseudomonadota | Candidatus_Alysiosphaera | 0.126091 | 3 | 0.26081 | 0.307692 | 0.470833 | 0.005521 | 0 |
| F_Otu0246 | Ascomycota | Niesslia | 0.119376 | 3 | 0.149832 | 0.285714 | 0.459127 | 0.003998 | 0 |
| F_Otu0003 | Mortierellomycota | Mortierella | 5.697605 | 2 | 0.245115 | 0.11828 | 0.182371 | 0.008186 | 0 |
| F_Otu0016 | Ascomycota | Alternaria | 5.03544 | 2 | 0.096565 | 0.11399 | 0.198402 | 0.037122 | 0 |
| F_Otu0031 | Ascomycota | Cladosporium | 1.078117 | 2 | 0.1364 | 0.170543 | 0.23364 | 0.079954 | 0 |
| B_Otu0006 | Pseudomonadota | Microvirga | 0.739387 | 2 | 0.221741 | 0.183168 | 0.255857 | 0.013326 | 0 |
| F_Otu0091 | Ascomycota | Scytalidium | 0.619637 | 2 | 0.02603 | 0.375 | 0.475 | 0.006663 | 0 |
| B_Otu0009 | Bacillota | Bacillus | 0.510707 | 2 | 0.049697 | 0.124646 | 0.209353 | 0.043404 | 0 |
| B_Otu0018 | Actinomycetota | Pseudonocardia | 0.473401 | 2 | 0.20718 | 0.171296 | 0.239125 | 0.006853 | 0 |
| F_Otu0052 | Ascomycota | Metarhizium | 0.468552 | 2 | 0.266735 | 0.139098 | 0.202726 | 0.006853 | 0 |
| F_Otu0124 | Basidiomycota | Entoloma | 0.431993 | 2 | 0.102844 | 0.215116 | 0.288449 | 0.033124 | 0 |
| F_Otu0009 | Basidiomycota | Solicoccozyma | 0.368947 | 2 | 0.133707 | 0.159483 | 0.231929 | 0.013326 | 0 |
| B_Otu0011 | Acidobacteriota | Bryobacter | 0.356264 | 2 | 0.085509 | 0.168582 | 0.261957 | 0.023415 | 0 |
| B_Otu0020 | Actinomycetota | Marmoricola | 0.328658 | 2 | 0.248006 | 0.174528 | 0.242632 | 0.006853 | 0 |
| F_Otu0030 | Ascomycota | Aspergillus | 0.318212 | 2 | 0.054341 | 0.133739 | 0.213835 | 0 | 1 |
| F_Otu0023 | Ascomycota | Pseudopyrenochaeta | 0.310005 | 2 | 0.083488 | 0.185 | 0.267911 | 0.019417 | 0 |
| F_Otu0069 | Ascomycota | Lectera | 0.300306 | 2 | 0.052536 | 0.16 | 0.236771 | 0.076718 | 0 |
| B_Otu0048 | Pseudomonadota | MND1 | 0.292472 | 2 | 0.127722 | 0.120879 | 0.197712 | 0.008186 | 0 |
| B_Otu0057 | Actinomycetota | Gaiella | 0.263374 | 2 | 0.09121 | 0.166667 | 0.238213 | 0.006853 | 0 |
| F_Otu0102 | Chytridiomycota | Clydaea | 0.247706 | 2 | 0.042577 | 0.166667 | 0.238213 | 0.006853 | 0 |
| B_Otu0091 | Actinomycetota | 0319-7L14_ge | 0.233903 | 2 | 0.114186 | 0.132931 | 0.20792 | 0.015991 | 0 |
| F_Otu0048 | Ascomycota | Neocosmospora | 0.196225 | 2 | 0.115063 | 0.25 | 0.387698 | 0.002094 | 0 |
| B_Otu0052 | Pseudomonadota | Haliangium | 0.189137 | 2 | 0.053111 | 0.222892 | 0.302681 | 0.035599 | 0 |
| B_Otu0096 | Actinomycetota | Aeromicrobium | 0.164515 | 2 | 0.168058 | 0.151724 | 0.240711 | 0.014468 | 0 |
| B_Otu0046 | Pseudomonadota | 0319-6G20_ge | 0.158547 | 2 | 0.129028 | 0.150685 | 0.238817 | 0.023415 | 0 |
| B_Otu0093 | Pseudomonadota | Reyranella | 0.155562 | 2 | 0.072512 | 0.205556 | 0.288042 | 0.026461 | 0 |
| B_Otu0070 | Pseudomonadota | Nitrosospira | 0.153697 | 2 | 0.32139 | 0.146667 | 0.223124 | 0.015991 | 0 |
| B_Otu0065 | Nitrospirotae | Nitrospirota | 0.152578 | 2 | 0.314501 | 0.129032 | 0.193337 | 0.008186 | 0 |
| B_Otu0035 | Actinomycetota | Geodermatophilus | 0.148847 | 2 | 0.235081 | 0.194737 | 0.272994 | 0.023986 | 0 |
| F_Otu0072 | Ascomycota | Pseudophloeospora | 0.148474 | 2 | 0.081953 | 0.181373 | 0.25755 | 0.006853 | 0 |
| B_Otu0120 | Bacteroidota | env.OPS_17_ge | 0.147355 | 2 | 0.060688 | 0.220238 | 0.292514 | 0.033981 | 0 |
| F_Otu0129 | Ascomycota | Dichotomopilus | 0.146236 | 2 | 0.090105 | 0.215116 | 0.279258 | 0.033219 | 0 |
| B_Otu0063 | Bacteroidota | Flavisolibacter | 0.145863 | 2 | 0.200872 | 0.131343 | 0.208208 | 0.008186 | 0 |
| F_Otu0198 | Ascomycota | Sarocladium | 0.138029 | 2 | 0.117015 | 0.18107 | 0.241865 | 0.08281 | 0 |
| B_Otu0069 | Gemmatimonadota | Longimicrobiaceae_ge | 0.137283 | 2 | 0.075289 | 0.352941 | 0.463889 | 0.006092 | 0 |
| B_Otu0095 | Pseudomonadota | Massilia | 0.129076 | 2 | 0.320817 | 0.164794 | 0.247563 | 0.008186 | 0 |
| F_Otu0078 | Basidiomycota | Filobasidium | 0.128329 | 2 | 0.18006 | 0.087649 | 0.153687 | 0 | 1 |
| B_Otu0101 | Pseudomonadota | Ellin6055 | 0.124226 | 2 | 0.087232 | 0.15678 | 0.219242 | 0.006853 | 0 |
| B_Otu0122 | Actinomycetota | Actinoallomurus | 0.103708 | 2 | 0.068353 | 0.087649 | 0.153687 | 0 | 1 |
| F_Otu0021 | Ascomycota | Clonostachys | 2.010744 | 1 | 0.046708 | 0.188776 | 0.246257 | 0 | 0 |
| B_Otu0007 | Actinomycetota | Mycobacterium | 0.913601 | 1 | 0.286403 | 0.120219 | 0.18256 | 0 | 0 |
| B_Otu0008 | Pseudomonadota | Sphingomonas | 0.830038 | 1 | 0.011648 | 0.164794 | 0.238093 | 0 | 0 |
| F_Otu0066 | Ascomycota | Fusariella | 0.784899 | 1 | 0.000206 | 0.285714 | 0.355556 | 0 | 0 |
| F_Otu0138 | Ascomycota | Arthrobotrys | 0.723346 | 1 | 0.073842 | 0.149194 | 0.192794 | 0 | 0 |
| B_Otu0022 | Actinomycetota | Rubrobacter | 0.697605 | 1 | 0.151506 | 0.146825 | 0.190467 | 0 | 0 |
| B_Otu0015 | Actinomycetota | Solirubrobacter | 0.647243 | 1 | 0.135447 | 1 | 1 | 0 | 0 |
| F_Otu0027 | Ascomycota | Chaetomium | 0.596508 | 1 | 0.216185 | 0.26087 | 0.348016 | 0 | 0 |
| F_Otu0036 | Ascomycota | Stemphylium | 0.444304 | 1 | 0.087347 | 0.179612 | 0.239532 | 0 | 0 |
| F_Otu0135 | Ascomycota | Pseudopithomyces | 0.424905 | 1 | 0.032835 | 0.161172 | 0.204105 | 0 | 0 |
| F_Otu0071 | Ascomycota | Darksidea | 0.370813 | 1 | 0.219224 | 0.106024 | 0.150014 | 0 | 0 |
| F_Otu0110 | Ascomycota | Chalara | 0.343953 | 1 | 0.031513 | 0.143411 | 0.189202 | 0 | 0 |
| F_Otu0014 | Ascomycota | Preussia | 0.320078 | 1 | 0.244881 | 1 | 1 | 0 | 0 |
| B_Otu0080 | Pseudomonadota | Rhodoplanes | 0.260389 | 1 | 0.244881 | 1 | 1 | 0 | 0 |
| B_Otu0034 | Gemmatimonadota | Gemmatimonas | 0.258151 | 1 | 0.022848 | 1 | 1 | 0 | 0 |
| B_Otu0079 | Actinomycetota | Kribbella | 0.253675 | 1 | 0.022848 | 1 | 1 | 0 | 0 |
| B_Otu0071 | Actinomycetota | Rhodococcus | 0.252928 | 1 | 0.022822 | 0.116402 | 0.165467 | 0 | 0 |
| F_Otu0448 | Ascomycota | Meyerozyma | 0.228307 | 1 | 0.056487 | 0.094828 | 0.153165 | 0 | 0 |
| B_Otu0098 | Actinomycetota | Actinophytocola | 0.201074 | 1 | 0.014614 | 0.108108 | 0.15869 | 0 | 0 |
| F_Otu0181 | Ascomycota | Geomyces | 0.199209 | 1 | 0.135447 | 1 | 1 | 0 | 0 |
| F_Otu0126 | Ascomycota | Periconia | 0.18466 | 1 | 0.008268 | 0.172897 | 0.223907 | 0 | 0 |
| B_Otu0142 | Actinomycetota | Glycomyces | 0.170484 | 1 | 0.000969 | 0.136029 | 0.177649 | 0 | 0 |
| B_Otu0031 | Bacillota | Sporosarcina | 0.167127 | 1 | 0.053337 | 0.154167 | 0.201299 | 0 | 0 |
| B_Otu0082 | Actinomycetota | Crossiella | 0.163769 | 1 | 0.13537 | 0.147651 | 0.214953 | 0 | 0 |
| F_Otu0109 | Ascomycota | Dokmaia | 0.159293 | 1 | 0.16146 | 1 | 1 | 0 | 0 |
| F_Otu0238 | Ascomycota | Phaeosphaeria | 0.153697 | 1 | 0.047524 | 0.188776 | 0.246257 | 0 | 0 |
| F_Otu0045 | Ascomycota | Pseudocoleophoma | 0.153324 | 1 | 0.037803 | 0.143411 | 0.189202 | 0 | 0 |
| F_Otu0425 | Ascomycota | Chrysosporium | 0.147355 | 1 | 0.06153 | 0.130564 | 0.193056 | 0 | 0 |
| B_Otu0084 | Pseudomonadota | Pseudolabrys | 0.140267 | 1 | 0.16146 | 1 | 1 | 0 | 0 |
| F_Otu0024 | Ascomycota | Plectosphaerella | 0.137656 | 1 | 0.038473 | 0.20339 | 0.293254 | 0 | 0 |
| B_Otu0192 | Bacteroidota | Chryseobacterium | 0.13206 | 1 | 0.133517 | 0.226415 | 0.324504 | 0 | 0 |
| B_Otu0090 | Actinomycetota | Conexibacter | 0.121988 | 1 | 0.136615 | 0.147651 | 0.214953 | 0 | 0 |
| F_Otu0076 | Ascomycota | Trichoderma | 0.117138 | 1 | 0.005189 | 0.171296 | 0.22469 | 0 | 0 |
| F_Otu0335 | Ascomycota | Schwanniomyces | 0.116765 | 1 | 0.016231 | 0.226415 | 0.324504 | 0 | 0 |
| B_Otu0111 | Pseudomonadota | Bradyrhizobium | 0.111915 | 1 | 0.10019 | 0.168182 | 0.217021 | 0 | 0 |
| B_Otu0074 | Acidobacteriota | Candidatus_Solibacter | 0.109304 | 1 | 0.069362 | 0.114583 | 0.158381 | 0 | 0 |
| B_Otu0127 | Gemmatimonadota | S0134_terrestrial_group_ge | 0.107066 | 1 | 0.148278 | 0.24 | 0.332143 | 0 | 0 |
| B_Otu0107 | Actinomycetota | Iamia | 0.105946 | 1 | 0.035814 | 0.132931 | 0.194068 | 0 | 0 |
| F_Otu0130 | Kickxellomycota | Ramicandelaber | 0.104081 | 1 | 0.215185 | 0.122517 | 0.165341 | 0 | 0 |
| B_Otu0116 | Bacteroidota | Pedobacter | 0.102589 | 1 | 0.167195 | 0.166667 | 0.215207 | 0 | 0 |
| F_Otu0105 | Ascomycota | Sagenomella | 0.102589 | 1 | 0.157203 | 0.141935 | 0.194336 | 0 | 0 |
| Tygerhoek KM Networks | | | | | | | | | |
| B_Otu0098 | Actinomycetota | Actinophytocola | 0.140709 | 5 | 0.259329 | 0.255102 | 0.406857 | 0.035653 | 1 |
| B_Otu0022 | Actinomycetota | Rubrobacter | 0.884458 | 4 | 0.099938 | 0.3 | 0.413624 | 0.051282 | 0 |
| F_Otu0009 | Basidiomycota | Solicoccozyma | 0.770684 | 4 | 0.084182 | 0.833333 | 0.9 | 0.002198 | 0 |
| B_Otu0034 | Gemmatimonadota | Gemmatimonas | 0.229557 | 4 | 0.245545 | 0.221239 | 0.37219 | 0.018681 | 1 |
| F_Otu0083 | Chytridiomycota | Fimicolochytrium | 1.207285 | 3 | 0.470078 | 0.297619 | 0.381333 | 0.040049 | 0 |
| B_Otu0006 | Pseudomonadota | Microvirga | 0.953204 | 3 | 0.244492 | 0.28125 | 0.366049 | 0.038828 | 0 |
| B_Otu0005 | Actinomycetota | Streptomyces | 0.862346 | 3 | 0.199965 | 0.319149 | 0.443333 | 0.014408 | 0 |
| F_Otu0021 | Ascomycota | Clonostachys | 0.65088 | 3 | 0.099367 | 0.306122 | 0.45619 | 0.013675 | 0 |
| F_Otu0049 | Ascomycota | Microdochium | 0.596205 | 3 | 0.124377 | 0.254717 | 0.362801 | 0.027839 | 0 |
| B_Otu0011 | Acidobacteriota | Bryobacter | 0.43459 | 3 | 0.083797 | 0.217742 | 0.316593 | 0.012454 | 0 |
| B_Otu0019 | Actinomycetota | Blastococcus | 0.373884 | 3 | 0.126388 | 0.214286 | 0.322413 | 0.018071 | 0 |
| B_Otu0059 | Bacteroidota | Flavobacterium | 0.336898 | 3 | 0.308519 | 0.245098 | 0.359429 | 0.021245 | 0 |
| B_Otu0091 | Actinomycetota | 0319-7L14_ge | 0.259709 | 3 | 0.113349 | 0.245902 | 0.404048 | 0.008791 | 1 |
| F_Otu0160 | Chytridiomycota | Synchytrium | 0.198601 | 3 | 0.188855 | 0.304878 | 0.396 | 0.043468 | 0 |
| F_Otu0065 | Ascomycota | Hormiactis | 0.184932 | 3 | 0.213254 | 0.264706 | 0.367945 | 0.028327 | 0 |
| B_Otu0063 | Bacteroidota | Flavisolibacter | 0.18051 | 3 | 0.294318 | 0.214286 | 0.312169 | 0.027839 | 0 |
| B_Otu0079 | Actinomycetota | Kribbella | 0.150358 | 3 | 0.167724 | 0.333333 | 0.46 | 0.01514 | 0 |
| B_Otu0142 | Actinomycetota | Glycomyces | 0.137091 | 3 | 0.274236 | 0.204918 | 0.315079 | 0.011477 | 0 |
| B_Otu0099 | Acidobacteriota | Subgroup_7_ge | 0.131865 | 3 | 0.040464 | 0.208333 | 0.367566 | 0.003419 | 1 |
| B_Otu0069 | Gemmatimonadota | Longimicrobiaceae_ge | 0.123824 | 3 | 0.18554 | 0.313953 | 0.407143 | 0.057143 | 0 |
| B_Otu0093 | Pseudomonadota | Reyranella | 0.111763 | 3 | 0.236706 | 0.245098 | 0.359429 | 0.02149 | 0 |
| B_Otu0084 | Pseudomonadota | Pseudolabrys | 0.111763 | 3 | 0.15832 | 0.290698 | 0.400095 | 0.042735 | 0 |
| F_Otu0476 | Ascomycota | Phialocephala | 0.110155 | 3 | 0.131998 | 0.18797 | 0.314254 | 0.011477 | 0 |
| F_Otu0002 | Ascomycota | Fusarium | 6.122457 | 2 | 0.042751 | 0.201613 | 0.288413 | 0.005861 | 0 |
| B_Otu0001 | Actinomycetota | Nocardioides | 2.040283 | 2 | 0.209223 | 0.18 | 0.263567 | 0.006349 | 0 |
| B_Otu0003 | Actinomycetota | 67-14_ge | 1.474632 | 2 | 0.197092 | 1 | 1 | 0.000244 | 0 |
| F_Otu0024 | Ascomycota | Plectosphaerella | 1.314626 | 2 | 0.161275 | 0.240385 | 0.312095 | 0.005861 | 0 |
| F_Otu0036 | Ascomycota | Stemphylium | 0.796414 | 2 | 0.198093 | 0.201613 | 0.288413 | 0.005861 | 0 |
| F_Otu0015 | Basidiomycota | Vishniacozyma | 0.627965 | 2 | 0.098549 | 0.277778 | 0.362 | 0.035165 | 0 |
| F_Otu0030 | Ascomycota | Aspergillus | 0.530273 | 2 | 0.155966 | 0.245902 | 0.365159 | 0.003419 | 0 |
| F_Otu0100 | Ascomycota | Gibellulopsis | 0.525046 | 2 | 0.031658 | 0.205479 | 0.334233 | 0 | 1 |
| F_Otu0135 | Ascomycota | Pseudopithomyces | 0.359411 | 2 | 0.023672 | 0.333333 | 0.426667 | 0.013675 | 0 |
| F_Otu0052 | Ascomycota | Metarhizium | 0.30353 | 2 | 0.164169 | 0.213675 | 0.318857 | 0 | 1 |
| B_Otu0071 | Actinomycetota | Rhodococcus | 0.280212 | 2 | 0.097844 | 0.160714 | 0.246208 | 0.01221 | 0 |
| B_Otu0048 | Pseudomonadota | MND1 | 0.231969 | 2 | 0.250434 | 0.283019 | 0.402857 | 0.010745 | 0 |
| F_Otu0181 | Ascomycota | Geomyces | 0.223527 | 2 | 0.131406 | 0.208333 | 0.305524 | 0.00232 | 0 |
| B_Otu0052 | Pseudomonadota | Haliangium | 0.191364 | 2 | 0.242507 | 0.211268 | 0.330265 | 0.003419 | 0 |
| F_Otu0400 | Ascomycota | Amerosporium | 0.189354 | 2 | 0.19234 | 0.214286 | 0.291902 | 0.006349 | 0 |
| F_Otu0086 | Ascomycota | Setophoma | 0.183726 | 2 | 0.118256 | 0.185185 | 0.287587 | 0.000488 | 0 |
| B_Otu0095 | Pseudomonadota | Massilia | 0.176087 | 2 | 0.074644 | 0.184932 | 0.272384 | 0.017582 | 0 |
| B_Otu0068 | Actinomycetota | IMCC26256_ge | 0.160006 | 2 | 0.222836 | 1 | 1 | 0.000244 | 0 |
| B_Otu0128 | Bacteroidota | Adhaeribacter | 0.145131 | 2 | 0.109019 | 0.245455 | 0.321693 | 0.030769 | 0 |
| F_Otu0102 | Chytridiomycota | Clydaea | 0.137091 | 2 | 0.170701 | 0.173077 | 0.246283 | 0.006349 | 0 |
| F_Otu0114 | Chytridiomycota | Rhizophlyctis | 0.131061 | 2 | 0.228632 | 0.625 | 0.7 | 0.000977 | 0 |
| F_Otu0087 | Basidiomycota | Geminibasidium | 0.12905 | 2 | 0.042093 | 0.254237 | 0.381825 | 0.006349 | 0 |
| F_Otu0051 | Basidiomycota | Cystofilobasidium | 0.120206 | 2 | 0.008749 | 0.204545 | 0.283524 | 0.01221 | 0 |
| B_Otu0053 | Pseudomonadota | SC-I-84_ge | 0.119402 | 2 | 0.154428 | 0.140625 | 0.219477 | 0.006349 | 0 |
| F_Otu0456 | Ascomycota | Aaosphaeria | 0.106939 | 2 | 0.034086 | 0.245455 | 0.328851 | 0.017582 | 0 |
| F_Otu0003 | Mortierellomycota | Mortierella | 5.259307 | 1 | 0.138099 | 1 | 1 | 0 | 0 |
| F_Otu0016 | Ascomycota | Alternaria | 3.664469 | 1 | 0.230942 | 0.177632 | 0.233495 | 0 | 0 |
| F_Otu0020 | Ascomycota | Paramyrothecium | 1.057329 | 1 | 0.001953 | 0.5 | 0.566667 | 0 | 0 |
| F_Otu0031 | Ascomycota | Cladosporium | 0.97411 | 1 | 0.015311 | 1 | 1 | 0 | 0 |
| F_Otu0153 | Ascomycota | Sarocladium | 0.95039 | 1 | 0.108951 | 0.195312 | 0.241429 | 0 | 0 |
| B_Otu0008 | Pseudomonadota | Sphingomonas | 0.883252 | 1 | 0.07917 | 0.123853 | 0.177062 | 0 | 0 |
| F_Otu0027 | Ascomycota | Chaetomium | 0.748975 | 1 | 0.000977 | 0.5 | 0.566667 | 0 | 0 |
| B_Otu0007 | Actinomycetota | Mycobacterium | 0.747367 | 1 | 0.060266 | 0.159236 | 0.23154 | 0 | 0 |
| B_Otu0015 | Actinomycetota | Solirubrobacter | 0.716009 | 1 | 0.157405 | 0.666667 | 0.75 | 0 | 0 |
| F_Otu0014 | Ascomycota | Preussia | 0.705958 | 1 | 0.243709 | 1 | 1 | 0 | 0 |
| F_Otu0081 | Ascomycota | Plenodomus | 0.578114 | 1 | 0.138099 | 1 | 1 | 0 | 0 |
| B_Otu0020 | Actinomycetota | Marmoricola | 0.498915 | 1 | 0.029756 | 1 | 1 | 0 | 0 |
| F_Otu0045 | Ascomycota | Pseudocoleophoma | 0.339712 | 1 | 0.00357 | 0.168919 | 0.224603 | 0 | 0 |
| F_Otu0218 | Ascomycota | Sporormia | 0.332878 | 1 | 0.158105 | 0.204918 | 0.278857 | 0 | 0 |
| F_Otu0069 | Ascomycota | Lectera | 0.264533 | 1 | 0.038829 | 0.204545 | 0.263771 | 0 | 0 |
| B_Otu0026 | Actinomycetota | Actinomycetospora | 0.257297 | 1 | 0.20502 | 0.153409 | 0.207497 | 0 | 0 |
| F_Otu0126 | Ascomycota | Periconia | 0.251668 | 1 | 0.11556 | 0.254237 | 0.325079 | 0 | 0 |
| B_Otu0035 | Actinomycetota | Geodermatophilus | 0.18855 | 1 | 0.021312 | 0.174419 | 0.267196 | 0 | 0 |
| F_Otu0066 | Ascomycota | Fusariella | 0.187344 | 1 | 0.112627 | 0.666667 | 0.75 | 0 | 0 |
| F_Otu0062 | Ascomycota | Articulospora | 0.180912 | 1 | 0.004975 | 0.18 | 0.236434 | 0 | 0 |
| F_Otu0078 | Basidiomycota | Filobasidium | 0.177696 | 1 | 0.275435 | 0.231481 | 0.278 | 0 | 0 |
| B_Otu0080 | Pseudomonadota | Rhodoplanes | 0.170861 | 1 | 0.188794 | 0.416667 | 0.5 | 0 | 0 |
| B_Otu0056 | Pseudomonadota | BIrii41_ge | 0.165233 | 1 | 0.243709 | 1 | 1 | 0 | 0 |
| F_Otu0091 | Ascomycota | Scytalidium | 0.162419 | 1 | 0.022264 | 0.245902 | 0.31619 | 0 | 0 |
| B_Otu0096 | Actinomycetota | Aeromicrobium | 0.152368 | 1 | 0.04644 | 0.159236 | 0.23154 | 0 | 0 |
| B_Otu0046 | Pseudomonadota | 0319-6G20_ge | 0.149152 | 1 | 0.110209 | 0.666667 | 0.75 | 0 | 0 |
| B_Otu0065 | Nitrospirotae | Nitrospirota | 0.145131 | 1 | 0.077408 | 0.18 | 0.236434 | 0 | 0 |
| B_Otu0116 | Bacteroidota | Pedobacter | 0.134679 | 1 | 0.03225 | 0.198413 | 0.261746 | 0 | 0 |
| B_Otu0120 | Bacteroidota | env.OPS_17_ge | 0.134277 | 1 | 0.206494 | 0.176471 | 0.255529 | 0 | 0 |
| B_Otu0134 | Actinomycetota | Paenarthrobacter | 0.132267 | 1 | 0.015311 | 1 | 1 | 0 | 0 |
| B_Otu0078 | Acidobacteriota | Subgroup_10 | 0.131865 | 1 | 0.054143 | 0.177632 | 0.238876 | 0 | 0 |
| F_Otu0048 | Ascomycota | Neocosmospora | 0.124628 | 1 | 0.007044 | 0.168919 | 0.224603 | 0 | 0 |
| B_Otu0107 | Actinomycetota | Iamia | 0.123422 | 1 | 0.103224 | 0.2 | 0.276931 | 0 | 0 |
| B_Otu0083 | Pseudomonadota | Candidatus_Alysiosphaera | 0.122216 | 1 | 0.025731 | 0.171233 | 0.234603 | 0 | 0 |
| F_Otu0141 | Ascomycota | Lecanicillium | 0.122216 | 1 | 0.041414 | 0.5 | 0.566667 | 0 | 0 |
| F_Otu0099 | Ascomycota | Emericellopsis | 0.120608 | 1 | 0.031092 | 1 | 1 | 0 | 0 |
| F_Otu0156 | Ascomycota | Wardomycopsis | 0.119402 | 1 | 0.031092 | 1 | 1 | 0 | 0 |
| B_Otu0090 | Actinomycetota | Conexibacter | 0.116588 | 1 | 0.039687 | 0.666667 | 0.75 | 0 | 0 |
| B_Otu0129 | Pseudomonadota | Allorhizobium-Neorhizobium-Pararhizobium-Rhizobium | 0.112165 | 1 | 0.102534 | 0.171233 | 0.234603 | 0 | 0 |
| B_Otu0110 | Pseudomonadota | Amaricoccus | 0.108949 | 1 | 0.170692 | 0.148352 | 0.197356 | 0 | 0 |
| F_Otu0299 | Ascomycota | Pyxidiophora | 0.106537 | 1 | 0.029756 | 1 | 1 | 0 | 0 |
| B_Otu0117 | Pseudomonadota | Altererythrobacter | 0.104527 | 1 | 0.054524 | 0.221311 | 0.268342 | 0 | 0 |
| B_Otu0042 | Pseudomonadota | Acidibacter | 0.104527 | 1 | 0.025209 | 0.232759 | 0.289962 | 0 | 0 |
| F_Otu0118 | Ascomycota | Sporormiella | 0.102919 | 1 | 0.041895 | 0.177632 | 0.227175 | 0 | 0 |
| Tygerhoek KC Networks | | | | | | | | | |
| F_Otu0078 | Basidiomycota | Filobasidium | 0.219841 | 5 | 0.419951 | 0.251969 | 0.383445 | 0.082418 | 2 |
| B_Otu0042 | Pseudomonadota | Acidibacter | 0.125721 | 4 | 0.376071 | 0.237037 | 0.354812 | 0.031136 | 0 |
| B_Otu0003 | Actinomycetota | 67-14_ge | 1.073784 | 3 | 0.071695 | 0.177778 | 0.287674 | 0.029637 | 0 |
| F_Otu0106 | Ascomycota | Sesquicillium | 0.995466 | 3 | 0.318664 | 0.366667 | 0.510606 | 0.006327 | 0 |
| F_Otu0019 | Basidiomycota | Solicoccozyma | 0.62895 | 3 | 0.262963 | 0.230769 | 0.358976 | 0.041958 | 0 |
| F_Otu0015 | Basidiomycota | Vishniacozyma | 0.516282 | 3 | 0.11161 | 0.22695 | 0.323363 | 0.063936 | 1 |
| F_Otu0066 | Ascomycota | Fusariella | 0.250412 | 3 | 0.207338 | 0.251969 | 0.361582 | 0.081918 | 0 |
| B_Otu0056 | Pseudomonadota | BIrii41_ge | 0.208505 | 3 | 0.145998 | 0.235294 | 0.344506 | 0.02048 | 1 |
| B_Otu0080 | Pseudomonadota | Rhodoplanes | 0.175529 | 3 | 0.103029 | 0.206452 | 0.323835 | 0.054612 | 0 |
| B_Otu0052 | Pseudomonadota | Haliangium | 0.161102 | 3 | 0.225311 | 0.366667 | 0.510606 | 0.008658 | 0 |
| B_Otu0083 | Pseudomonadota | Candidatus_Alysiosphaera | 0.155949 | 3 | 0.165197 | 0.144796 | 0.248144 | 0.038295 | 0 |
| F_Otu0049 | Ascomycota | Microdochium | 0.146675 | 3 | 0.3581 | 0.251969 | 0.352245 | 0.064436 | 0 |
| B_Otu0096 | Actinomycetota | Aeromicrobium | 0.145301 | 3 | 0.19318 | 0.235294 | 0.348885 | 0.086247 | 0 |
| F_Otu0375 | Ascomycota | Neoanthostomella | 0.145301 | 3 | 0.094581 | 0.216495 | 0.347014 | 0.034632 | 1 |
| B_Otu0091 | Actinomycetota | 0319-7L14_ge | 0.131561 | 3 | 0.1428 | 0.213333 | 0.321143 | 0.01998 | 1 |
| F_Otu0178 | Ascomycota | Wahlenbergiella | 0.121256 | 3 | 0.297998 | 0.184211 | 0.337908 | 0.027972 | 0 |
| B_Otu0046 | Pseudomonadota | 0319-6G20_ge | 0.117821 | 3 | 0.215143 | 0.458333 | 0.568182 | 0.012987 | 0 |
| F_Otu0108 | Ascomycota | Exophiala | 0.115416 | 3 | 0.049371 | 0.165354 | 0.31453 | 0.023643 | 0 |
| B_Otu0063 | Bacteroidota | Flavisolibacter | 0.105111 | 3 | 0.1603 | 0.201923 | 0.346714 | 0.02997 | 1 |
| F_Otu0016 | Ascomycota | Alternaria | 1.928414 | 2 | 0.21093 | 0.214765 | 0.294978 | 0 | 1 |
| F_Otu0045 | Ascomycota | Pseudocoleophoma | 1.825021 | 2 | 0.026235 | 0.202532 | 0.280754 | 0.058275 | 0 |
| B_Otu0022 | Actinomycetota | Rubrobacter | 0.842264 | 2 | 0.040388 | 0.15311 | 0.236445 | 0.010323 | 0 |
| B_Otu0006 | Pseudomonadota | Microvirga | 0.79177 | 2 | 0.019952 | 0.2 | 0.310074 | 0.02997 | 0 |
| B_Otu0007 | Actinomycetota | Mycobacterium | 0.786961 | 2 | 0.239076 | 0.145833 | 0.269254 | 0.012654 | 0 |
| B_Otu0008 | Pseudomonadota | Sphingomonas | 0.781121 | 2 | 0.077808 | 0.170213 | 0.250695 | 0.01998 | 0 |
| B_Otu0021 | Pseudomonadota | Skermanella | 0.556815 | 2 | 0.067102 | 0.216495 | 0.324055 | 0.032634 | 0 |
| B_Otu0009 | Bacillota | Bacillus | 0.534144 | 2 | 0.057118 | 0.366667 | 0.472727 | 0.005994 | 0 |
| F_Otu0069 | Ascomycota | Lectera | 0.468535 | 2 | 0.263466 | 0.12963 | 0.240774 | 0.00666 | 0 |
| F_Otu0052 | Ascomycota | Metarhizium | 0.458917 | 2 | 0.208987 | 0.115523 | 0.192839 | 0.010323 | 0 |
| F_Otu0153 | Ascomycota | Sarocladium | 0.401553 | 2 | 0.087827 | 0.225806 | 0.328477 | 0.035964 | 0 |
| B_Otu0018 | Actinomycetota | Pseudonocardia | 0.392278 | 2 | 0.161149 | 0.180791 | 0.259536 | 0.051948 | 0 |
| B_Otu0011 | Acidobacteriota | Bryobacter | 0.326669 | 2 | 0.146497 | 0.289474 | 0.418182 | 0.00333 | 0 |
| F_Otu0129 | Ascomycota | Dichotomopilus | 0.308807 | 2 | 0.108902 | 0.146853 | 0.274434 | 0.012654 | 0 |
| F_Otu0168 | Ascomycota | Sporormiella | 0.278236 | 2 | 0.121373 | 0.192661 | 0.293159 | 0.00666 | 0 |
| F_Otu0073 | Ascomycota | Lophiotrema | 0.254534 | 2 | 0.093212 | 0.289474 | 0.407576 | 0.00333 | 0 |
| B_Otu0071 | Actinomycetota | Rhodococcus | 0.246977 | 2 | 0.161898 | 0.161616 | 0.247497 | 0.044955 | 0 |
| F_Otu0135 | Ascomycota | Pseudopithomyces | 0.213658 | 2 | 0.056148 | 0.164062 | 0.295503 | 0.017982 | 0 |
| F_Otu0030 | Ascomycota | Aspergillus | 0.213314 | 2 | 0.109584 | 0.129032 | 0.217175 | 0.01998 | 0 |
| B_Otu0098 | Actinomycetota | Actinophytocola | 0.19133 | 2 | 0.03604 | 0.423077 | 0.507576 | 0.009324 | 0 |
| F_Otu0281 | Ascomycota | Chaetosphaeronema | 0.185147 | 2 | 0.120604 | 0.190909 | 0.299095 | 0 | 1 |
| B_Otu0034 | Gemmatimonadota | Gemmatimonas | 0.184117 | 2 | 0.035959 | 0.147465 | 0.219258 | 0.010323 | 0 |
| B_Otu0031 | Bacillota | Sporosarcina | 0.17175 | 2 | 0.255643 | 0.230769 | 0.331859 | 0.03663 | 0 |
| B_Otu0079 | Actinomycetota | Kribbella | 0.171063 | 2 | 0.044228 | 0.173913 | 0.259028 | 0.010323 | 0 |
| F_Otu0110 | Ascomycota | Chalara | 0.137744 | 2 | 0.207132 | 0.198758 | 0.287377 | 0.028971 | 0 |
| F_Otu0072 | Ascomycota | Pseudophloeospora | 0.132591 | 2 | 0.10405 | 0.130435 | 0.242988 | 0.00666 | 0 |
| B_Otu0094 | Pseudomonadota | Steroidobacter | 0.127095 | 2 | 0.093108 | 0.178771 | 0.25744 | 0.010323 | 0 |
| B_Otu0078 | Acidobacteriota | Subgroup_10 | 0.114386 | 2 | 0.173013 | 1 | 1 | 0.000333 | 0 |
| F_Otu0091 | Ascomycota | Scytalidium | 0.114042 | 2 | 0.251229 | 1 | 1 | 0.000333 | 0 |
| B_Otu0110 | Pseudomonadota | Amaricoccus | 0.113355 | 2 | 0.023411 | 0.182609 | 0.30431 | 0.02664 | 0 |
| F_Otu0002 | Ascomycota | Fusarium | 3.950605 | 1 | 0.153562 | 0.192771 | 0.254168 | 0 | 0 |
| F_Otu0020 | Ascomycota | Paramyrothecium | 2.207681 | 1 | 0.028718 | 0.142857 | 0.230783 | 0 | 0 |
| B_Otu0001 | Actinomycetota | Nocardioides | 1.835669 | 1 | 0.176686 | 0.666667 | 0.75 | 0 | 0 |
| F_Otu0031 | Ascomycota | Cladosporium | 1.52377 | 1 | 0.07639 | 1 | 1 | 0 | 0 |
| F_Otu0057 | Basidiomycota | Trechispora | 1.353394 | 1 | 0.025414 | 0.275 | 0.357576 | 0 | 0 |
| F_Otu0023 | Ascomycota | Pseudopyrenochaeta | 1.009893 | 1 | 0.186817 | 0.275 | 0.357576 | 0 | 0 |
| F_Otu0005 | Ascomycota | Penicillium | 0.82715 | 1 | 0.020436 | 0.152381 | 0.202692 | 0 | 0 |
| B_Otu0005 | Actinomycetota | Streptomyces | 0.742306 | 1 | 0.008485 | 0.148837 | 0.203633 | 0 | 0 |
| B_Otu0015 | Actinomycetota | Solirubrobacter | 0.619332 | 1 | 0.012639 | 1 | 1 | 0 | 0 |
| F_Otu0024 | Ascomycota | Plectosphaerella | 0.467848 | 1 | 0.102293 | 0.103896 | 0.156447 | 0 | 0 |
| F_Otu0062 | Ascomycota | Articulospora | 0.449643 | 1 | 0.171822 | 0.156716 | 0.245503 | 0 | 0 |
| F_Otu0027 | Ascomycota | Chaetomium | 0.422506 | 1 | 0.07639 | 1 | 1 | 0 | 0 |
| F_Otu0141 | Ascomycota | Lecanicillium | 0.353806 | 1 | 0.112006 | 0.162791 | 0.227779 | 0 | 0 |
| F_Otu0134 | Ascomycota | Torula | 0.32873 | 1 | 0.074543 | 0.666667 | 0.75 | 0 | 0 |
| F_Otu0109 | Ascomycota | Dokmaia | 0.246634 | 1 | 0.150582 | 0.126984 | 0.188529 | 0 | 0 |
| B_Otu0054 | Actinomycetota | Pseudarthrobacter | 0.21194 | 1 | 0.097721 | 0.275 | 0.357576 | 0 | 0 |
| F_Otu0128 | Ascomycota | Dactylonectria | 0.208162 | 1 | 0.073954 | 1 | 1 | 0 | 0 |
| F_Otu0326 | Ascomycota | Xylaria | 0.184804 | 1 | 0.027795 | 0.129032 | 0.177539 | 0 | 0 |
| F_Otu0083 | Chytridiomycota | Fimicolochytrium | 0.178277 | 1 | 0.174929 | 0.192771 | 0.254168 | 0 | 0 |
| B_Otu0048 | Pseudomonadota | MND1 | 0.150453 | 1 | 0.136507 | 0.229167 | 0.314502 | 0 | 0 |
| B_Otu0068 | Actinomycetota | IMCC26256_ge | 0.148049 | 1 | 0.020667 | 0.151659 | 0.215612 | 0 | 0 |
| B_Otu0057 | Actinomycetota | Gaiella | 0.147362 | 1 | 0.017883 | 0.133333 | 0.187882 | 0 | 0 |
| F_Otu0124 | Basidiomycota | Entoloma | 0.142896 | 1 | 0.038502 | 0.115385 | 0.19094 | 0 | 0 |
| F_Otu0167 | Ascomycota | Vaginatispora | 0.125721 | 1 | 0.017569 | 0.666667 | 0.75 | 0 | 0 |
| B_Otu0116 | Bacteroidota | Pedobacter | 0.11576 | 1 | 0.012639 | 1 | 1 | 0 | 0 |
| F_Otu0138 | Ascomycota | Arthrobotrys | 0.105455 | 1 | 0.155444 | 0.666667 | 0.75 | 0 | 0 |
| B_Otu0093 | Pseudomonadota | Reyranella | 0.105111 | 1 | 0.073954 | 1 | 1 | 0 | 0 |
| B_Otu0095 | Pseudomonadota | Massilia | 0.104081 | 1 | 0.048171 | 0.229167 | 0.309957 | 0 | 0 |
| B_Otu0183 | Pseudomonadota | Novosphingobium | 0.103394 | 1 | 0.150246 | 0.202532 | 0.256969 | 0 | 0 |
| B_Otu0129 | Pseudomonadota | Allorhizobium-Neorhizobium-Pararhizobium-Rhizobium | 0.101676 | 1 | 0.006464 | 0.116022 | 0.192127 | 0 | 0 |
